# Unraveling the Reactivity of SiO_2_-Supported Nickel Catalyst in Ethylene Copolymerization with Polar Monomers: A Theoretical Study

**DOI:** 10.3390/polym17091268

**Published:** 2025-05-06

**Authors:** Daniela E. Ortega, Diego Cortés-Arriagada

**Affiliations:** 1 Centro Integrativo de Biología y Química Aplicada (CIBQA), Universidad Bernardo O’Higgins, General Gana 1702, Santiago 8370854, Chile; 2 Instituto Universitario de Investigación y Desarrollo Tecnológico (IDT), Universidad Tecnológica Metropolitana, Ignacio Valdivieso 2409, San Joaquín, Santiago 8940577, Chile; dcortes@utem.cl

**Keywords:** ethylene copolymerization, SiO_2_-supported catalyst, polar monomers, nickel catalyst, DFT calculations

## Abstract

Understanding the catalytic behavior of heterogeneous systems for the copolymerization of ethylene with polar monomers is essential for developing advanced functional polyolefins. In this study, we conducted a quantum chemical investigation of the SiO_2_-supported Ni–allyl–α-imine ketone catalyst (Ni-OH@SiO_2_) to uncover the factors governing monomer insertion, selectivity, and reactivity. Using DFT calculations and energy decomposition analysis (ALMO-EDA), we evaluated the coordination and insertion of six industrially relevant polar monomers, comparing their behavior to ethylene homopolymerization. Our results show that special polar monomers (SPMs) with aliphatic spacers, such as vinyltrimethoxysilane (vTMS) and 5-hexenyl acetate (AMA), exhibit favorable insertion profiles due to enhanced electrostatic and orbital interactions with minimal steric hindrance. In contrast, fundamental polar monomers (FPMs), including methyl acrylate (MA) and vinyl chloride (vCl), show higher activation barriers and increased Pauli repulsion due to strong electron-withdrawing effects and conjugation with the vinyl group. AMA displayed the lowest activation barrier (7.4 kcal/mol) and highest insertion thermodynamic stability (−17.6 kcal/mol). These findings provide molecular-level insight into insertion mechanisms and comonomer selectivity in Ni–allyl catalysts supported on silica, extending experimental understanding. This work establishes key structure–reactivity relationships and offers design principles for developing efficient Ni-based heterogeneous catalysts for polar monomer copolymerization.

## 1. Introduction

Ethylene polymerization is a highly efficient and widely adopted industrial process conducted on a large scale expected to hit USD 163 billion by 2032 [1,2]. Polyethylene (PE), recognized as one of the most extensively utilized polymers, is crucial in everyday life owing to its exceptional mechanical properties, durability, versatility, and cost-effectiveness [3]. Despite these attributes, PE’s inherent hydrocarbon-based chemical structure produces less-than-optimal adhesion to polar materials, limiting its potential applications. Introducing polar functional groups into the PE monomer offers a pathway to craft polyolefins with distinct microstructures and properties, ultimately enhancing adhesion, wettability, and miscibility [4]. In the industrial realm, two predominant methods produce functionalized PE: postpolymerization functionalization and free radical copolymerization of ethylene with various polar monomers [5]. However, postpolymerization functionalization often requires stringent reaction conditions, while the free radical copolymerization process grapples with challenges related to imprecise control over copolymer composition and microstructure [6,7]. An alternative method involves metal-catalyzed coordination copolymerizing ethylene with polar monomers [8], offering a direct and economically viable route to produce functionalized PE. A key advantage lies in the coordination−insertion mechanism, where the structure of the metal catalyst predominantly dictates the copolymerization process [9,10,11,12,13,14]. This approach operates under mild reaction conditions, minimizing undesirable side reactions such as cross-linking and degradation.

**Scheme 1:**
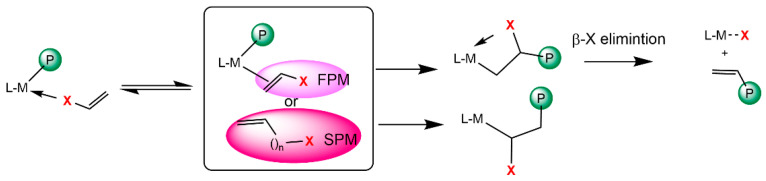
Influence of the polar monomer (FPM, SPM) in the olefin polymerization process. M: metal, L: ligand, P: polymer, X: polar functional group.

Several challenges emerge in the polar olefin copolymerization mechanism (Scheme 1); after the insertion of the polar monomer (X: polar functional group), subsequent insertion of the new monomer becomes challenging due to the presence of the X group, favoring the formation of stable X compounds (LM⋯X) and fast chain termination reactions, such as β-X elimination, which are crucial factors to control [12]. Two types of polar monomers (Scheme 1) further complicate the scenario; fundamental polar monomers (FPM) have the polar functional group directly attached to the C–C double bond, making them challenging substrates due to factors such as fast β-X chain termination reactions, prevention of comonomer binding by the X group binding to the metal center and inactivating the complex, and rate retardation of polymerization. However, their significance in industrial applications, such as polyvinyl chloride (PVC) production, cannot be overlooked. Conversely, special polar monomers (SPM) feature a spacer between the polar group and the double bond, making it relatively easier to copolymerize with ethylene since the polar group’s presence is remote from the metal center. Therefore, the synthetic strategy for rationalizing catalysts for the more direct and economical copolymerization of polar olefins remains a significant challenge and is considered one of the last “Holy Grails” in this field [15].

Consequently, transition-metal-catalyzed ethylene−polar monomer copolymerization has garnered increasing attention in recent years. However, this method has not yet been applied in the industry. Mainly because heterogeneous molecular catalysts based on early transition metals (Ti, Zr, or Cr) widely used in the industry suffer from the poisoning effects of polar groups toward metal centers [16]. In this context, heterogeneous catalysts based on late-transition metals like nickel have shown that they can directly copolymerize ethylene with polar functional groups [17]. This capability stems from the nickel catalyst’s less oxophilic nature than many early transition-metal catalysts [10,11,12,13,14]. In addition, their efficiency, earth abundance, and low cost make them more attractive alternatives than their high-cost Pd and Pt counterparts [18]. Notwithstanding, there has been no important commercialization in the copolymerization industry, where the design and investigation of high-performance heterogeneous catalysts to facilitate the synthesis of polar polymers represents a pivotal industry motivation for engaging in this research domain.

**Scheme 2:**
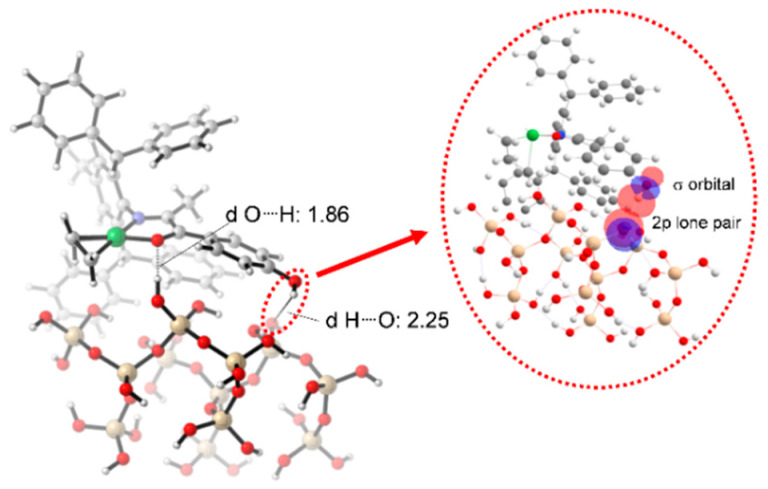
Heterogenization of the Ni–allyl–α-imine ketone catalyst by OH as a linker group on the SiO_2_ surface (Ni-OH@SiO_2_). Chemisorption of the Ni–α-imine ketone catalyst with the SiO_2_ support is evidenced by the interaction of the low-occupied σ-orbital belonging to the -OH linker group with the 2p lone pair electrons belonging to the oxygen atom from the silanol group of the support according to our previous results [21]. Atom color code: oxygen (red), nitrogen (blue), nickel (green), silicon (yellow), carbon (gray), hydrogen (white).

The heterogenization of molecular catalysts with promising characteristics can be a good alternative for the industry since it can bring together all the advantages of homogeneous and heterogeneous catalysts in a single system [19]. One of the plausible heterogenization approaches for olefin polymerization catalysts can be achieved by immobilizing onto solid support by covalent and non-covalent interactions such as electrostatic and Van der Waals interactions, maximizing the polymerization process’s stability, activity, and selectivity [17,20]. In this work, we have focused on heterogenization by locating the OH moiety as a linker group on the backbone of an *α*-imino-ketone-based [Ni(allyl)]^+^ catalyst that allows the adsorption of the catalyst by covalent interaction with the SiO_2_ support (Ni-OH@SiO_2_), as shown in Scheme 2 [21]. Ni-OH@SiO_2_ catalyst has demonstrated high thermal stability (operation temperature > 100 °C) [22], which makes it extremely attractive for industrial applications. Additionally, its heterogeneous nature allows for physical separation and potential recovery after reaction, minimizing environmental risks such as nickel leaching. Liang et al. [22] showed that this catalyst maintains its activity under operational conditions and can be easily separated from the polymer product due to its particulate morphology. Its performance in copolymerization is also notable: the homogeneous counterpart exhibits moderate activity (0.3 × 10^4^ g PE/mol Ni h) and copolymer molecular weight (17,900 g/mol) under mild conditions without the need for additional cocatalysts using FPM. Conversely, utilizing SPM, it displays high activity (13.3 × 10^4^ g PE/mol Ni h), significant incorporation (10.8%), and high molecular weight (165,200 g/mol), showcasing its superior capability to facilitate the production of polar functionalized linear low-density polyethylene (LLDPE) [22]. The latter suggests a lower poisoning effect of this type of catalyst in copolymerization reactions involving polar monomers.

**Scheme 3:**
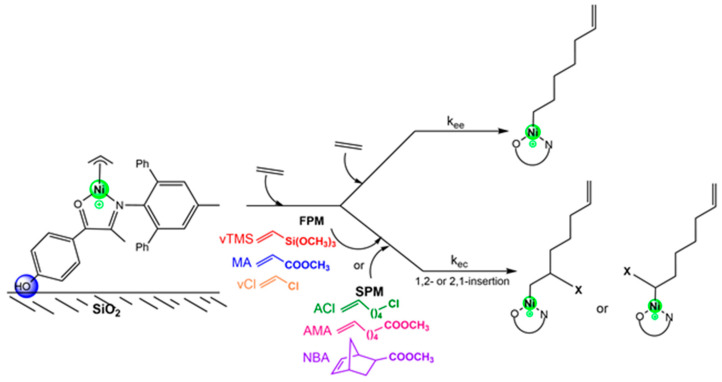
Insertion modes in ethylene copolymerization reactions on Ni-OH@SiO_2_ catalyst. The initial Ni catalyst contains a pre-coordinated allyl ligand; subsequent monomer insertions (ethylene or FPM- or SPM-type comonomers) increase the carbon count accordingly.

Considering the above, we performed a novel quantum chemical investigation aimed at characterizing the reactivity of the Ni-OH@SiO_2_ catalyst for ethylene copolymerization reactions. The utilization of heterogeneous catalysts for the production of polar functionalized polyolefins remains largely unexplored, thus offering an intriguing avenue for research. We aim to address the development of efficient heterogeneous molecular catalysts for ethylene copolymerization with polar monomers. These challenges include the lack of sufficient mechanistic understanding, making it difficult to study the structure–reactivity relationship, and the unclear origin of the polar comonomer effect on the reactivity of heterogeneous catalysts. Consequently, a thorough analysis of the factors stabilizing the transition state can provide a theoretical foundation for the rational selection of parameters that ligands interacting with specific monomers, comonomers, and surfaces should possess. Additionally, we examine the substrate effects through copolymerization reactions with FPM and SPM comonomers based on experimental data from their homogeneous counterparts [22], including vinyltrimethoxysilane (vTMS), 5-norbornene-2-yl acetate (NBA), methyl acrylate (MA), and vinyl chloride (vCl). The SPMs are based on an MA and vCl longer chain; we will refer to them as alkyl methacrylate (AMA; 5-hexenyl acetate) and alkenyl chloride (ACl; 6-chloro-1-hexene) (Scheme 3). While these polar-functionalized polyolefins may pose challenges to traditional recycling methods, their tunable architectures and functional surface groups make them suitable for reuse in high-performance composites. Zou et al. [17] demonstrated that such materials can be successfully integrated into advanced applications, including reinforced composites and additive manufacturing, thereby offering pathways for functional recycling and reducing dependency on energy-intensive end-of-life treatments. The homogeneous Ni–α-imine ketone catalyst provides a low-pressure (8 atm of ethylene), low-temperature (20–50 °C) pathway for the copolymerization process. Its heterogeneous counterpart could offer an alternative to commercially available copolymerization methods, which are typically produced by radical polymerizations in high-pressure reactors [23].

## 2. Computational Methods

A 2 × 2 × 1 supercell based on the α-quartz (α-SiO_2_) crystallographic model [24] with (001) crystalline orientation was used to build a slab model consisting of 12 molecular units with a thickness of 5.1 Å in order to construct a closed-shell finite cluster (Si_12_O_36_H_26_) on the basis of DFT calculations with the Crystal17 package [25], as implemented in our previous studies [21]. Geometries of all stationary points were optimized using the ω-B97XD/6-31+G(d,p) level of theory in Gaussian 16 [26,27,28], considering the SiO_2_ support fixed with the exception of the top silanol (SiOH) and the siloxane (SiOSi) groups that interact with the OH linker group of the catalyst in order to avoid the cluster deformation due to the size effect. The conformational search for the coordination of all monomers in either *cis* or *trans* positions relative to the oxygen atom (ketone moiety) was conducted using the Adsorption Locator module in BIOVIA Materials Studio, employing a Monte Carlo-based simulated annealing method with the universal force field (UFF) [29,30]. A total of 20 conformations were screened, and the three most stable were fully optimized using the same level of theory in Gaussian 16. Furthermore, the 1,2- and 2,1-insertion modes of polar monomers were explored through conformational sampling with the Conformers module in BIOVIA Materials Studio [31], utilizing the Boltzmann jump search method. [30] The most stable low-energy conformers were subsequently optimized using the same method in Gaussian 16 and were reported in Table S1.

To establish the connection between the reactants and products, we employed the nudged elastic band + saddlepoint-minimization (NEB-TS) method [32] within ORCA 5.0.1 [33]. This approach entailed generating ten intermediate images between both minima as part of the transition state structure search process. To confirm the nature of the optimized structures, vibrational frequency calculations were also performed; all the optimized transition state structures exhibit only one imaginary frequency, while all minima display no imaginary frequencies. Truhlar’s quasi-harmonic corrections, in which all vibrational frequencies below 100 cm^−1^ were shifted to 100 cm^−1^ in entropy calculations, were performed in the GoodVibes package [34]. DLPNO-CCSD(T)/def2-SVP [35,36] single-point energy calculations were carried out with the SMD solvation model in toluene as solvent (ε = 2.37) [37,38]. Reported Gibbs free energies in solution include thermal corrections computed at 298 K and were corrected with this method.

Energy decomposition analysis (EDA) calculations were performed to decompose the activation energies into the energy difference between the transition state species and the ground state π-complexes (∆ΔE^ǂ^ = ∆E_TS_ − ∆E_π-complex_). In this context, for each species, EDA was computed in terms of the interaction energy (ΔE_INT_) between the monomers and the catalyst using the absolutely localized molecular orbital method (ALMO-EDA-2) in Q-Chem 6.1 [39,40]. This approach allows for the dissection of ΔE_INT_ into chemically significant components, such as electrostatic Coulombic interactions (ΔE_ELEC_), orbital interactions (ΔE_ORB_ = ΔE_CT_ + ΔE_POL_) involving interfragment charge transfer (ΔE_CT_) between the catalyst and the monomers and the intrafragment polarization energies (ΔE_POL_), dispersion interactions (ΔE_DISP_), and the Pauli repulsion (ΔE_PAULI_) effects:

ΔE_INT_ = ΔE_ELEC_ + ΔE_ORB_ + ΔE_DISP_ + ΔE_PAULI_ (1)

Natural bond orbital (NBO) analyses were performed to characterize σ and π-interactions between the monomers and the nickel catalyst by employing the second-order Fock matrix and the stabilization energy [E(2)] [41], which indicates the strength of interactions between electron donors and acceptors. Moreover, the electronic population analysis will also be carried out for insight into the charge distribution on the Ni centers by using the CM5 atomic charges [42] in the Multiwfn 3.8 package [43].

## 3. Results and Discussions

**Figure 1:**
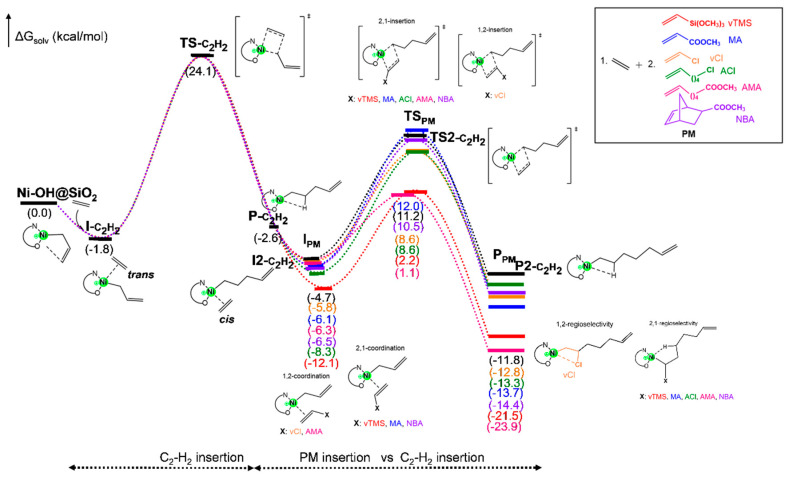
Free energy profiles for ethylene homopolymerization and copolymerization with polar monomers catalyzed by the Ni-OH@SiO_2_ system.

The reaction mechanism for the ethylene copolymerization with polar monomers (PM) was studied based on the chain initiation and propagation steps by ethylene insertion followed by PM insertion. The preferential insertion of ethylene is supported by its lower steric hindrance and higher intrinsic reactivity under mild conditions, as evidenced by experimental studies [22,44,45]. In contrast, polar comonomers typically exhibit reduced reactivity and tend to suppress catalytic activity, particularly at early stages, due to electronic and steric constraints. Therefore, two main precursors are involved in the reaction pathway. These are (i) the Ni–allyl complex (Figure 1; Ni-OH@SiO_2_) and (ii) the Ni–alkyl complex, which is the catalytically active species formed after the insertion of the first monomer (ethylene) into the Ni–allyl precursor (Figure 1; P_C2H2_). This species serves as the reference point for subsequent monomer insertions. To rationally characterize the activity of heterogenization of Ni–α-imino ketone molecular catalyst for ethylene copolymerization reactions, the Gibbs free energy profile is discussed below (Figure 1). We first investigated the ethylene insertion into the Ni-OH@SiO_2_, followed by the PM insertion into the P_C2H2_ species, as is shown in Figure 1. From our previous studies [21,46], we found that the ethylene uptake is more stable *trans* to the ketone moiety (monomer *trans* to the O atom) by almost 2.0 kcal/mol (I_C2H2_). Then, the ethylene insertion (TS_C2H2_) has a barrier of 25.9 kcal/mol exergonically, forming a stable β-agostic complex P_C2H2_. We further investigated the polar monomer (PM) insertion vs. the ethylene insertion in the P_C2H2_ species.

### 3.1. Insights into the Feasibility of Incorporation of PMs into Ni-OH@SiO_2_ Catalyst

**Figure 2:**
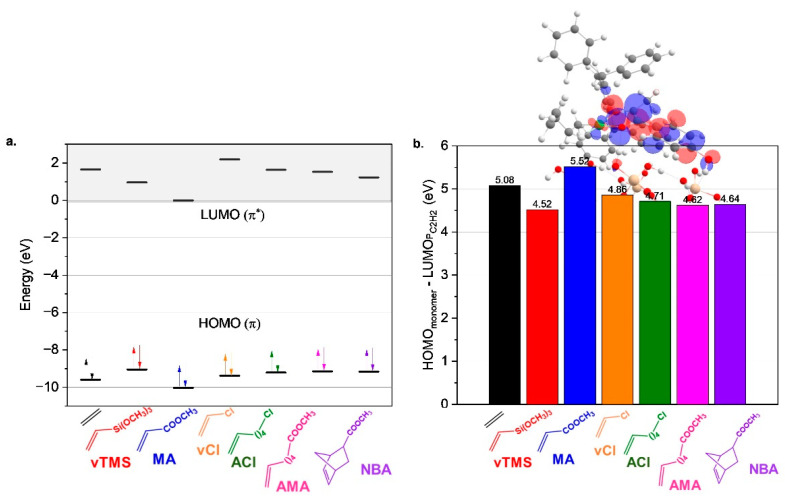
(**a**) Frontier orbital energy levels (HOMO and LUMO) of the monomers analyzed in this study. (**b**) HOMO-LUMO energy difference between the HOMO of each monomer and the LUMO of the alkyl product (P2-_C2H2_) formed after the first ethylene insertion, as depicted in Figure 1. Atom color code: oxygen (red), nitrogen (blue), nickel (green), silicon (yellow), carbon (gray), hydrogen (white).

A total of six polar monomers (PMs) were investigated for ethylene copolymerization (Figure 1). The monomer coordination properties were predicted based on the energies of the HOMO (π C=C bonding orbital) and LUMO (π* C=C antibonding orbital) of ethylene and the polar monomers (Figure 2a). The HOMO energies of the polar monomers differ significantly from that of ethylene, exhibiting higher values (−9.05 to −9.38 eV vs. −9.60 eV), with the notable exception of methyl acrylate (MA), which has the lowest HOMO energy (−10.03 eV). This suggests that most polar monomers exhibit stronger σ-donation to the catalyst, forming stronger carbon–metal π-bonds during the copolymerization process. Among the monomers, vinyltrimethylsilane (vTMS) is predicted to show the strongest π-complexation due to its highest HOMO energy (−9.05 eV), whereas MA exhibits the weakest coordination. Vinyl chloride (vCl), with a HOMO energy of −9.38 eV, is predicted to have the second weakest performance after MA. For monomers with similar HOMO energies, such as alkyl chloride (ACl), alkyl methacrylate (AMA), and norbornene acetate (NBA) (~−9.2 eV), comparable σ-donation to the metal center is anticipated.

Insights into the back-donation from the catalyst to the monomer were obtained from the LUMO energies of the monomers (Figure 2a), which is critical for stabilizing the π-complex. MA has the lowest LUMO energy (0.01 eV), indicating it could facilitate substantial back-donation compared to other polar monomers, whose LUMO energies range between 0.97 and 2.19 eV. Particularly, vCl has the highest LUMO energy (2.19 eV), predicting the weakest back-donation. The back-donation trend is as follows: vCl (2.19 eV) < ethylene (1.66 eV) ≈ ACl (1.64 eV) < AMA (1.53 eV) < NBA (1.23 eV) < vTMS (0.97 eV). This trend highlights that vTMS is expected to exhibit the most favorable back-donation among the polar monomers.

In addition to these insights, we analyzed the interaction between the HOMO and LUMO of the monomers and alkyl product formed after the first ethylene insertion (P_C2H2_), respectively. Figure 2b shows that the LUMO of P_C2H2_ is mainly localized on the nickel center and the imine ketone ligand, providing an important descriptor of coordination strength. The HOMO-LUMO energy difference in MA is the largest (5.52 eV), indicating the most challenging coordination. In comparison, the HOMO-LUMO energy difference of vTMS is the smallest (4.52 eV), suggesting the easiest coordination among the polar monomers. The gaps for the other monomers follow the trend: ethylene (5.08 eV) > vCl (4.86 eV) > ACl (4.71 eV) > AMA ≈ NBA (~4.60 eV) > vTMS (4.52 eV). The study predicts that vTMS will have the strongest coordination properties among polar monomers due to favorable HOMO and LUMO energies, while MA will have weak coordination behavior. However, all polar monomers are predicted to coordinate more effectively with the catalyst than ethylene, except for MA, which is consistent with its low incorporation experimentally observed in supported Ni-based catalysts, such as in the study by Zhang et al. [44], where MA incorporation was limited to ~0.5–0.7 mol%. This highlights the diverse electronic properties of the polar monomers and their potential to influence the copolymerization process.

Lastly, we have examined the coordination of polar monomers (PMs) into P_C2H2_ (I_PM_ species in Figure 1). The coordination of the PMs taking place at the *cis* site of the ketone moiety consistently yielded more stable π-complexes. Consequently, this study focused exclusively on the favorable *cis* insertion of monomers. Among the polar monomers, comparative energy data are reported in Table S1, in which it is shown that the 1,2-coordination mode to the nickel center was preferred for vCl and AMA comonomers. Interestingly, despite AMA forming a 1,2-coordinated π-complex, the transition state for insertion is more stable in the 2,1 orientation. In contrast, the 2,1-coordination mode was favored for vTMS, MA, ACl, and NBA. The formation of I_PM_ species was exergonic for all PMs (Figure 1). These findings are consistent with the experimental evidence [47], in which MA undergoes 2,1-insertion exclusively, whereas other polar monomers like vCl insert via the 1,2 pathway. Notably, all I_PM_ species exhibited more exergonic incorporation than the ethylene monomer, which is consistent with our previous HOMO-LUMO analysis (Figure 2b) and reflects their enhanced thermodynamic favorability.

Experimentally, the homogeneous counterpart of the Ni-OH@SiO_2_ catalyst demonstrated higher incorporation rates of special polar monomers (SPMs) such as AMA (6.3%), NBA (5.5%), and ACl (3.4%) compared to FPMs (functionalized polar monomers) such as MA (0.6%), with vTMS showing the highest incorporation (10.8%) [22]. Consistent with these findings, our calculations predict the following order of comonomer incorporation based on Gibbs free energy changes:

vTMS (−9.5 kcal/mol) > ACl (−5.7 kcal/mol) > NBA (−3.9 kcal/mol) > AMA (−3.7 kcal/mol) > MA (−3.5 kcal/mol) > vCl (−3.2 kcal/mol). While NBA, AMA, MA, and vCl exhibit similar ΔG° values, subtle differences in their functional group properties influence their incorporation. For example, variations in inductive effects, electron delocalization, and steric hindrance impact the monomer’s interaction with the catalyst.

**Figure 3:**
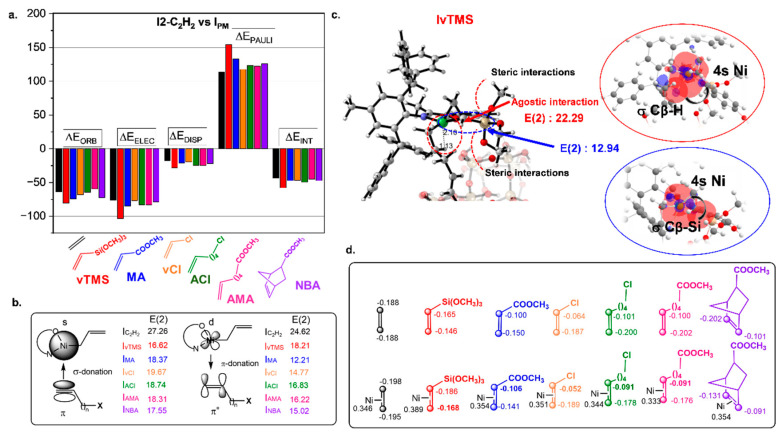
(**a**) Comparison of the interaction energies for ethylene and polar monomer incorporation into the π-complexes of the Ni-OH@SiO_2_ catalyst, based on energy decomposition analysis (EDA). (**b**) Natural bond orbital (NBO) second-order perturbation energy analysis [E(2)], highlighting σ-donation and π-donation interactions between the Ni-OH@SiO_2_ catalyst and each monomer in the π-complex. Energies are reported in kcal/mol. (**c**) Representation of the vTMS π-complex, showing Cβ-H and Cβ-Si moieties, serves as donors to the nickel center. (**d**) CM5 atomic charges of the monomers, both in their isolated states and within the π-complexes. Atom color code: oxygen (red), nitrogen (blue), nickel (green), silicon (yellow), carbon (gray), hydrogen (white).

To elucidate the origin of monomer incorporation, we conducted an ALMO-EDA study according to Equation (1), revealing the nature of catalyst-monomer interactions in the π-complexes (Figure 3a). The I_vTMS_ complex emerged as the most thermodynamically favorable intermediate, with the highest interaction energy (ΔE_INT_ = −57.9 kcal/mol). This interaction is predominantly governed by electrostatic (ΔE_ELEC_ = −103.4 kcal/mol) and orbital (ΔE_ORB_ = −80.6 kcal/mol) contributions, with Pauli repulsion (ΔE_PAULI_ = 154.5 kcal/mol) and dispersive effects (ΔE_DISP_ = −28.4 kcal/mol) playing relatively smaller roles. A similar trend was observed for other PMs, where electrostatic and orbital interactions were the primary contributors to the catalyst–monomer binding.

NBO analysis further supports this conclusion, highlighting donor-acceptor interactions in terms of stabilization energy [E(2)] (Figure 3b). The polarization of the vinyl groups was assessed through CM5 atomic charges (Figure 3d), which revealed that the polar groups induce an asymmetric π-electron distribution, as reflected in the following order of polarization: vTMS (Δe = 0.018 e) < MA (Δe = 0.035 e) < NBA (Δe = 0.040 e) < AMA (Δe = 0.085 e) < ACl (Δe = 0.087 e) < vCl (Δe = 0.137 e). For most monomers (e.g., MA, vCl, ACl, AMA, NBA), the vinyl carbons become less negative upon π-complex formation, indicating stronger σ-donation to the nickel center [higher E(2) values for σ-donation compared to π-donation]. This behavior underscores the role of the metal as an electron acceptor, stabilizing the π-complex and priming the monomer for subsequent insertion.

For instance, the highest polarization observed in the I_vCl_ complex is due to stronger σ-donation [E(2) = 19.67 kcal/mol] compared to π-donation [E(2) = 14.77 kcal/mol]. This behavior is attributed to the strong electron-withdrawing inductive effect of chlorine, which reduces electron density on the vinyl carbon and alters the distribution of orbital interactions. The presence of the electronegative chlorine atom enhances the asymmetry of the π-system, increasing its overall polarizability and favoring σ-type interactions with the Ni center. A similar trend is noted in ACl, where the longer spacer weakens the vinyl carbon polarization (0.087 e vs. 0.137 e for vCl) but promotes stronger π-donation [E(2) = 16.83 kcal/mol].

For AMA (Δe = 0.085 e), the ester group contributes moderate electronegativity and polarizability, facilitating electron delocalization. In comparison, MA shows a lower degree of polarization (Δe = 0.035 e) due to its simpler structure. In NBA, the slightly greater polarization (Δe = 0.040 e) relative to MA can be attributed to its rigid structure, which confines electron density and concentrates it around the vinyl and ester groups. This promotes efficient electronic interactions, leading to stronger π-donation from the nickel center to the NBA π* orbital (E(2) = 15.02 kcal/mol) compared to MA (E(2) = 12.21 kcal/mol).

In the I_vTMS_ complex, an agostic interaction was identified between the nickel center and the β-hydrogen of the already inserted chain (Figure 3c). This interaction arises from the overlap between the σ Cβ−H orbital and the nickel 4s orbital [E(2) = 22.29 kcal/mol], which contributes to stabilizing the π-complex. However, the observed Ni–Hβ distance of 2.10 Å is significantly longer than typical agostic distances (1.7–1.8 Å), suggesting that the interaction between the monomer and the nickel center partially suppresses optimal charge transfer where the nickel center exhibits high electrophilicity (+0.389 e). In addition to the agostic effect, a stabilizing σ(C–Si) → Ni electron-donating interaction is also observed [Figure 3c; E(2) = 12.94 kcal/mol], originating from the SiMe_3_ substituent of vTMS. While this σ-donation would generally be expected to reduce the electrophilicity of the metal center, it plays a nuanced role in enhancing the overall stability of the π-complex without significantly altering the nickel’s Lewis acidic nature. The bulky and polarizable SiMe_3_ group contributes not only via dispersion forces but also by promoting local orbital complementarity through σ-donation, which facilitates a preorganized geometry favorable for subsequent monomer insertion.

Interestingly, the vinyl group of vTMS exhibits an increase in negative charge when forming the π-complex, compared to its isolated monomer state (Figure 3d). This behavior underscores a critical aspect of the electronic stabilization within the complex. The redistribution of electron density is facilitated by the nickel center, as reflected in a greater E(2) value for π-donation compared to σ-donation (Figure 3b). The nickel center not only stabilizes the monomer through coordination but also mediates a transfer of electron density from its d-orbital to the π* orbital of the vinyl group. This interaction exemplifies the metal’s dual role: acting as a stabilizing anchor and promoting charge redistribution to strengthen the complex. Unlike polar monomers containing more electronegative or polarizable substituents, the electronic environment of vTMS enables a unique redistribution of charge under the influence of the nickel center. Furthermore, while the agostic interaction is intramolecular (not directly involving vTMS), it may alter the catalyst’s electronic properties. This adjustment could indirectly influence both the stabilization and the charge redistribution mechanisms within the π-complex.

A comparative analysis of the interaction energy components between the π-complexes formed with ethylene and various polar monomers was performed using ALMO-EDA (see Figure S1). All comonomers exhibited more favorable interaction energies than the ethylene-only π-complex, indicating enhanced thermodynamic stabilization upon coordination. Among the destabilizing factors, Pauli repulsion was found to contribute significantly (32–45%), particularly for MA, NBA, and vTMS, consistent with their bulkier structures or electron-withdrawing groups. These effects contrast with ethylene’s smaller size and symmetrical geometry, which facilitate more effective orbital overlap with the Ni center and reduced steric disruption. A detailed breakdown of these contributions, including electrostatic and orbital effects, is provided in the Supporting Information (Figure S1).

In summary, vTMS exhibited the highest incorporation and thermodynamic favorability among the studied polar monomers, driven by strong electrostatic and orbital interactions (Figure 3a). Differences in functional group properties—such as inductive effects and polarizability—significantly influenced coordination strength and complex stability. vTMS, with its electron-rich alkoxy substituents, enhances orbital overlap with the metal, whereas MA, featuring an electron-withdrawing ester group, increases Pauli repulsion and weakens binding, highlighting the contrasting electronic effects that govern monomer–catalyst interactions. Compared to ethylene, the polar monomers introduce distinct electronic and steric characteristics that modulate the stability and binding efficiency of the π-complexes. Ethylene, due to its smaller size and symmetry, exhibits superior orbital overlap with the nickel center, resulting in stronger σ-donation and π-back-donation interactions (Figure 3b), which facilitates an effective coordination process. In contrast, polar monomers, despite their enhanced electrostatic contributions, experience greater steric hindrance and electronic polarization at the nickel center. The balance between these factors underpins the competitive incorporation of polar monomers in copolymerization processes. Notably, the ability of vTMS to achieve high incorporation despite these challenges highlights its unique electronic and steric properties, making it a promising candidate for functionalized copolymer synthesis.

### 3.2. Activation Barrier Analysis for Polar Monomer Insertion into Ni-OH@SiO_2_ Catalyst

The insertion of ethylene as the second monomer (TS2_-C2H2_) presents an energy barrier of 15.9 kcal/mol, leading to the formation of an exergonic β-agostic complex, P2_C2H2_ (∆G° = −7.1 kcal/mol) as shown in Figure 1.

**Figure 4:**
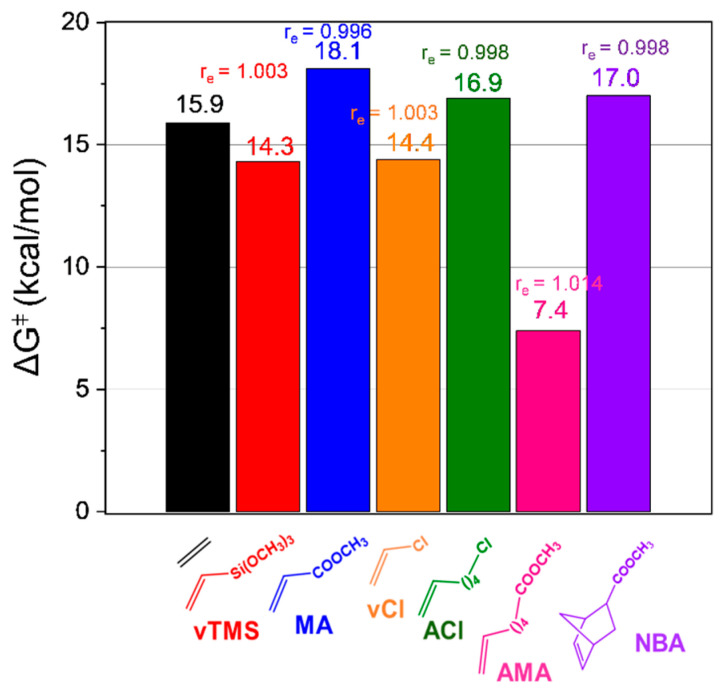
Activation free energy barriers for ethylene homopolymerization and copolymerization processes catalyzed by Ni-OH@SiO_2_. The comparison illustrates the kinetic feasibility of monomer insertion for ethylene and the studied polar comonomers, emphasizing the influence of monomer structure on catalytic efficiency (r_e_; discussed in the SI).

Although no experimental evidence currently confirms the insertion mode of all the polar monomers (PMs) studied here on the Ni-OH@SiO_2_ catalyst, our conformational sampling studies predict that the 2,1-insertion mode is the most stable pathway, both kinetically and thermodynamically (Table S1). This preference is consistent with the catalyst’s ability to stabilize transition states involving polar functional groups. Interestingly, while vCl exhibits thermodynamic favorability for product formation via the 1,2-regioselective pathway, this mode is also kinetically preferred, with a barrier approximately 2.0 kcal/mol lower than the 2,1-insertion (Table S1). The preference for 1,2-insertion with vCl is attributed to the formation of a highly stable chelate complex (β-Cl product; P_vCl_ in Figure 1), which leads to a strong interaction between the comonomer and the nickel center. Figure 4 further highlights the activation free energy differences between ethylene homopolymerization and vCl copolymerization. The lower activation free energy for vCl copolymerization (ΔG^ǂ^ = 14.4 kcal/mol) compared to ethylene homopolymerization (15.9 kcal/mol) indicates that the initial step of copolymerization is more kinetically favorable. This difference can be attributed to the strong electronic interactions between the Cl atom and the nickel. These findings emphasize the inherent trade-offs when incorporating polar monomers into the polymer chain. While polar monomers like vCl exhibit a lower activation barrier, their regioselective behavior and high chelation tendencies may compromise catalyst efficiency and polymer properties. This implies that while the incorporation of vCl is kinetically efficient, the chain growth process may be hindered by competing interactions, such as the stabilization of β-Cl chelate intermediates (P_vCl_ in Figure 1).

Figure 4 illustrates that the insertion of the AMA comonomer is both kinetically and thermodynamically more favorable than the homopolymerization pathway. The energy barrier for monomer insertion is notably low at 7.4 kcal/mol, and the process is highly exergonic by −17.6 kcal/mol. This computational prediction aligns well with experimental data for naphthoquinone-based Ni@SiO_2_ catalysts, which demonstrate high activity (up to 2.65 × 10^6^ g/mol h) during the copolymerization of ethylene with polar comonomers such as 5-hexene acetate [44] (AMA in this study). The production of high-molecular-weight polymers (Mn up to 630,000) by this catalyst suggests that similar performance can be anticipated for AMA copolymerization on the Ni-OH@SiO_2_ catalyst. This strong agreement between experimental and computational results underscores the robustness of the modeling approach in predicting catalyst efficiency and product outcomes [45,48].

**Figure 5:**
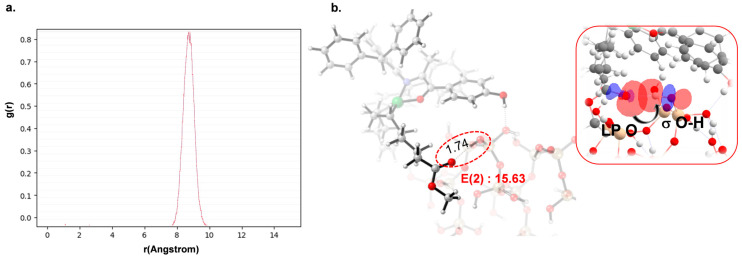
(**a**) Radial distribution function (RDF) between the ester carbonyl oxygen (O-ester) and the nearest surface hydrogen (H–SiO_2_) in the P_AMA_ structure showing a peak at ~8.5 Å indicative of a weak, non-persistent interaction. (**b**) Natural bond orbital (NBO) second-order perturbation energy analysis (E(2)) in the P_AMA_ structure reveals a significant orbital interaction between O-ester and H–SiO_2_ (O⋯H distances = 1.74 Å), consistent with transient adsorption. Atom color code: oxygen (red), nitrogen (blue), nickel (green), silicon (yellow), carbon (gray), hydrogen (white).

Nevertheless, the Ni-OH@SiO_2_ catalyst demonstrates markedly different behavior when a comonomer with a longer spacer, such as methyl 10-undecenoate, is employed. Experimental evidence indicates complete catalyst deactivation under these conditions. This deactivation is attributed to the strong interactions between the polar comonomer and the hydroxyl groups on the SiO_2_ surface, which likely block the active sites of the catalyst, preventing effective polymerization. To gain further insights into this phenomenon, we performed molecular dynamics simulations in the product structure of P_AMA_ to investigate the nature of the interaction between the AMA polar monomer and the SiO_2_ surface (Figure 5). The Forcite module in BIOVIA Materials Studio [49] was employed to optimize the system geometry and to conduct the simulations using the Universal Force Field (UFF). Dynamic equilibration was carried out in the NVT ensemble at 373 K for 1000 ps with a time step of 1 fs. This was followed by equilibration in the NPT ensemble for 5 ns. After these steps, a well-equilibrated structure was obtained. The radial distribution function (RDF) between the oxygen atom of the carbonyl group in the ester and the nearest hydrogen atom on the SiO_2_ surface was analyzed, as displayed in Figure 5. The RDF reveals a pronounced peak around 8.5 Å, indicating that this is the most visited distance between O-ester and H-SiO_2_ atoms during the simulation. However, the sharp decline in the distribution suggests that this interaction is transient and not persistently structured within the system. For strong interactions, such as hydrogen bonding, a characteristic distance of 1.5–2.0 Å is typically observed. Since no such peak is present, it indicates that there is no strong hydrogen bond formation between O-ester and H-SiO_2_. Instead, the interaction appears to be a weak spatial correlation, where these atoms have a high probability of being found at 8.5 Å without forming a stable bond.

Interestingly, NBO analysis in Figure 5 indicates a stabilization energy of 15.62 kcal/mol between the O-ester and H-SiO_2_ atoms, suggesting a significant orbital overlap. However, this interaction does not translate into persistent binding, as evidenced by the fluctuating distances observed in molecular dynamic simulation. The contrast between NBO findings and RDF results suggests that while electronic effects can temporarily stabilize the interaction, thermal fluctuations and dynamic effects prevent long-lasting adsorption. These findings support the hypothesis that the AMA comonomer does not strongly or persistently adhere to the SiO_2_ surface, allowing copolymerization to proceed efficiently without significant catalyst deactivation. Moreover, this insight prompts a reassessment of the proposed mechanism of catalyst deactivation observed experimentally with polar monomers containing longer spacers such as 10-undecenoate. Although a significant electronic interaction exists in the product structure, the lack of persistent adsorption under thermal conditions suggests that this factor alone is unlikely to be responsible for catalyst deactivation. Therefore, while our results do not support the strong interaction hypothesis as the cause of deactivation, they open avenues for further investigation into cooperative effects and surface structural reorganizations.

On the other hand, for TS_vTMS_, the computational results also indicate a kinetically and thermodynamically favorable insertion compared to ethylene homopolymerization. The activation barrier (∆G^ǂ^ = 14.3 kcal/mol) and reaction free energy (∆G° = −9.4 kcal/mol) for vTMS insertion are 1.6 and 2.3 kcal/mol lower, respectively, than those for ethylene homopolymerization. These findings suggest that vTMS is well suited for ethylene copolymerization, offering both enhanced catalytic activity and regioselectivity. This prediction aligns with experimental observations [9] for homogeneous nickel catalysts, further supporting the reliability of our computational results.

On the other hand, comonomers such as ACl, NBA, and MA exhibit moderate activation barriers in the range of 17.0–18.0 kcal/mol, which is higher than the barrier for ethylene homopolymerization. Although these comonomers are kinetically less favorable, the product formation process is more than that of ethylene stable (i.e., more exergonic as is shown in Figure 1), with ∆G° values of −7.9 kcal/mol for NBA, −7.6 kcal/mol for MA, and −5.0 kcal/mol for ACl, compared to −7.1 kcal/mol for ethylene homopolymerization. The lower exergonicity observed for ACl suggests reduced catalytic activity and the formation of lower-molecular-weight copolymers compared to ethylene homopolymerization. Conversely, the slightly more exergonic processes predicted for NBA and MA suggest that, thermodynamically, these comonomers could yield copolymers with molecular weights comparable to those of ethylene homopolymers. This aligns with experimental evidence [50] showing that, although nickel-based systems can copolymerize ethylene with acrylates like MA, the resulting polymers typically exhibit lower molecular weights and reduced catalytic activity—limitations that arise from strong electronic interactions and steric hindrance near the nickel coordination site.

In summary, our computational findings demonstrate that the catalytic activity and the resulting polymer properties are strongly influenced by the comonomer’s spacer length, polarity, and insertion energetics. Comonomers such as AMA and vTMS, with lower activation barriers and high thermodynamic stability, are predicted to achieve high incorporation rates and produce high-molecular-weight copolymers. Conversely, comonomers like ACl, NBA, and MA, while thermodynamically favorable, present higher kinetic barriers, potentially limiting their incorporation efficiency. While vCl, their regioselective behavior and high chelation tendencies may compromise catalyst efficiency. These insights are critical for optimizing copolymerization processes, enabling the tailored design of catalysts and monomers for the synthesis of advanced functional materials.

### 3.3. Nature of Activation Barriers for PM Insertion into Ni-OH@SiO_2_ Catalysts

**Figure 6:**
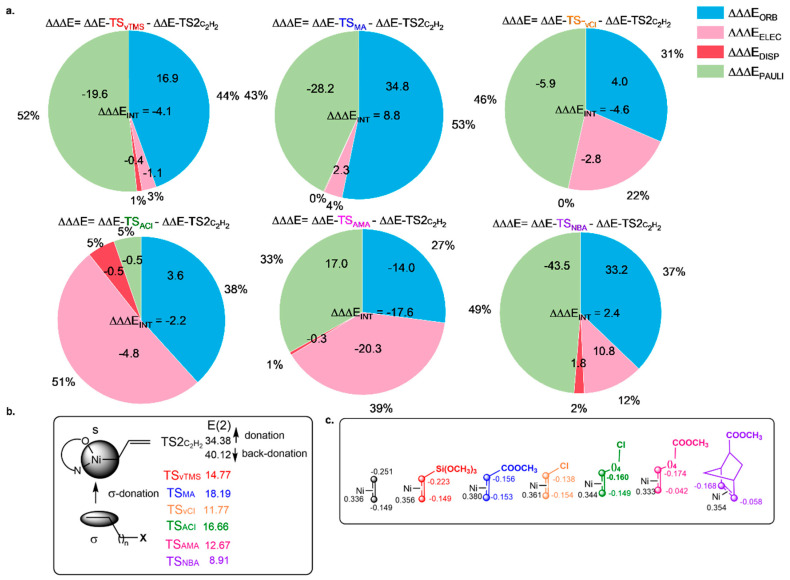
(**a**) Schematic representation of the interaction energy differences (∆∆E_INT_) in the energy barriers. The pie chart displays the interaction energy differences between the energy barriers (∆ΔE_INT_ = ∆E_TS_ − ∆E_I_) of the ethylene co-homopolymerization(∆∆ΔE_INT_ = Δ∆E_TS-PM_ − Δ∆E_TS2-C2H2_) in terms of orbital interaction (Δ∆∆E_ORB_), electrostatic (Δ∆∆E_ELEC_), dispersion (Δ∆∆E_DISP_) and Pauli repulsion (Δ∆∆E_PAULI_), contributions. Positive values favor ethylene homopolymerization, while negative values favor ethylene copolymerization. (**b**) Natural bond orbital (NBO) second-order perturbation energy analysis (E(2)), highlighting σ-donation between the Ni-OH@SiO_2_ catalyst and each monomer in the transition states. Energies are reported in kcal/mol. (**c**) CM5 atomic charges of the monomers in the transition state species.

To investigate the nature of the energy barriers and the selectivity of the copolymerization with the ethylene homopolymerization reaction, we studied the ALMO-EDA terms on the ground state (π-complex) and transition state structures (∆ΔE_INT_ = ∆E_TS_ − ∆E_I_) in order to predict the origin of the energy barriers. Figure 6a highlights the dominant quantitative contributions between the difference between the copolymerization and homopolymerization barriers (∆∆ΔE_INT_ = Δ∆E_TS-PM_ − Δ∆E_TS2-C2H2_). Positive ΔΔΔE_INT_ values indicate effects that promote the ethylene polymerization barrier; negative ΔΔΔE_INT_ values indicate effects that promote the ethylene copolymerization barrier.

In general, Figure 6a shows that each pie chart includes four different effects on selectivity; among these, it is observed that both types of electronic effects (∆ΔΔE_ORB_ and ∆ΔΔE_ELEC_) are the main stabilizing interactions for all the energy barriers. In contrast, the dispersion interactions are negligible (∆ΔΔE_DISP_). Orbital interactions (∆ΔΔE_ORB_) are the main effect that promotes the homopolymerization pathway (31–53%), except for the copolymerization with the AMA comonomer, which will be discussed later. Interestingly, in this context, NBO analyses (Figure 6b) consistently show that the dominant orbital interaction during the transition state corresponds to the σ(C-C) → LP*(Ni) charge transfer with a C–C vinyl bond of around 1.44 Å. This suggests a concerted insertion pathway, analogous in spirit to the Chalk–Harrod mechanism, where the vinyl σ-bond engages directly with a low-lying acceptor orbital on the metal center. The LP* orbital has a significant 4s character, given the geometry and electronic configuration of the Ni(II) center, and serves as a favorable site for charge donation in the absence of strong d–π* back-donation. The prominence of this σ-type interaction, even in sterically or electronically complex systems, indicates a compensatory mechanism: enhanced σ-donation can occur despite steric congestion as the system attempts to stabilize the transition state through orbital realignment. However, this compensation often comes at the cost of increased Pauli repulsion. This is evidenced in comonomers like MA, which exhibits one of the highest NBO stabilization energies (Figure 6b; E(2) = 18.19 kcal/mol) among the comonomers studied (Figure 6b). This strong interaction is consistent with the highly electrophilic character of the nickel center in the transition state (Figure 6c; +0.38 e), which enhances σ-donation from the vinyl group via a strong σ → 4s (Ni) interaction, increasing the Pauli repulsion (Figure 6a) and the activation barrier. These findings are consistent with previous mechanistic observations by Chen and Brookhart [9], who reported that polar monomers such as MA and vinyl halides tend to form stable chelates, hindering catalyst turnover. In addition, Zhang et al. [45] experimentally observed that MA shows limited incorporation into copolymers, even under Pd-SiO_2_ supported catalysis, due to its strong electron-withdrawing ester group directly conjugated to the vinyl system, which aligns with our theoretical results.

Similar to MA comonomer, NBA comonomer also exhibits a less stable insertion into the nickel center than the homopolymerization process, which is evidenced by positive ∆∆ΔE_INT_ values (Figure 6a). For both comonomers, the Pauli repulsion contribution is the dominant effect (∆∆ΔE_PAULI_: 43% for MA and 49% for NBA comonomers), which stems from unfavorable steric and electronic interactions near the nickel center. Interestingly, for NBA comonomer, a lower stabilization energy is observed (Figure 6b; E(2) = 8.91 kcal/mol), and the charge distribution across the vinyl carbons is highly asymmetric (Figure 6c; −0.058 e and −0.168 e), which disrupts the electronic balance and reduces the efficiency of orbital interactions with the nickel center. The combined effect of weak orbital stabilization and high Pauli repulsion explains the relatively poor reactivity of NBA in comparison with other polar comonomers. In both cases, MA and NBA, although their electronic profiles differ, the inability to offset destabilizing repulsion through favorable electrostatic or orbital contributions renders their insertion pathways less competitive compared to ethylene. Interestingly, although ethylene is a symmetric molecule in its free state, the vinyl group becomes significantly polarized in the transition state (Δe = 0.102 e). Despite this high polarization, ethylene exhibits the strongest σ → LP(Ni) interaction (Figure 6b; E(2) = 34.48 kcal/mol) due to its small size, ideal orbital orientation, and lack of steric perturbations. This contrasts with NBA, which also shows high polarization (Δe = 0.110 e) but much weaker orbital stabilization (E(2) = 8.91 kcal/mol), suggesting that the directionality and orbital alignment of the polarized vinyl system are more critical than the absolute magnitude of charge separation alone.

In contrast, ACl, vCl, vTMS, and AMA comonomers exhibit a stronger total interaction energy difference (Figure 6a; negative ∆∆ΔE_INT_ values), indicating that the transition state for these comonomer insertions is inherently more stable than that of ethylene. Particularly, although ACl comonomer has a slightly higher activation barrier (ΔG^ǂ^ = 16.9 kcal/mol) compared to ethylene homopolymerization (ΔG^ǂ^ = 15.9 kcal/mol), its interaction energy (ΔΔΔE_INT_ = −2.2 kcal/mol) suggests a more stable transition state. This apparent contradiction can be explained by the need for greater electronic reorganization before insertion (I_ACl_ species), which increases the initial energy cost. However, once the system reaches the transition state, favorable electrostatic interactions (∆∆ΔE_ELEC_: 51%) and reduced Pauli repulsion (∆∆ΔE_PAULI_: 5%) stabilize the insertion process, making ACl energetically more favorable overall. This interpretation is further supported by an independent gradient model based on Hirshfeld analysis (IGMH) [51] (Figure S2), which reveals significant van der Waals interactions (green isosurfaces) between the ACl monomer and the SiO_2_ support and clear electrostatic attraction (blue isosurfaces) between the vinyl group and the Ni center. These non-covalent interactions contribute to the stabilization of the transition state beyond direct orbital interactions, providing a favorable environment that reinforces electrostatic complementarity and minimizes steric repulsion. The involvement of the support highlights the importance of considering the full catalytic system when evaluating monomer–catalyst interactions. This is in contrast to NBA comonomer, which has a similar activation barrier (ΔG^ǂ^ = 17.0 kcal/mol) but lacks the stabilizing energy terms (Figure 6a), resulting in a less favorable insertion pathway.

Similar to ACl in structure, vCl exhibits a slightly lower activation barrier (ΔG^ǂ^ = 14.4 kcal/mol) and lower electrostatic stabilization (∆∆ΔE_ELEC_: 22%). The increased Pauli repulsion (∆∆ΔE_PAULI_: 46%) arises from the 1,2-insertion mode, which induces spatial proximity between the nickel center and the electron-withdrawing chlorine atom. This interaction perturbs the electronic environment, reducing the efficiency of ΔΔΔE_ORB_ interactions between the vinyl group and the Ni center (Figure 6b; E(2) = 11.77 kcal/mol) compared to ethylene. Moreover, as observed with the MA comonomer, the higher positive charge on the nickel center in the vCl transition state (+0.36 e) compared to ethylene (+0.34 e) may indicate a partial donation of electron density from the chlorine atom to the metal. This electron donation enhances the effective Lewis acidity of the nickel center, leading to a strong electrostatic attraction—evidenced by the blue isosurfaces—between the chlorine atom and the nickel (Figure S2). Altogether, this combination of steric proximity, electronic perturbation, and reduced orbital overlap contributes to a less stabilized transition state despite the modest activation barrier. These findings highlight the importance of designing monomers that prevent post-insertion destabilization, particularly by minimizing unfavorable interactions between polar substituents in FPMs and the metal center.

The insertion of vTMS presents a moderately favorable transition state, with a relative interaction energy of ΔΔΔE_INT_ = −4.1 kcal/mol. However, Figure 6a indicates that this stabilization does not arise from stabilizing contributions; ΔΔΔE_ELEC_ and ΔΔΔE_DISP_ dispersion terms are only marginally more favorable for vTMS than for ethylene, and ΔΔΔE_ORB_ is strongly favored for ethylene homopolymerization (Figure 6b). This apparent discrepancy can be rationalized by the pronounced stabilization of the π-complex in the vTMS system (Figure 3). Consequently, the overall interaction energy of the transition state appears more favorable, even though the classic stabilizing components are not strongly enhanced.

Lastly, among all the comonomers studied, AMA exhibits the most favorable insertion profile (Figure 6a). Its energy barrier is highly stabilized, with a ΔΔΔE_INT_ of −17.6 kcal/mol, and every stabilizing component (ΔΔΔE_ORB_, ΔΔΔE_DISP_, ΔΔΔE_ELEC_) is more favorable than in ethylene (Figure 6a). Figure 6b confirms that AMA has the strongest σ(C-C) → LP(Ni) interaction (E(2) = 24.88 kcal/mol) among the polar comonomers. This efficiency is supported by the pronounced polarization of the vinyl group (−0.174 e and −0.042 e), which creates a highly directional donor orbital that aligns well with the vacant orbital on Ni. The presence of an aliphatic spacer between the acetate group and the vinyl moiety minimizes electronic perturbation of the π-system and avoids steric congestion at the metal center. These features combine to produce a remarkably low activation barrier (~7.4 kcal/mol), consistent with experimental observations of high incorporation and molecular weight in AMA-based copolymers [44]. In contrast, MA places the electron-withdrawing ester group in direct conjugation with the vinyl system, which increases electron density near the nickel center and enhances Pauli repulsion. The superior performance of AMA over its parent monomer MA aligns with the design principle highlighted by Chen and Brookhart [9], who emphasize the importance of spatially separating the polar group from the reactive vinyl moiety. In AMA, the aliphatic spacer effectively insulates the π-system from electronic perturbations, minimizing Pauli repulsion and enhancing orbital overlap with the nickel center—features reflected in its exceptionally low barrier and high E(2) interaction energy. In addition, the lower charge on the nickel center in the AMA system (Figure 6c; +0.33 e) reflects reduced electrophilicity, which has been associated with enhanced copolymerization tolerance toward polar groups. This is in agreement with observations in supported Ni@SiO_2_ catalysts [44], where neutral nickel centers enabled higher molecular weights and better performance in ethylene copolymerization with AMA comonomer systems.

In summary, ALMO-EDA analysis in the activation barriers reveals structure–reactivity relationships governing the copolymerization behavior of polar comonomers with nickel catalysts. AMA, with minimal Pauli repulsion, enhanced electrostatic and orbital interactions, and optimal vinyl polarization, surpasses ethylene in favorability. The use of aliphatic spacers in AMA decouples the functional group from the reactive site, promoting efficient interaction with the metal center. These results highlight the importance of tuning both electronic and steric features to design comonomers that not only coordinate well with the catalyst but also promote smooth and favorable insertion pathways.

## 4. Conclusions

Via DFT calculations and ALMO-EDA analyses, we have unraveled the key factors that govern monomer incorporation and selectivity for the ethylene copolymerization with some fundamental and special polar monomers catalyzed by a silica-supported Ni–α-imine ketone catalyst (Ni-OH@SiO_2_). The reactivity trends reveal a strong structure–reactivity relationship driven by the electronic nature, spacer length, and steric profile of each comonomer.

Fundamental polar monomers (FPMs) such as MA and vCl exhibit strong electron-withdrawing effects directly conjugated to the vinyl group. These monomers lead to higher Pauli repulsion, elevated activation barriers (MA), lower incorporation efficiency (MA, vCl) and catalyst deactivation (vCl) due to stable chelation and regioselective insertion pathways. Meanwhile, special polar monomers (SPMs) such as NBA and ACl showed intermediate reactivity, with moderate activation barriers and thermodynamically stable insertion products. ACl benefitted from electrostatic stabilization and reduced Pauli repulsion, while NBA was limited by poor orbital interaction and higher Pauli repulsion.

AMA and vTMS exhibited the most favorable behavior. AMA showed the lowest activation barrier and strongest orbital interaction, aided by a flexible aliphatic spacer that minimized steric congestion and electronic perturbation. Although electronic interactions between AMA and the SiO_2_ support were observed, these do not appear to inhibit the copolymerization process, as they are transient and thermally labile. vTMS also demonstrated efficient incorporation, with a balance of π-complex stabilization and favorable electrostatics, despite modest Pauli repulsion.

These findings provide a valuable theoretical foundation for interpreting experimentally observed activity trends in nickel-based heterogeneous catalysts. By analyzing the electronic and energetic factors governing monomer insertion, the study offers mechanistic insight that complements reported performance data and supports the rational design of improved catalytic systems. This framework enables the development of structure–reactivity relationships and guides the tuning of monomer structures to optimize ethylene copolymerization with polar comonomers under industrially relevant conditions.

## Data Availability

All data are available in the Supporting Information document.

## Supplementary Materials

The following supporting information can be downloaded at: https://www.mdpi.com/article/10.3390/polym17091268/s1, Figure S1: Schematic representation of the interaction energy differences (∆∆E_INT_) in the contributions to the π-complexes; Figure S2: IGMH analysis for the transition states TS_vCl_ and TS_ACl_ into the Ni–OH@SiO_2_ catalyst; Table S1:Computed free energies for the coordination and insertion modes (1,2- and 2,1-) of polar monomers (PMs) into the Ni–OH@SiO_2_ catalyst.
