# Supplementary material for: Unraveling the Reactivity of SiO2-Supported Nickel Catalyst in Ethylene Copolymerization with Polar Monomers: A Theoretical Study
Source: Polymers (Basel). 2025 May 6;17(9):1268. doi: 10.3390/polym17091268 (PMC12073392; doi:10.3390/polym17091268)
Supplement: Supplementary file 1 [file polymers-17-01268-s001.zip › polymers-3595379-supplementary.pdf]

Supporting Information

Unraveling the Reactivity of SiO_2-Supported Nickel Catalyst in Ethylene Copolymerization with Polar Monomers: A Theoretical Study

Daniela E. Ortega [1,*] and Diego Cortés-Arriagada [2]

[1] Centro Integrativo de Biología y Química Aplicada (CIBQA), Universidad Bernardo O'Higgins,
General Gana 1702, Santiago 8370854, Chile
[2] Instituto Universitario de Investigación y Desarrollo Tecnológico (IDT), Universidad Tecnológica
Metropolitana, Ignacio Valdivieso 2409, San Joaquín, Santiago 8940577, Chile; dcortes@utem.cl
* Correspondence: daniela.ortega@ubo.cl

Table S1. Computed free energies (in kcal/mol) for the coordination and insertion modes (1,2- and 2,1-) of polar monomers (PMs) into the Ni–OH@SiO_2 catalyst. The table summarizes the Gibbs free energy barriers ($\Delta G^{\neq}$; $\Delta G°$), of 1,2- and 2,1-insertion modes. Lower values are indicated in red.

Coordination		Insertion manner							
		**$TS_{PM\text{-}1,2\text{-}ins.}$**	**$TS_{PM\text{-}2,1\text{-}ins.}$**	**$P_{PM\text{-}1,2\text{-}ins.}$**	**$P_{PM\text{-}2,1\text{-}ins.}$**	**$\Delta G^{\neq}_{\text{-}1,2\ ins.}$**	**$\Delta G^{\neq}_{\text{-}2,1\ ins.}$**	**$\Delta G°_{\text{-}1,2\ ins}$**	**$\Delta G°_{\text{-}2,1\ ins}$**
vTMS	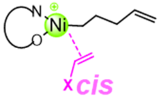	3.8	2.2	-19.3	-21.5	15.9	14.3	-7.2	-9.4
MA	I_{PM}	14.2	12.0	-12.3	-13.7	20.3	18.1	-6.2	-7.6
vCl		8.6	10.0	-12.8	-9.4	14.4	15.8	-7.0	-3.6
ACl		10.8	8.6	-9.1	-13.3	19.1	16.9	-0.8	-5.0
AMA		2.9	1.1	-20.5	-23.9	9.2	7.4	-14.2	-17.6
NBA		11.3	10.5	-13.1	-14.4	17.8	17.0	-6.8	-7.9

A detailed analysis of the interaction energy changes ($\Delta\Delta E_{INT} = \Delta EI_{PM} - \Delta E\text{-}I2_{C2H2}$) within the energy decomposition analysis ($\Delta\Delta E_{ORB}$, $\Delta\Delta E_{ELEC}$, $\Delta\Delta E_{DISP}$, $\Delta\Delta E_{PAULI}$) framework between the polar monomers and ethylene in the π-complexes is presented in Fig. S1. Positive $\Delta\Delta E_{INT}$ values indicate effects that promote ethylene polymerization; negative $\Delta\Delta E_{INT}$ values indicate effects that promote ethylene copolymerization π-complex. All $\Delta\Delta E_{INT}$ values are negative, confirming the stability of the interaction between the catalyst and the comonomers. Particularly, it is observed that each I_{PM} π-complex is more stable than the $I2_{C2H2}$ complex, confirming their remarkable thermodynamic stability (Fig. 1 in the main

manuscript). Dispersion interactions ($\Delta\Delta E_{DISP}$) favor special polar monomers (ACl, AMA, and NBA) in ~ 25% since they also have greater contact with the SiO_2 surface. Notably, Pauli repulsion ($\Delta\Delta E_{PAULI}$) emerges as the primary destabilizing factor in the π-complexes, accounting for 32–45% of the total interaction energy (Fig. S1), following the trend: MA (45%) > NBA (43%) > vTMS (42%) > ACl (38%) > vCl (34%) > AMA (32%). This observation aligns with the expected behavior, as ethylene, being a smaller and more symmetric molecule, can engage more effectively with the nickel center. Specifically, ethylene exhibits strong σ donation from its π-electron pair to the nickel's vacant 4s orbital and π back-donation from the nickel's d-orbitals to the ethylene's π* orbital. These interactions are characterized by higher donor-acceptor stabilization energies [E(2), Fig. 3b in the main manuscript], enabling ethylene to achieve more efficient orbital overlap and less steric disruption, thereby minimizing Pauli repulsion.

In contrast, polar monomers, which possess bulkier (vTMS), electronic strain (NBA), and more electronegative substituents (vCl, MA), experience greater steric hindrance and electronic polarization at the metal center (above 40%). This results in higher Pauli repulsion, as their substituents disrupt the optimal overlap of orbitals with the nickel center. Additionally, the electron-withdrawing nature of polar groups in these monomers can reduce the efficiency of π back-donation (vCl), further contributing to their less favorable interaction compared to ethylene (see Fig. 3b in the main manuscript). Thus, while polar monomers stabilize the π-complexes through electrostatic and orbital contributions ($\Delta\Delta E_{ORB} + \Delta\Delta E_{ELEC} \approx 40\%$). Their incorporation inherently imposes a greater degree of steric and electronic strain on the system.

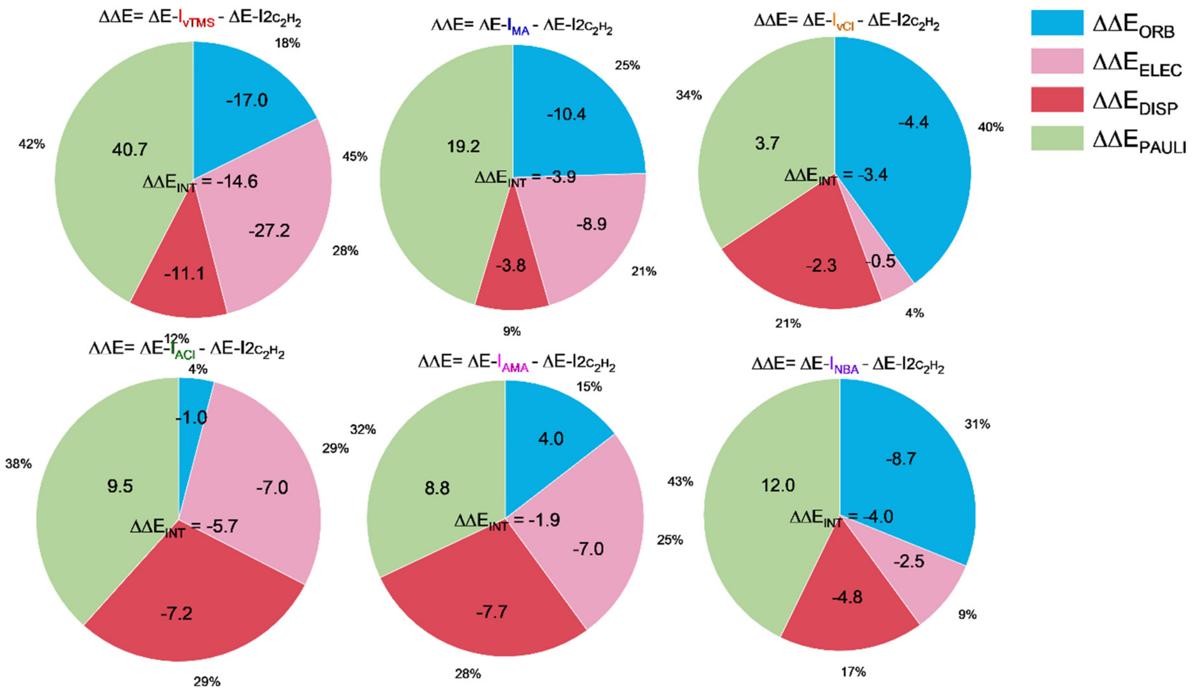

Figure S1. Schematic representation of the interaction energy differences ($\Delta\Delta E_{INT}$) in the contributions to the π-complexes. The pie chart displays the interaction energy differences between the I_{PM} and I_{C2H2} π-complexes ($\Delta\Delta E_{INT}$ = ΔE-I_{PM} - ΔE-I_{C2H2}) in terms of orbital interaction ($\Delta\Delta E_{ORB}$), electrostatic ($\Delta\Delta E_{ELEC}$), dispersion ($\Delta\Delta E_{DISP}$) and Pauli repulsion ($\Delta\Delta E_{PAULI}$), contributions. The energies are in kcal/mol.

On the other hand, to quantify the likelihood of polar comonomer incorporation into the growing polymer chain, we analyzed the Gibbs free energy differences for competing insertion transition states using the reactivity ratio (Eq. S1):

$$r_e = \frac{k_{ee}}{k_{ec}} = e^{\frac{\Delta G^{\ddagger}_{ec} - \Delta G^{\ddagger}_{ee}}{RT}} \qquad \text{(S1)}$$

Where r_e is the reactivity ratio of ethylene, k_{ee}, and k_{ec} are the rates of monomer insertions of ethylene (e) and comonomer (c) given the previous insertion of monomer ethylene, respectively. $\Delta G^{\ddagger}_{ec}$ and $\Delta G^{\ddagger}_{ee}$ are the Gibbs free energy barriers of the insertion of the comonomer after ethylene and the insertion of ethylene, respectively. R and T are the gas constant and the temperature, respectively. This approach, based on the Curtin-Hammett principle, evaluates the relative rates of comonomer (c) and ethylene (e) insertion following the insertion of a prior ethylene unit. The reactivity ratios reveal distinct trends in catalytic

efficiency and selectivity. For example, AMA displays a significantly lower activation barrier, resulting in a preferential incorporation into the copolymer (r_e = 1.014). Similarly, vTMS and vCl exhibit reactivity ratios near unity ($r_e \approx$ 1.002), indicating comparable insertion efficiency to ethylene. In contrast, comonomers like MA, NBA, and ACl exhibit reactivity ratios below unity (r_e = 0.996–0.998), reflecting reduced reactivity and less efficient incorporation into the copolymer chain. This difference can be rationalized by specific electronic and steric factors. Special monomers present lower insertion barriers resulting from favorable electronic interactions and reduced steric hindrance in their transition states, whereas fundamental monomers show less favorable interactions, increasing their r_e values and limiting their effective incorporation into the polymer chain. Thus, the analysis via the r_e parameter not only confirms previously observed trends from individual insertion barriers but also provides an additional, robust, and readily interpretable metric, enhancing the overall understanding of catalyst selectivity toward different monomers.

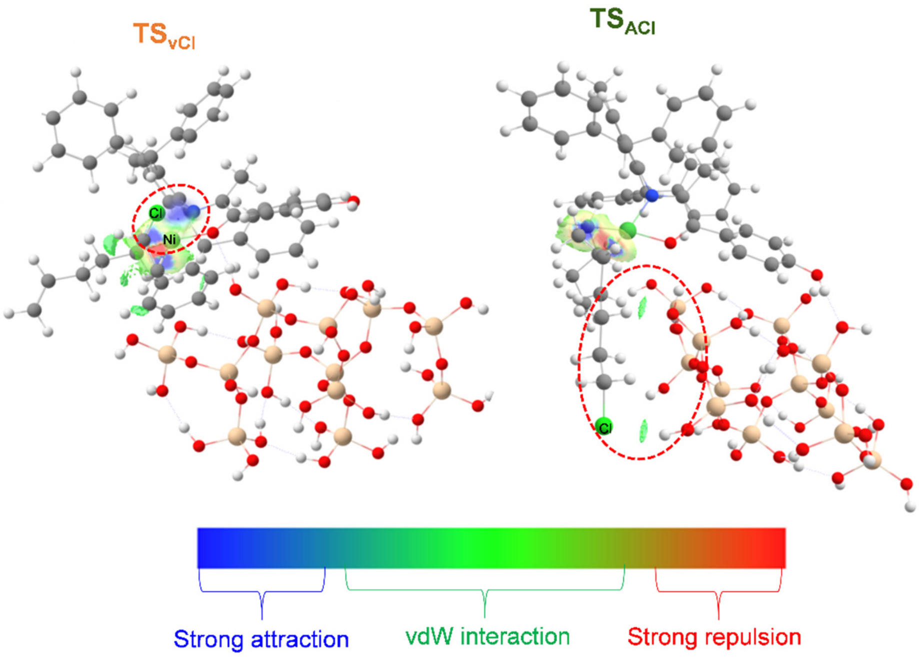

Figure S2. IGMH analysis for the transition states TS_{vCl} and TS_{ACl} into the Ni–OH@SiO_2 catalyst. The isosurfaces represent non-covalent interactions, colored by the sign(λ_2)ρ function: blue indicates strong attraction (e.g., electrostatics), green corresponds to van der Waals (vdW) interactions, and red highlights strong repulsive interactions (Pauli repulsion).

Cartesian coordinates of the optimized structures

C_2H_2

C	0.000000000000	0.665166000000	0.000000000000
H	0.925220000000	1.234562000000	0.000000000000
H	-0.925182000000	1.234603000000	0.000000000000
C	0.000000000000	-0.665166000000	0.000000000000
H	-0.925220000000	-1.234562000000	0.000000000000
H	0.925182000000	-1.234603000000	0.000000000000

Ni-OH@SiO_2

O	6.064504000000	1.831581000000	-0.444378000000
Si	4.544905000000	1.950589000000	0.112259000000
O	4.286151000000	0.697508000000	1.104936000000
Si	3.302052000000	-0.467216000000	1.645964000000
O	3.228134000000	-1.768730000000	0.672324000000
O	3.401433000000	1.846977000000	-1.072474000000
O	4.296014000000	3.365137000000	0.892975000000
Si	4.690194000000	4.342507000000	2.112369000000
O	3.357121000000	4.875413000000	2.858346000000
Si	2.270961000000	6.086204000000	3.034359000000
O	0.808340000000	5.358870000000	2.824351000000
O	1.833192000000	0.118062000000	1.943494000000
Si	0.882922000000	1.276358000000	2.567979000000
O	-0.623011000000	0.662344000000	2.696244000000
O	4.049857000000	-0.954464000000	3.058550000000
O	5.653204000000	5.553884000000	1.654736000000
Si	5.933016000000	6.760398000000	0.578624000000
O	5.142435000000	8.047003000000	1.293946000000
O	5.382156000000	6.448144000000	-0.909227000000
O	7.549789000000	6.975998000000	0.451033000000
O	5.569702000000	3.490917000000	3.224850000000
Si	5.396696000000	2.417667000000	4.465591000000
O	4.058551000000	1.491032000000	4.303320000000
O	0.831132000000	2.618054000000	1.607054000000
Si	0.176259000000	3.201486000000	0.214695000000
O	-1.352988000000	3.741337000000	0.454141000000
Si	-2.242939000000	4.945086000000	1.136682000000
O	-3.832580000000	4.576496000000	1.051608000000
O	0.153324000000	2.049685000000	-0.939594000000
O	1.119031000000	4.482337000000	-0.161337000000
Si	1.419112000000	5.619290000000	-1.319057000000
O	2.596464000000	6.596862000000	-0.715514000000
O	2.100380000000	4.871967000000	-2.616477000000
O	0.051520000000	6.380082000000	-1.767312000000
O	1.398861000000	1.809381000000	4.020906000000
O	6.774929000000	1.542421000000	4.466350000000
Si	7.816016000000	0.673687000000	3.543663000000
O	6.893486000000	-0.359493000000	2.616090000000

O	8.665618000000	1.591874000000	2.512854000000
Si	9.058836000000	3.091803000000	2.009850000000
O	10.660620000000	3.286259000000	1.801437000000
O	8.297207000000	3.221544000000	0.541293000000
O	8.516628000000	4.233710000000	3.059847000000
O	8.746985000000	-0.139323000000	4.597830000000
O	5.179440000000	3.207477000000	5.882811000000
O	-1.831033000000	6.330627000000	0.323013000000
O	-1.949210000000	5.074885000000	2.737829000000
O	2.490729000000	7.266238000000	1.910459000000
O	2.439230000000	6.806411000000	4.490743000000
H	6.800532000000	2.375117000000	-0.112513000000
H	3.379425000000	2.576148000000	-1.704448000000
H	5.019042000000	-1.011934000000	2.940173000000
H	-1.038176000000	0.677803000000	3.569899000000
H	2.356408000000	1.785149000000	4.202621000000
H	-4.184369000000	4.265581000000	0.214752000000
H	-1.066521000000	5.420183000000	2.959985000000
H	2.744789000000	-1.717371000000	-0.155303000000
H	0.917329000000	4.431349000000	2.551297000000
H	2.448862000000	6.232376000000	5.265792000000
H	5.864491000000	3.822974000000	6.154171000000
H	4.153695000000	0.568098000000	3.994052000000
H	11.187648000000	3.642978000000	2.519264000000
H	8.502451000000	4.004834000000	0.019534000000
H	7.555495000000	4.262384000000	3.165291000000
H	5.246599000000	8.900484000000	0.862893000000
H	8.052655000000	7.010242000000	1.268711000000
H	6.887025000000	-0.162590000000	1.671817000000
H	9.433833000000	-0.720684000000	4.265046000000
H	-0.667167000000	6.434493000000	-1.115097000000
H	1.663513000000	4.983022000000	-3.463695000000
H	1.028171000000	1.721478000000	-1.184206000000
H	-2.092005000000	7.160752000000	0.734118000000
H	3.355553000000	7.719191000000	1.928642000000
H	4.413108000000	6.475401000000	-1.030705000000
H	2.447456000000	6.911147000000	0.198003000000
Ni	-3.140827000000	2.237500000000	4.984342000000
N	-2.473035000000	2.907207000000	6.652954000000
C	-4.473884000000	3.593305000000	4.464378000000
H	-3.843276000000	4.285252000000	3.903169000000
H	-5.111176000000	4.035789000000	5.225758000000
C	-4.812563000000	2.346757000000	3.906001000000
C	-3.810682000000	1.683949000000	3.186209000000
H	-3.917170000000	0.631168000000	2.943416000000
H	-3.115126000000	2.246338000000	2.563205000000
C	-0.982038000000	1.170453000000	6.255966000000
C	-1.452996000000	2.302991000000	7.157563000000
H	-5.660366000000	1.786100000000	4.297519000000
O	-1.736189000000	0.952546000000	5.271720000000
C	-0.845044000000	2.625906000000	8.480071000000
H	-0.649708000000	1.708464000000	9.040994000000

H	0.090098000000	3.176652000000	8.342535000000
H	-1.517271000000	3.257621000000	9.057692000000
C	-3.111487000000	3.981435000000	7.353527000000
C	-2.707894000000	5.292408000000	7.089983000000
C	-4.186836000000	3.682583000000	8.197215000000
C	-3.319408000000	6.313979000000	7.814735000000
C	-4.776419000000	4.737098000000	8.889900000000
C	-4.332203000000	6.053188000000	8.739009000000
H	-3.025019000000	7.342154000000	7.622317000000
H	-5.621719000000	4.529734000000	9.540516000000
C	-4.954104000000	7.169397000000	9.537899000000
H	-4.447229000000	7.282225000000	10.502343000000
H	-4.880202000000	8.124418000000	9.011805000000
H	-6.009488000000	6.971224000000	9.741726000000
C	-4.695052000000	2.243411000000	8.282932000000
H	-4.609653000000	1.836722000000	7.265355000000
C	-1.669587000000	5.544861000000	6.001298000000
H	-1.842571000000	4.776488000000	5.236396000000
C	-6.179903000000	2.170236000000	8.624386000000
C	-6.669295000000	1.645814000000	9.818999000000
C	-7.093864000000	2.656541000000	7.681742000000
C	-8.042222000000	1.618515000000	10.071419000000
H	-5.985903000000	1.245164000000	10.560038000000
C	-8.460596000000	2.630512000000	7.928755000000
H	-6.724928000000	3.069417000000	6.745345000000
C	-8.940448000000	2.111282000000	9.131748000000
H	-8.405527000000	1.205118000000	11.006839000000
H	-9.152603000000	3.012455000000	7.184861000000
H	-10.007098000000	2.087486000000	9.329199000000
C	-3.802195000000	1.374086000000	9.153551000000
C	-3.325070000000	0.157072000000	8.660853000000
C	-3.421454000000	1.773125000000	10.437016000000
C	-2.476367000000	-0.641176000000	9.425557000000
H	-3.620185000000	-0.168234000000	7.665485000000
C	-2.584189000000	0.973407000000	11.210617000000
H	-3.775674000000	2.722493000000	10.830124000000
C	-2.102968000000	-0.233607000000	10.704653000000
H	-2.112745000000	-1.582337000000	9.025052000000
H	-2.302538000000	1.295308000000	12.208219000000
H	-1.446421000000	-0.854364000000	11.305576000000
C	-1.860807000000	6.861993000000	5.251669000000
C	-3.134399000000	7.195825000000	4.774048000000
C	-0.784271000000	7.671614000000	4.893187000000
C	-3.325096000000	8.304456000000	3.959442000000
H	-3.986180000000	6.573023000000	5.030706000000
C	-0.969798000000	8.781671000000	4.067144000000
H	0.218362000000	7.436379000000	5.230054000000
C	-2.238278000000	9.102215000000	3.595849000000
H	-4.322220000000	8.544461000000	3.603141000000
H	-0.113232000000	9.391154000000	3.796629000000
H	-2.384756000000	9.971530000000	2.962036000000
C	-0.256960000000	5.308496000000	6.510739000000

C	0.565591000000	4.375411000000	5.876949000000
C	0.236799000000	5.992756000000	7.625209000000
C	1.858779000000	4.132017000000	6.339674000000
H	0.207040000000	3.825898000000	5.011601000000
C	1.527393000000	5.756618000000	8.091178000000
H	-0.394423000000	6.718052000000	8.130812000000
C	2.344472000000	4.823316000000	7.448660000000
H	2.488910000000	3.404773000000	5.840534000000
H	1.897107000000	6.300198000000	8.954679000000
H	3.350996000000	4.630595000000	7.805594000000
C	0.217355000000	0.390829000000	6.404520000000
C	0.356124000000	-0.742168000000	5.569471000000
C	1.342687000000	0.817241000000	7.142245000000
C	1.569601000000	-1.369269000000	5.415180000000
H	-0.504615000000	-1.108451000000	5.021912000000
C	2.563160000000	0.195541000000	6.993714000000
H	1.296085000000	1.687760000000	7.778587000000
C	2.694789000000	-0.871208000000	6.094556000000
H	1.668351000000	-2.224008000000	4.753415000000
H	3.444164000000	0.559323000000	7.509178000000
O	3.917586000000	-1.380476000000	5.915045000000
H	3.943957000000	-1.902693000000	5.101028000000

I_{C2H2}

O	6.116407000000	1.516725000000	-0.168616000000
Si	4.544907000000	1.950591000000	0.112257000000
O	4.056135000000	0.921425000000	1.273838000000
Si	3.302046000000	-0.467222000000	1.645963000000
O	3.349770000000	-1.657469000000	0.514502000000
O	3.487119000000	1.871398000000	-1.176154000000
O	4.408710000000	3.499796000000	0.725525000000
Si	4.690193000000	4.342506000000	2.112391000000
O	3.317813000000	4.785652000000	2.904110000000
Si	2.270960000000	6.086201000000	3.034355000000
O	0.781825000000	5.350231000000	2.836464000000
O	1.753738000000	-0.064817000000	2.076108000000
Si	0.882943000000	1.276349000000	2.568002000000
O	-0.706386000000	0.781012000000	2.632345000000
O	4.165256000000	-1.006513000000	3.003352000000
O	5.622017000000	5.655917000000	1.794652000000
Si	5.933018000000	6.760399000000	0.578621000000
O	5.143887000000	8.114829000000	1.233635000000
O	5.341356000000	6.370880000000	-0.896605000000
O	7.578537000000	6.885406000000	0.437731000000
O	5.613923000000	3.409755000000	3.140172000000
Si	5.396698000000	2.417665000000	4.465577000000
O	4.052783000000	1.444876000000	4.370382000000
O	0.997029000000	2.578088000000	1.529767000000
Si	0.176204000000	3.201549000000	0.214678000000
O	-1.381837000000	3.564229000000	0.675887000000
Si	-2.242906000000	4.945052000000	1.136670000000
O	-3.848499000000	4.550131000000	1.261829000000

O	0.160969000000	2.136850000000	-1.044873000000
O	0.978946000000	4.619330000000	-0.056040000000
Si	1.419118000000	5.619275000000	-1.319052000000
O	2.590943000000	6.651720000000	-0.745128000000
O	2.213833000000	4.653028000000	-2.439623000000
O	0.093342000000	6.335856000000	-1.978224000000
O	1.356705000000	1.811276000000	4.053660000000
O	6.822234000000	1.566735000000	4.542256000000
Si	7.816015000000	0.673698000000	3.543663000000
O	6.790083000000	-0.145176000000	2.468154000000
O	8.854702000000	1.580404000000	2.646387000000
Si	9.058833000000	3.091797000000	2.009851000000
O	10.644062000000	3.415174000000	1.704330000000
O	8.261116000000	3.060555000000	0.517556000000
O	8.423637000000	4.284014000000	2.977493000000
O	8.596413000000	-0.376084000000	4.537217000000
O	5.146049000000	3.311265000000	5.836100000000
O	-1.958210000000	6.043174000000	-0.121295000000
O	-1.851969000000	5.532977000000	2.615593000000
O	2.562042000000	7.230394000000	1.857755000000
O	2.386298000000	6.850142000000	4.489752000000
H	6.865062000000	2.163212000000	-0.051154000000
H	3.385817000000	2.719383000000	-1.665039000000
H	5.152144000000	-0.909880000000	2.837499000000
H	-1.332744000000	1.375041000000	2.177861000000
H	2.325189000000	1.739932000000	4.264252000000
H	-4.253200000000	4.219103000000	0.441354000000
H	-0.875853000000	5.642692000000	2.783403000000
H	2.749832000000	-1.582166000000	-0.247971000000
H	0.925267000000	4.422535000000	2.529914000000
H	2.278094000000	6.273822000000	5.276515000000
H	5.844262000000	3.956632000000	6.043668000000
H	4.181937000000	0.529766000000	4.003902000000
H	11.194983000000	3.684906000000	2.459020000000
H	8.410032000000	3.871714000000	-0.007769000000
H	7.448996000000	4.208860000000	3.086352000000
H	5.192256000000	8.919109000000	0.683830000000
H	8.044962000000	6.736855000000	1.281689000000
H	6.714048000000	0.261619000000	1.571659000000
H	9.296278000000	-0.914754000000	4.129079000000
H	-0.716554000000	6.222759000000	-1.417224000000
H	1.845092000000	4.694531000000	-3.339644000000
H	1.067985000000	1.859649000000	-1.293904000000
H	-2.322588000000	6.929423000000	0.064243000000
H	3.456372000000	7.674908000000	1.861166000000
H	4.351611000000	6.453039000000	-0.986730000000
H	2.447634000000	6.947167000000	0.199547000000
Ni	-3.528250000000	1.896810000000	6.313634000000
N	-2.361044000000	2.509614000000	7.847324000000
C	-2.398509000000	2.830198000000	4.528743000000
H	-1.643638000000	2.030415000000	4.489900000000
H	-2.051751000000	3.870412000000	4.438774000000

C	-3.747599000000	2.557989000000	4.329244000000
C	-4.253799000000	1.241481000000	4.574602000000
H	-5.336665000000	1.061984000000	4.477232000000
H	-3.610971000000	0.364454000000	4.386725000000
C	-1.106196000000	0.671173000000	7.069604000000
C	-1.238507000000	1.840747000000	8.017224000000
H	-4.440598000000	3.397712000000	4.149563000000
O	-2.121529000000	0.463061000000	6.341192000000
C	-0.168432000000	2.253990000000	8.976305000000
H	-0.010122000000	1.487875000000	9.760361000000
H	0.785285000000	2.389377000000	8.428337000000
H	-0.426614000000	3.204193000000	9.473695000000
C	-2.695262000000	3.617512000000	8.706869000000
C	-2.449694000000	4.947341000000	8.267993000000
C	-3.332765000000	3.379180000000	9.962268000000
C	-2.875460000000	6.017460000000	9.076950000000
C	-3.723419000000	4.490536000000	10.730052000000
C	-3.514842000000	5.818082000000	10.311329000000
H	-2.709771000000	7.044078000000	8.714418000000
H	-4.234522000000	4.303644000000	11.687471000000
C	-3.983166000000	6.975990000000	11.155712000000
H	-3.650444000000	6.871391000000	12.208482000000
H	-3.609866000000	7.943971000000	10.770392000000
H	-5.092311000000	7.028376000000	11.177132000000
C	-3.570465000000	1.964759000000	10.509591000000
H	-3.707388000000	1.303149000000	9.629654000000
C	-1.666197000000	5.213067000000	6.983479000000
H	-1.832435000000	4.326802000000	6.339589000000
C	-4.861907000000	1.854065000000	11.340832000000
C	-4.863339000000	1.418079000000	12.680243000000
C	-6.101530000000	2.177228000000	10.744290000000
C	-6.064714000000	1.311818000000	13.402737000000
H	-3.915893000000	1.152394000000	13.170753000000
C	-7.300374000000	2.068652000000	11.461879000000
H	-6.129972000000	2.528937000000	9.702142000000
C	-7.287137000000	1.635030000000	12.798257000000
H	-6.039147000000	0.969615000000	14.448645000000
H	-8.253197000000	2.324447000000	10.973391000000
H	-8.227384000000	1.549260000000	13.363670000000
C	-2.351210000000	1.400450000000	11.242262000000
C	-2.012177000000	0.041614000000	11.071883000000
C	-1.565315000000	2.184463000000	12.110969000000
C	-0.908276000000	-0.516838000000	11.735954000000
H	-2.625212000000	-0.590226000000	10.408452000000
C	-0.462636000000	1.628710000000	12.779804000000
H	-1.811296000000	3.247151000000	12.258708000000
C	-0.126128000000	0.278005000000	12.590878000000
H	-0.660829000000	-1.579349000000	11.588206000000
H	0.139872000000	2.257082000000	13.453606000000
H	0.739070000000	-0.156352000000	13.114429000000
C	-2.187835000000	6.409452000000	6.177106000000
C	-3.571098000000	6.502598000000	5.904449000000

C	-1.332967000000	7.384750000000	5.627675000000
C	-4.084872000000	7.543763000000	5.120236000000
H	-4.257206000000	5.748697000000	6.322895000000
C	-1.847444000000	8.430832000000	4.842388000000
H	-0.247744000000	7.327167000000	5.795569000000
C	-3.222947000000	8.517221000000	4.588040000000
H	-5.166817000000	7.597296000000	4.923873000000
H	-1.159167000000	9.184107000000	4.428867000000
H	-3.625742000000	9.340156000000	3.977983000000
C	-0.158468000000	5.254169000000	7.248596000000
C	0.717735000000	4.477231000000	6.462636000000
C	0.393996000000	6.062123000000	8.265623000000
C	2.105970000000	4.495372000000	6.695024000000
H	0.325382000000	3.833071000000	5.660167000000
C	1.778319000000	6.094182000000	8.493574000000
H	-0.268964000000	6.674847000000	8.894633000000
C	2.639796000000	5.306410000000	7.711996000000
H	2.780632000000	3.863435000000	6.098827000000
H	2.185188000000	6.733508000000	9.291933000000
H	3.724731000000	5.310776000000	7.896467000000
C	0.109630000000	-0.109627000000	6.845119000000
C	0.218599000000	-0.749859000000	5.575888000000
C	1.215189000000	-0.232745000000	7.737494000000
C	1.396729000000	-1.358138000000	5.167942000000
H	-0.632218000000	-0.695874000000	4.883346000000
C	2.391989000000	-0.859440000000	7.342807000000
H	1.155886000000	0.147940000000	8.763173000000
C	2.527465000000	-1.361878000000	6.022884000000
H	1.470397000000	-1.792363000000	4.160187000000
H	3.251302000000	-0.936316000000	8.024189000000
O	3.726649000000	-1.813043000000	5.636087000000
H	3.769151000000	-1.864786000000	4.646535000000
C	-5.325323000000	2.556125000000	6.949620000000
H	-5.968232000000	2.865806000000	6.108415000000
H	-5.139303000000	3.339704000000	7.704213000000
C	-5.182335000000	1.193060000000	7.272290000000
H	-4.897086000000	0.875845000000	8.286337000000
H	-5.689849000000	0.414459000000	6.681085000000

TS_{C2H2}

O	6.069502000000	1.819430000000	-0.470663000000
Si	4.544941000000	1.950584000000	0.112432000000
O	4.341218000000	0.734235000000	1.206098000000
Si	3.301796000000	-0.467023000000	1.645939000000
O	3.312235000000	-1.774941000000	0.649724000000
O	3.340574000000	1.764025000000	-1.039004000000
O	4.262242000000	3.432153000000	0.819560000000
Si	4.690157000000	4.342486000000	2.112305000000
O	3.365618000000	4.831736000000	2.954493000000
Si	2.270973000000	6.086183000000	3.034358000000
O	0.802375000000	5.336548000000	2.754809000000
O	1.791154000000	0.124319000000	1.807613000000

Si	0.883272000000	1.276202000000	2.567997000000
O	-0.592279000000	0.558047000000	2.696439000000
O	4.003210000000	-0.970447000000	3.111284000000
O	5.658599000000	5.603120000000	1.742249000000
Si	5.933023000000	6.760394000000	0.578624000000
O	5.158381000000	8.090026000000	1.297492000000
O	5.326408000000	6.412560000000	-0.896077000000
O	7.579310000000	6.889312000000	0.421620000000
O	5.622980000000	3.416655000000	3.144913000000
Si	5.396700000000	2.417644000000	4.465578000000
O	4.061159000000	1.447833000000	4.337073000000
O	0.765914000000	2.708895000000	1.701045000000
Si	0.176113000000	3.201526000000	0.214593000000
O	-1.398915000000	3.742929000000	0.324263000000
Si	-2.242933000000	4.945078000000	1.136666000000
O	-3.879835000000	4.664322000000	1.039256000000
O	0.252515000000	1.965056000000	-0.874647000000
O	1.162819000000	4.482972000000	-0.121460000000
Si	1.419134000000	5.619277000000	-1.319027000000
O	2.624766000000	6.616606000000	-0.733126000000
O	2.093166000000	4.831549000000	-2.621421000000
O	0.018309000000	6.372314000000	-1.742601000000
O	1.472723000000	1.753471000000	4.025521000000
O	6.833393000000	1.586827000000	4.537548000000
Si	7.816018000000	0.673745000000	3.543653000000
O	6.765531000000	-0.289559000000	2.623894000000
O	8.736232000000	1.538776000000	2.490002000000
Si	9.058835000000	3.091766000000	2.009859000000
O	10.660258000000	3.342440000000	1.731700000000
O	8.225556000000	3.232311000000	0.545603000000
O	8.477852000000	4.213156000000	3.090176000000
O	8.714640000000	-0.244657000000	4.568683000000
O	5.103666000000	3.291551000000	5.840199000000
O	-1.782065000000	6.392408000000	0.415629000000
O	-1.894386000000	4.900369000000	2.749981000000
O	2.565396000000	7.243789000000	1.868413000000
O	2.364775000000	6.854059000000	4.492978000000
H	6.821868000000	2.370587000000	-0.125954000000
H	3.271454000000	2.526562000000	-1.651770000000
H	4.998311000000	-0.982341000000	3.001216000000
H	-1.259315000000	0.935685000000	3.311225000000
H	2.457588000000	1.739708000000	4.179358000000
H	-4.248941000000	4.708365000000	0.139610000000
H	-0.961918000000	5.207989000000	2.918392000000
H	2.789427000000	-1.707739000000	-0.168375000000
H	0.957552000000	4.394318000000	2.472553000000
H	2.305152000000	6.262228000000	5.272877000000
H	5.777605000000	3.959463000000	6.056184000000
H	4.151737000000	0.510566000000	4.008659000000
H	11.215555000000	3.542136000000	2.504776000000
H	8.303507000000	4.117761000000	0.136516000000
H	7.497887000000	4.161968000000	3.167956000000

H	5.193671000000	8.907251000000	0.765830000000
H	8.050998000000	6.827678000000	1.272882000000
H	6.626051000000	0.041532000000	1.712216000000
H	9.407496000000	-0.787892000000	4.154795000000
H	-0.656648000000	6.456033000000	-1.023185000000
H	1.624799000000	4.953692000000	-3.464872000000
H	1.181426000000	1.661748000000	-0.989338000000
H	-1.884803000000	7.172177000000	1.003757000000
H	3.467416000000	7.672091000000	1.880157000000
H	4.329684000000	6.470895000000	-0.973751000000
H	2.479280000000	6.907400000000	0.211684000000
Ni	-3.776951000000	2.533520000000	4.681489000000
N	-2.967766000000	3.201070000000	6.252994000000
C	-3.112746000000	0.511269000000	1.572353000000
H	-2.932208000000	-0.453584000000	2.070627000000
H	-2.575522000000	0.690060000000	0.628880000000
C	-3.943549000000	1.441318000000	2.083817000000
C	-4.750926000000	1.271140000000	3.323262000000
H	-5.792644000000	0.965751000000	3.116072000000
H	-4.357578000000	0.500052000000	4.023697000000
C	-1.581795000000	1.350892000000	5.947796000000
C	-2.006368000000	2.482999000000	6.814509000000
H	-4.084896000000	2.384714000000	1.527365000000
O	-2.329361000000	1.176566000000	4.914238000000
C	-1.459659000000	2.777103000000	8.173641000000
H	-1.312844000000	1.841410000000	8.745070000000
H	-0.490515000000	3.311132000000	8.097721000000
H	-2.146859000000	3.433474000000	8.733198000000
C	-3.515387000000	4.309999000000	6.980525000000
C	-2.971083000000	5.597573000000	6.781360000000
C	-4.646106000000	4.098751000000	7.804361000000
C	-3.509355000000	6.664409000000	7.520429000000
C	-5.147962000000	5.194511000000	8.525113000000
C	-4.575679000000	6.479342000000	8.422112000000
H	-3.108330000000	7.676971000000	7.356315000000
H	-6.036789000000	5.046147000000	9.157621000000
C	-5.108285000000	7.626755000000	9.240808000000
H	-4.910561000000	8.602301000000	8.755914000000
H	-6.199013000000	7.535405000000	9.410321000000
H	-4.625987000000	7.654492000000	10.241507000000
C	-5.296408000000	2.714663000000	7.821243000000
H	-5.184581000000	2.336335000000	6.775682000000
C	-1.874660000000	5.764428000000	5.739395000000
H	-2.040701000000	4.940050000000	5.013087000000
C	-6.798049000000	2.756622000000	8.096914000000
C	-7.400857000000	2.049140000000	9.152440000000
C	-7.624648000000	3.510323000000	7.234056000000
C	-8.791875000000	2.100402000000	9.347121000000
H	-6.780190000000	1.444672000000	9.829102000000
C	-9.010955000000	3.561759000000	7.425424000000
H	-7.169841000000	4.078773000000	6.407138000000
C	-9.601115000000	2.855566000000	8.487555000000

H	-9.243758000000	1.540133000000	10.179521000000
H	-9.636248000000	4.156572000000	6.742438000000
H	-10.689869000000	2.894170000000	8.641124000000
C	-4.519332000000	1.732636000000	8.687133000000
C	-4.093323000000	0.503442000000	8.147483000000
C	-4.192376000000	2.034319000000	10.023787000000
C	-3.346030000000	-0.401634000000	8.917444000000
H	-4.342649000000	0.262542000000	7.101340000000
C	-3.456375000000	1.127252000000	10.801226000000
H	-4.510784000000	2.996018000000	10.455304000000
C	-3.024839000000	-0.090583000000	10.248485000000
H	-3.013702000000	-1.353361000000	8.476063000000
H	-3.211999000000	1.374890000000	11.845286000000
H	-2.441477000000	-0.798022000000	10.856584000000
C	-1.994383000000	7.007103000000	4.855782000000
C	-3.255082000000	7.381776000000	4.341527000000
C	-0.850117000000	7.651553000000	4.350869000000
C	-3.361877000000	8.329576000000	3.315427000000
H	-4.164888000000	6.903286000000	4.731975000000
C	-0.950208000000	8.592487000000	3.312442000000
H	0.144783000000	7.392555000000	4.736183000000
C	-2.205487000000	8.927084000000	2.777821000000
H	-4.354686000000	8.600039000000	2.924577000000
H	-0.032856000000	9.056589000000	2.918413000000
H	-2.289132000000	9.674966000000	1.973738000000
C	-0.483948000000	5.516707000000	6.311130000000
C	0.323663000000	4.499902000000	5.763961000000
C	0.026591000000	6.282826000000	7.378679000000
C	1.616805000000	4.258885000000	6.258191000000
H	-0.050676000000	3.885663000000	4.932785000000
C	1.317949000000	6.048984000000	7.876126000000
H	-0.593371000000	7.077036000000	7.821627000000
C	2.120423000000	5.037170000000	7.315301000000
H	2.231938000000	3.467081000000	5.810543000000
H	1.703383000000	6.661822000000	8.704827000000
H	3.136253000000	4.847534000000	7.693138000000
C	-0.406784000000	0.525427000000	6.136762000000
C	-0.253711000000	-0.624579000000	5.305547000000
C	0.697652000000	0.894968000000	6.962065000000
C	0.964767000000	-1.277074000000	5.196412000000
H	-1.109604000000	-0.967134000000	4.711002000000
C	1.912418000000	0.234203000000	6.873833000000
H	0.638637000000	1.768636000000	7.616774000000
C	2.084856000000	-0.808027000000	5.929907000000
H	1.078252000000	-2.136228000000	4.517948000000
H	2.786428000000	0.568878000000	7.449540000000
O	3.318948000000	-1.300224000000	5.765413000000
H	3.414101000000	-1.681288000000	4.858907000000
C	-4.876311000000	4.053001000000	4.334437000000
H	-4.288867000000	4.709616000000	3.666356000000
H	-5.217128000000	4.516011000000	5.274338000000
C	-5.650699000000	3.000051000000	3.745561000000

H	-6.518394000000	2.634159000000	4.321243000000
H	-5.844136000000	3.086974000000	2.663612000000

P_{C2H2}

O	6.121476000000	1.446511000000	-0.045475000000
Si	4.544926000000	1.950570000000	0.112422000000
O	3.962584000000	0.963101000000	1.257063000000
Si	3.301798000000	-0.466998000000	1.645929000000
O	3.360492000000	-1.651842000000	0.509578000000
O	3.561266000000	1.915675000000	-1.228769000000
O	4.462115000000	3.505148000000	0.714776000000
Si	4.690162000000	4.342472000000	2.112336000000
O	3.281767000000	4.757024000000	2.860162000000
Si	2.270969000000	6.086182000000	3.034350000000
O	0.757413000000	5.398620000000	2.823674000000
O	1.752580000000	-0.089858000000	2.104489000000
Si	0.883291000000	1.276209000000	2.567999000000
O	-0.714239000000	0.874034000000	2.680410000000
O	4.242282000000	-0.968609000000	2.964527000000
O	5.590091000000	5.681386000000	1.812328000000
Si	5.933029000000	6.760396000000	0.578619000000
O	5.156843000000	8.130730000000	1.210911000000
O	5.337744000000	6.358953000000	-0.891679000000
O	7.583502000000	6.831015000000	0.459703000000
O	5.620553000000	3.444656000000	3.167847000000
Si	5.396696000000	2.417644000000	4.465577000000
O	4.068235000000	1.442902000000	4.300253000000
O	0.993192000000	2.535595000000	1.491760000000
Si	0.176087000000	3.201536000000	0.214588000000
O	-1.389145000000	3.625639000000	0.567331000000
Si	-2.242924000000	4.945067000000	1.136660000000
O	-3.869518000000	4.600585000000	1.076833000000
O	0.178046000000	2.177209000000	-1.082840000000
O	1.027739000000	4.604882000000	-0.045129000000
Si	1.419139000000	5.619266000000	-1.319016000000
O	2.593969000000	6.652448000000	-0.742807000000
O	2.213733000000	4.698780000000	-2.472780000000
O	0.065053000000	6.315799000000	-1.941920000000
O	1.429150000000	1.868309000000	4.011406000000
O	6.825168000000	1.570546000000	4.546804000000
Si	7.816020000000	0.673760000000	3.543648000000
O	6.779107000000	-0.076821000000	2.429622000000
O	8.882654000000	1.594433000000	2.691654000000
Si	9.058834000000	3.091756000000	2.009862000000
O	10.637351000000	3.429347000000	1.692629000000
O	8.252749000000	3.023563000000	0.522983000000
O	8.401003000000	4.300100000000	2.942348000000
O	8.556279000000	-0.425301000000	4.510941000000
O	5.103925000000	3.265306000000	5.856452000000
O	-1.827867000000	6.221487000000	0.112817000000
O	-1.947309000000	5.266856000000	2.722397000000
O	2.567467000000	7.249533000000	1.879689000000

O	2.468919000000	6.791174000000	4.513488000000
H	6.871989000000	2.102153000000	-0.008734000000
H	3.426903000000	2.789723000000	-1.660170000000
H	5.221298000000	-0.815572000000	2.760952000000
H	-1.012117000000	0.648215000000	3.595171000000
H	2.402649000000	1.768458000000	4.186549000000
H	-4.202679000000	4.400868000000	0.184160000000
H	-1.001117000000	5.509377000000	2.908049000000
H	2.714399000000	-1.599732000000	-0.216458000000
H	0.903333000000	4.475335000000	2.507726000000
H	2.395194000000	6.164350000000	5.264438000000
H	5.743660000000	3.971640000000	6.053733000000
H	4.213413000000	0.537328000000	3.909108000000
H	11.196301000000	3.680456000000	2.447776000000
H	8.344994000000	3.853136000000	0.012972000000
H	7.430946000000	4.197477000000	3.076741000000
H	5.182151000000	8.915292000000	0.632221000000
H	8.022968000000	6.613514000000	1.304352000000
H	6.678771000000	0.372729000000	1.548515000000
H	9.274150000000	-0.940324000000	4.103494000000
H	-0.692772000000	6.309141000000	-1.303621000000
H	1.734679000000	4.615673000000	-3.316023000000
H	1.087408000000	1.886850000000	-1.302161000000
H	-2.061178000000	7.098645000000	0.471457000000
H	3.470156000000	7.675460000000	1.873646000000
H	4.347514000000	6.446937000000	-0.979863000000
H	2.453264000000	6.934667000000	0.204657000000
Ni	-2.966241000000	2.274657000000	4.948537000000
N	-2.349333000000	2.811278000000	6.602134000000
C	-5.812273000000	-0.885941000000	3.386489000000
H	-5.836228000000	-1.034673000000	4.479775000000
H	-5.787620000000	-1.789349000000	2.758191000000
C	-5.830882000000	0.345994000000	2.849773000000
C	-5.839710000000	1.620012000000	3.646238000000
H	-6.711239000000	2.250802000000	3.362914000000
H	-5.953365000000	1.389169000000	4.726812000000
C	-0.847677000000	1.058588000000	6.232371000000
C	-1.316498000000	2.163069000000	7.123370000000
H	-5.810034000000	0.456544000000	1.749731000000
O	-1.616468000000	0.843365000000	5.224530000000
C	-0.743991000000	2.486370000000	8.461509000000
H	-0.565572000000	1.561242000000	9.042530000000
H	0.215901000000	3.031409000000	8.346490000000
H	-1.428046000000	3.140357000000	9.028207000000
C	-3.025710000000	3.833263000000	7.343764000000
C	-2.661316000000	5.184311000000	7.158843000000
C	-4.110942000000	3.457619000000	8.170553000000
C	-3.308661000000	6.151119000000	7.946984000000
C	-4.735943000000	4.460370000000	8.928116000000
C	-4.323100000000	5.807122000000	8.861796000000
H	-3.038670000000	7.209783000000	7.812066000000
H	-5.592304000000	4.184799000000	9.562744000000

C	-4.971817000000	6.854190000000	9.729702000000
H	-4.481364000000	6.900322000000	10.725781000000
H	-4.895141000000	7.863265000000	9.280598000000
H	-6.042102000000	6.630706000000	9.907106000000
C	-4.586712000000	2.004428000000	8.143444000000
H	-4.456760000000	1.687307000000	7.080998000000
C	-1.623516000000	5.520921000000	6.093359000000
H	-1.768056000000	4.758696000000	5.293329000000
C	-6.075438000000	1.856435000000	8.451297000000
C	-6.562965000000	1.104305000000	9.535001000000
C	-7.006073000000	2.485068000000	7.594963000000
C	-7.945241000000	0.990354000000	9.762704000000
H	-5.858109000000	0.594444000000	10.207166000000
C	-8.383605000000	2.371408000000	7.818690000000
H	-6.639956000000	3.083456000000	6.745614000000
C	-8.859214000000	1.622118000000	8.908459000000
H	-8.307207000000	0.397402000000	10.616165000000
H	-9.091865000000	2.868919000000	7.138961000000
H	-9.940921000000	1.530199000000	9.087204000000
C	-3.681803000000	1.088425000000	8.955383000000
C	-3.164660000000	-0.085772000000	8.372943000000
C	-3.332409000000	1.395011000000	10.285048000000
C	-2.306079000000	-0.929609000000	9.094966000000
H	-3.431637000000	-0.330755000000	7.332201000000
C	-2.486388000000	0.546229000000	11.015395000000
H	-3.721178000000	2.315277000000	10.748061000000
C	-1.964268000000	-0.614880000000	10.420216000000
H	-1.903523000000	-1.837357000000	8.620586000000
H	-2.226606000000	0.796110000000	12.055227000000
H	-1.294589000000	-1.275667000000	10.990732000000
C	-1.841409000000	6.859422000000	5.388525000000
C	-3.139353000000	7.232613000000	4.977691000000
C	-0.757691000000	7.665799000000	4.994159000000
C	-3.345378000000	8.374275000000	4.192903000000
H	-4.000589000000	6.611379000000	5.265076000000
C	-0.960613000000	8.805206000000	4.198133000000
H	0.267409000000	7.394227000000	5.277627000000
C	-2.254297000000	9.165258000000	3.793671000000
H	-4.366806000000	8.646477000000	3.885845000000
H	-0.092824000000	9.411059000000	3.895442000000
H	-2.415524000000	10.063463000000	3.178130000000
C	-0.207141000000	5.303134000000	6.607416000000
C	0.649265000000	4.405898000000	5.941897000000
C	0.270150000000	5.975055000000	7.750875000000
C	1.959346000000	4.187296000000	6.401239000000
H	0.300905000000	3.860339000000	5.053542000000
C	1.579023000000	5.764753000000	8.211491000000
H	-0.392864000000	6.673213000000	8.284381000000
C	2.429752000000	4.870280000000	7.536480000000
H	2.617576000000	3.482647000000	5.877235000000
H	1.937942000000	6.301964000000	9.102322000000
H	3.456624000000	4.693628000000	7.889732000000

C	0.366189000000	0.283439000000	6.355548000000
C	0.536097000000	-0.795588000000	5.437486000000
C	1.484784000000	0.655946000000	7.156491000000
C	1.772753000000	-1.380497000000	5.233612000000
H	-0.326947000000	-1.132050000000	4.847124000000
C	2.725732000000	0.062536000000	6.969767000000
H	1.409132000000	1.477560000000	7.875517000000
C	2.901687000000	-0.912304000000	5.956620000000
H	1.896605000000	-2.182512000000	4.491232000000
H	3.607920000000	0.402121000000	7.529822000000
O	4.140341000000	-1.342529000000	5.694379000000
H	4.163736000000	-1.691977000000	4.768169000000
C	-4.289183000000	3.527329000000	4.457534000000
H	-3.816080000000	4.454859000000	4.080778000000
H	-5.058413000000	3.708378000000	5.232108000000
C	-4.579435000000	2.474668000000	3.434907000000
H	-4.473562000000	2.887019000000	2.405793000000
H	-3.679692000000	1.704400000000	3.356889000000

I2$_{C2H2}$

O	6.079670000000	1.765422000000	-0.433615000000
Si	4.544920000000	1.950561000000	0.112409000000
O	4.300332000000	0.767113000000	1.225164000000
Si	3.301783000000	-0.466980000000	1.645930000000
O	3.326647000000	-1.755107000000	0.625823000000
O	3.352856000000	1.786785000000	-1.053972000000
O	4.293536000000	3.446634000000	0.800484000000
Si	4.690165000000	4.342464000000	2.112349000000
O	3.353206000000	4.818845000000	2.943922000000
Si	2.270968000000	6.086181000000	3.034350000000
O	0.797864000000	5.339181000000	2.774153000000
O	1.778327000000	0.083220000000	1.850190000000
Si	0.883317000000	1.276225000000	2.567992000000
O	-0.635444000000	0.640571000000	2.709688000000
O	4.043698000000	-0.970912000000	3.094285000000
O	5.648029000000	5.617593000000	1.756841000000
Si	5.933030000000	6.760399000000	0.578616000000
O	5.155262000000	8.097422000000	1.279262000000
O	5.331928000000	6.396789000000	-0.894940000000
O	7.580239000000	6.881654000000	0.427327000000
O	5.623575000000	3.422609000000	3.150467000000
Si	5.396694000000	2.417645000000	4.465577000000
O	4.060483000000	1.449999000000	4.324701000000
O	0.816492000000	2.685398000000	1.669306000000
Si	0.176062000000	3.201565000000	0.214578000000
O	-1.404500000000	3.713267000000	0.361758000000
Si	-2.242926000000	4.945039000000	1.136660000000
O	-3.876051000000	4.654247000000	1.056924000000
O	0.273226000000	2.005323000000	-0.915546000000
O	1.126888000000	4.520424000000	-0.094661000000
Si	1.419160000000	5.619240000000	-1.318996000000
O	2.625974000000	6.618882000000	-0.740640000000

O	2.115374000000	4.797651000000	-2.591109000000
O	0.029540000000	6.368343000000	-1.785232000000
O	1.460633000000	1.761060000000	4.031316000000
O	6.830375000000	1.581928000000	4.538234000000
Si	7.816021000000	0.673766000000	3.543645000000
O	6.765526000000	-0.262175000000	2.593883000000
O	8.757810000000	1.541692000000	2.512559000000
Si	9.058833000000	3.091752000000	2.009864000000
O	10.656318000000	3.352966000000	1.720515000000
O	8.220260000000	3.212296000000	0.546856000000
O	8.472778000000	4.219862000000	3.080207000000
O	8.691773000000	-0.269240000000	4.565410000000
O	5.102507000000	3.280454000000	5.847404000000
O	-1.778246000000	6.370285000000	0.373250000000
O	-1.883912000000	4.957566000000	2.748900000000
O	2.563716000000	7.240137000000	1.865657000000
O	2.381501000000	6.853186000000	4.492407000000
H	6.826999000000	2.339690000000	-0.116257000000
H	3.288017000000	2.562302000000	-1.651909000000
H	5.037897000000	-0.947324000000	2.960925000000
H	-1.094116000000	0.813919000000	3.568295000000
H	2.444798000000	1.739118000000	4.186714000000
H	-4.240752000000	4.513418000000	0.165640000000
H	-0.951327000000	5.268426000000	2.910400000000
H	2.749948000000	-1.708509000000	-0.156747000000
H	0.955177000000	4.404610000000	2.473241000000
H	2.328982000000	6.258827000000	5.270448000000
H	5.774755000000	3.948870000000	6.067434000000
H	4.163292000000	0.520590000000	3.976719000000
H	11.217930000000	3.552651000000	2.488982000000
H	8.297591000000	4.093116000000	0.127583000000
H	7.492899000000	4.169019000000	3.159018000000
H	5.186255000000	8.907478000000	0.736490000000
H	8.048614000000	6.815421000000	1.280123000000
H	6.662588000000	0.071085000000	1.677604000000
H	9.391693000000	-0.805744000000	4.154552000000
H	-0.660770000000	6.440321000000	-1.079242000000
H	1.613150000000	4.833707000000	-3.423367000000
H	1.205865000000	1.713098000000	-1.029175000000
H	-1.895657000000	7.163742000000	0.937375000000
H	3.464032000000	7.671812000000	1.875380000000
H	4.336271000000	6.460117000000	-0.977775000000
H	2.480839000000	6.907776000000	0.204409000000
Ni	-3.474708000000	2.452182000000	4.686576000000
N	-2.796517000000	3.111783000000	6.418706000000
C	-7.678779000000	5.842975000000	1.167192000000
H	-6.767892000000	6.262576000000	0.706259000000
H	-8.594783000000	5.837032000000	0.556513000000
C	-7.661746000000	5.370945000000	2.426009000000
C	-6.442547000000	5.332600000000	3.301954000000
H	-5.614859000000	5.887082000000	2.813569000000
H	-6.651657000000	5.838416000000	4.272124000000

C	-1.304104000000	1.338186000000	6.087753000000
C	-1.787590000000	2.457738000000	6.957447000000
H	-8.590727000000	4.951432000000	2.855821000000
O	-2.006957000000	1.168804000000	5.028913000000
C	-1.222255000000	2.775206000000	8.301878000000
H	-1.069737000000	1.845635000000	8.883154000000
H	-0.249800000000	3.298321000000	8.199860000000
H	-1.899350000000	3.443476000000	8.859299000000
C	-3.377300000000	4.206159000000	7.130184000000
C	-2.889589000000	5.512573000000	6.901194000000
C	-4.506297000000	3.964783000000	7.947163000000
C	-3.471636000000	6.568358000000	7.621794000000
C	-5.055154000000	5.050643000000	8.647446000000
C	-4.531606000000	6.354521000000	8.525435000000
H	-3.115795000000	7.594167000000	7.437779000000
H	-5.941793000000	4.877992000000	9.276728000000
C	-5.112024000000	7.493763000000	9.322450000000
H	-4.629870000000	7.561775000000	10.321315000000
H	-4.957165000000	8.467205000000	8.818156000000
H	-6.197466000000	7.358441000000	9.496080000000
C	-5.099502000000	2.555620000000	7.971128000000
H	-5.001321000000	2.188811000000	6.921539000000
C	-1.814117000000	5.708360000000	5.840441000000
H	-1.968846000000	4.881459000000	5.113155000000
C	-6.594374000000	2.549235000000	8.279646000000
C	-7.144684000000	1.937810000000	9.419933000000
C	-7.465005000000	3.182674000000	7.365634000000
C	-8.531379000000	1.968901000000	9.648164000000
H	-6.488028000000	1.423427000000	10.136164000000
C	-8.846331000000	3.214353000000	7.591139000000
H	-7.046265000000	3.662536000000	6.466741000000
C	-9.385515000000	2.607231000000	8.738541000000
H	-8.944518000000	1.484224000000	10.545603000000
H	-9.508418000000	3.712489000000	6.866650000000
H	-10.470762000000	2.628891000000	8.918274000000
C	-4.263422000000	1.604964000000	8.816629000000
C	-3.803391000000	0.391757000000	8.266616000000
C	-3.908655000000	1.922793000000	10.142565000000
C	-2.999831000000	-0.481305000000	9.016832000000
H	-4.071966000000	0.138722000000	7.228129000000
C	-3.117348000000	1.046330000000	10.901009000000
H	-4.248247000000	2.874476000000	10.580200000000
C	-2.654544000000	-0.155432000000	10.338407000000
H	-2.643092000000	-1.420600000000	8.567795000000
H	-2.854045000000	1.305852000000	11.937585000000
H	-2.028350000000	-0.838753000000	10.931307000000
C	-1.978395000000	6.951117000000	4.964560000000
C	-3.257192000000	7.294540000000	4.473737000000
C	-0.858949000000	7.626404000000	4.444516000000
C	-3.405501000000	8.244168000000	3.454824000000
H	-4.147223000000	6.786099000000	4.870556000000
C	-1.001871000000	8.571086000000	3.414441000000

H	0.149094000000	7.389037000000	4.809490000000
C	-2.274463000000	8.875482000000	2.903334000000
H	-4.412161000000	8.487275000000	3.081172000000
H	-0.104263000000	9.063251000000	3.009013000000
H	-2.390976000000	9.625823000000	2.105519000000
C	-0.410089000000	5.484991000000	6.387526000000
C	0.413398000000	4.498519000000	5.809084000000
C	0.092249000000	6.235090000000	7.469877000000
C	1.713295000000	4.268477000000	6.291077000000
H	0.046966000000	3.899839000000	4.962983000000
C	1.390490000000	6.012522000000	7.954668000000
H	-0.541305000000	7.004818000000	7.936308000000
C	2.207420000000	5.028677000000	7.365783000000
H	2.342370000000	3.499247000000	5.823778000000
H	1.769521000000	6.611646000000	8.796252000000
H	3.227583000000	4.846958000000	7.735626000000
C	-0.130188000000	0.515210000000	6.286200000000
C	0.017118000000	-0.625632000000	5.439349000000
C	0.984599000000	0.887953000000	7.094267000000
C	1.236948000000	-1.264070000000	5.296532000000
H	-0.845707000000	-0.969912000000	4.854041000000
C	2.207046000000	0.245235000000	6.965111000000
H	0.927587000000	1.752878000000	7.761724000000
C	2.369298000000	-0.787495000000	6.008493000000
H	1.344585000000	-2.117625000000	4.610236000000
H	3.090610000000	0.586485000000	7.522060000000
O	3.600403000000	-1.266869000000	5.802467000000
H	3.656318000000	-1.653338000000	4.893592000000
C	-4.678955000000	3.910473000000	4.388907000000
H	-3.889701000000	4.516007000000	3.880144000000
H	-4.861657000000	4.323202000000	5.401527000000
C	-5.959165000000	3.887429000000	3.584783000000
H	-5.828240000000	3.393484000000	2.602079000000
H	-6.758891000000	3.332212000000	4.122933000000
C	-3.760801000000	1.697623000000	2.809529000000
H	-2.793879000000	1.259760000000	2.509183000000
H	-4.134674000000	2.501268000000	2.155849000000
C	-4.584627000000	1.058924000000	3.738407000000
H	-5.653139000000	1.308129000000	3.832816000000
H	-4.272837000000	0.100121000000	4.187098000000

$TS2_{C2H2}$

O	6.121278000000	1.489147000000	-0.111518000000
Si	4.544922000000	1.950570000000	0.112412000000
O	4.017028000000	0.942840000000	1.269703000000
Si	3.301793000000	-0.466997000000	1.645930000000
O	3.365291000000	-1.660140000000	0.517762000000
O	3.508301000000	1.875396000000	-1.190393000000
O	4.432002000000	3.499766000000	0.723256000000
Si	4.690161000000	4.342471000000	2.112337000000
O	3.312757000000	4.774790000000	2.903356000000
Si	2.270970000000	6.086180000000	3.034351000000

O	0.783102000000	5.369874000000	2.775309000000
O	1.739181000000	-0.063122000000	2.026509000000
Si	0.883301000000	1.276231000000	2.567992000000
O	-0.715644000000	0.845297000000	2.738254000000
O	4.222846000000	-0.973844000000	2.975992000000
O	5.605393000000	5.667377000000	1.801630000000
Si	5.933029000000	6.760396000000	0.578619000000
O	5.165942000000	8.127217000000	1.228756000000
O	5.335807000000	6.367730000000	-0.891443000000
O	7.583937000000	6.836237000000	0.449235000000
O	5.624944000000	3.436920000000	3.162215000000
Si	5.396696000000	2.417645000000	4.465578000000
O	4.078167000000	1.433969000000	4.288021000000
O	0.902766000000	2.609925000000	1.590193000000
Si	0.176068000000	3.201561000000	0.214588000000
O	-1.394472000000	3.677115000000	0.415240000000
Si	-2.242928000000	4.945041000000	1.136661000000
O	-3.856260000000	4.698722000000	0.918775000000
O	0.236306000000	2.058126000000	-0.979827000000
O	1.119168000000	4.531679000000	-0.082032000000
Si	1.419161000000	5.619246000000	-1.319003000000
O	2.618588000000	6.628775000000	-0.742854000000
O	2.146129000000	4.777069000000	-2.565520000000
O	0.030982000000	6.345366000000	-1.817283000000
O	1.454164000000	1.775405000000	4.032267000000
O	6.829599000000	1.573671000000	4.548594000000
Si	7.816020000000	0.673760000000	3.543648000000
O	6.772033000000	-0.088904000000	2.441342000000
O	8.872637000000	1.583398000000	2.667690000000
Si	9.058834000000	3.091756000000	2.009862000000
O	10.638740000000	3.417841000000	1.688417000000
O	8.238536000000	3.056695000000	0.529646000000
O	8.415698000000	4.286228000000	2.969078000000
O	8.565468000000	-0.418506000000	4.512066000000
O	5.089413000000	3.258013000000	5.857512000000
O	-1.706922000000	6.351306000000	0.377989000000
O	-1.902432000000	4.974959000000	2.751460000000
O	2.590029000000	7.239290000000	1.875177000000
O	2.379470000000	6.820266000000	4.507500000000
H	6.867632000000	2.144974000000	-0.024095000000
H	3.412683000000	2.716811000000	-1.687872000000
H	5.199493000000	-0.818005000000	2.764901000000
H	-0.939883000000	0.448152000000	3.606616000000
H	2.433629000000	1.702289000000	4.190870000000
H	-4.055608000000	3.809310000000	0.539632000000
H	-0.983539000000	5.298269000000	2.938871000000
H	2.728487000000	-1.604323000000	-0.216271000000
H	0.942912000000	4.444864000000	2.463973000000
H	2.277786000000	6.211309000000	5.269630000000
H	5.729277000000	3.960673000000	6.067431000000
H	4.226062000000	0.528282000000	3.896842000000
H	11.203063000000	3.657573000000	2.443267000000

H	8.315061000000	3.902678000000	0.043885000000
H	7.442546000000	4.195126000000	3.089344000000
H	5.183777000000	8.913637000000	0.652164000000
H	8.030483000000	6.657942000000	1.298823000000
H	6.686854000000	0.349320000000	1.556396000000
H	9.289031000000	-0.925516000000	4.104543000000
H	-0.664088000000	6.415935000000	-1.112968000000
H	1.638728000000	4.766776000000	-3.395643000000
H	1.162899000000	1.802918000000	-1.175041000000
H	-1.816424000000	7.152853000000	0.925350000000
H	3.489685000000	7.670958000000	1.876870000000
H	4.342233000000	6.445875000000	-0.977142000000
H	2.480750000000	6.913512000000	0.204232000000
Ni	-3.307547000000	1.200990000000	5.466234000000
N	-2.406292000000	2.414437000000	6.744302000000
C	-4.444979000000	1.848134000000	-0.256379000000
H	-5.527580000000	1.992673000000	-0.414806000000
H	-3.797499000000	1.849899000000	-1.146915000000
C	-3.927455000000	1.653229000000	0.972931000000
C	-4.711516000000	1.600101000000	2.253899000000
H	-5.776724000000	1.859474000000	2.070280000000
H	-4.699029000000	0.538751000000	2.589113000000
C	-0.777931000000	0.795806000000	6.399730000000
C	-1.275045000000	1.927923000000	7.227807000000
H	-2.837429000000	1.505045000000	1.081977000000
O	-1.624291000000	0.368617000000	5.524081000000
C	-0.614133000000	2.423924000000	8.472267000000
H	-0.329628000000	1.576186000000	9.125331000000
H	0.295854000000	3.008107000000	8.229731000000
H	-1.294062000000	3.090533000000	9.029081000000
C	-3.085663000000	3.460768000000	7.441303000000
C	-2.839506000000	4.814373000000	7.107790000000
C	-4.054647000000	3.108662000000	8.414755000000
C	-3.505146000000	5.808489000000	7.847048000000
C	-4.693742000000	4.137053000000	9.123430000000
C	-4.414003000000	5.495835000000	8.874649000000
H	-3.324252000000	6.862983000000	7.587824000000
H	-5.455282000000	3.868181000000	9.871692000000
C	-5.081483000000	6.579655000000	9.680890000000
H	-4.562044000000	6.725939000000	10.652167000000
H	-5.067979000000	7.551433000000	9.151086000000
H	-6.133849000000	6.324005000000	9.914739000000
C	-4.370561000000	1.631584000000	8.634787000000
H	-4.227003000000	1.162111000000	7.630545000000
C	-1.858750000000	5.149770000000	5.989044000000
H	-1.958065000000	4.340463000000	5.231148000000
C	-5.822337000000	1.363391000000	9.022320000000
C	-6.171700000000	0.490541000000	10.069289000000
C	-6.859627000000	1.958089000000	8.268052000000
C	-7.519852000000	0.220006000000	10.357646000000
H	-5.381116000000	0.014427000000	10.666934000000
C	-8.204453000000	1.686993000000	8.553310000000

H	-6.606383000000	2.662570000000	7.459852000000
C	-8.540141000000	0.814414000000	9.602146000000
H	-7.771434000000	-0.463224000000	11.182799000000
H	-8.997117000000	2.163812000000	7.956874000000
H	-9.595359000000	0.602399000000	9.830031000000
C	-3.341271000000	0.953353000000	9.528969000000
C	-2.705450000000	-0.226048000000	9.094162000000
C	-2.990597000000	1.487363000000	10.783955000000
C	-1.732489000000	-0.855889000000	9.885894000000
H	-2.971109000000	-0.646582000000	8.111057000000
C	-2.024546000000	0.857214000000	11.582654000000
H	-3.474189000000	2.412438000000	11.133479000000
C	-1.388185000000	-0.312993000000	11.134116000000
H	-1.241230000000	-1.772396000000	9.525766000000
H	-1.762181000000	1.284778000000	12.562152000000
H	-0.627136000000	-0.802940000000	11.759684000000
C	-2.180761000000	6.431834000000	5.219238000000
C	-3.495602000000	6.647897000000	4.754829000000
C	-1.168221000000	7.313295000000	4.798200000000
C	-3.785932000000	7.695635000000	3.874004000000
H	-4.297862000000	5.958979000000	5.061408000000
C	-1.454256000000	8.362847000000	3.908105000000
H	-0.130058000000	7.162620000000	5.124345000000
C	-2.761546000000	8.555003000000	3.436203000000
H	-4.815806000000	7.836193000000	3.512569000000
H	-0.641058000000	9.031163000000	3.585135000000
H	-2.989744000000	9.381556000000	2.745435000000
C	-0.418534000000	5.080110000000	6.481008000000
C	0.507651000000	4.250830000000	5.822829000000
C	0.013704000000	5.835293000000	7.590541000000
C	1.840117000000	4.172203000000	6.262656000000
H	0.198240000000	3.642875000000	4.961500000000
C	1.344408000000	5.767033000000	8.029707000000
H	-0.702954000000	6.484234000000	8.116628000000
C	2.263652000000	4.931679000000	7.367543000000
H	2.551549000000	3.514739000000	5.746925000000
H	1.666480000000	6.365666000000	8.895027000000
H	3.307702000000	4.861714000000	7.707580000000
C	0.526265000000	0.174031000000	6.432252000000
C	0.731877000000	-0.933203000000	5.556500000000
C	1.651544000000	0.667392000000	7.153793000000
C	1.995062000000	-1.449257000000	5.330015000000
H	-0.132475000000	-1.355541000000	5.026748000000
C	2.917654000000	0.134386000000	6.956448000000
H	1.551322000000	1.520270000000	7.831453000000
C	3.114476000000	-0.890653000000	5.999473000000
H	2.143770000000	-2.277514000000	4.621503000000
H	3.795689000000	0.554723000000	7.465792000000
O	4.366090000000	-1.283574000000	5.732393000000
H	4.367576000000	-1.714924000000	4.844600000000
C	-4.824081000000	2.397612000000	4.717059000000
H	-4.460439000000	3.132723000000	5.466118000000

H	-5.908160000000	2.588545000000	4.640204000000
C	-4.125351000000	2.488056000000	3.365001000000
H	-4.158873000000	3.552475000000	3.042350000000
H	-3.029280000000	2.286666000000	3.456184000000
C	-4.259132000000	-0.420124000000	5.133727000000
H	-4.049678000000	-1.058136000000	6.011166000000
H	-4.030076000000	-0.898995000000	4.163740000000
C	-5.320040000000	0.559914000000	5.189266000000
H	-5.993648000000	0.555217000000	4.318961000000
H	-5.876709000000	0.676095000000	6.135927000000

P2$_{C2H2}$

Ni	0.627782000000	0.690370000000	0.913101000000
N	-1.083336000000	-0.237182000000	0.507092000000
C	-1.107486000000	1.202939000000	4.951863000000
H	-1.126576000000	0.923110000000	6.010799000000
H	-1.498891000000	0.341573000000	4.395747000000
C	-2.006156000000	2.386705000000	4.730994000000
C	-2.698319000000	3.007728000000	5.685289000000
H	-3.329548000000	3.860976000000	5.455804000000
H	-2.657585000000	2.684829000000	6.723425000000
C	0.104350000000	-0.449378000000	-1.469756000000
C	-1.170753000000	-0.722292000000	-0.678485000000
H	-2.096322000000	2.753522000000	3.710087000000
O	0.958723000000	0.257711000000	-0.889841000000
C	-2.380577000000	-1.362478000000	-1.264173000000
H	-2.604848000000	-0.923648000000	-2.240282000000
H	-2.225327000000	-2.437722000000	-1.383513000000
H	-3.233330000000	-1.222902000000	-0.601993000000
C	-2.173958000000	-0.233707000000	1.430426000000
C	-2.299890000000	-1.291091000000	2.334656000000
C	-2.984513000000	0.911339000000	1.491175000000
C	-3.328747000000	-1.224365000000	3.274339000000
C	-3.990133000000	0.934032000000	2.451740000000
C	-4.184739000000	-0.126067000000	3.342243000000
H	-3.430320000000	-2.026697000000	3.999772000000
H	-4.612699000000	1.818402000000	2.546074000000
C	-5.241773000000	-0.027076000000	4.409831000000
H	-5.403395000000	-0.987169000000	4.905258000000
H	-4.935482000000	0.700846000000	5.169617000000
H	-6.195912000000	0.310774000000	3.995465000000
C	-2.699489000000	2.074989000000	0.537975000000
H	-1.605380000000	2.153674000000	0.481421000000
C	-1.282281000000	-2.430220000000	2.300995000000
H	-0.317621000000	-1.966735000000	2.054322000000
C	-3.175732000000	3.429994000000	1.044853000000
C	-4.510355000000	3.837033000000	0.960325000000
C	-2.250144000000	4.303423000000	1.620425000000
C	-4.911284000000	5.068854000000	1.470431000000
H	-5.246591000000	3.195461000000	0.486096000000
C	-2.644952000000	5.538004000000	2.131139000000
H	-1.200295000000	4.021808000000	1.664935000000
C	-3.981459000000	5.921305000000	2.063589000000

H	-5.952517000000	5.366448000000	1.397694000000
H	-1.907997000000	6.199294000000	2.575636000000
H	-4.295311000000	6.882234000000	2.457950000000
C	-3.162526000000	1.787148000000	-0.884672000000
C	-2.313784000000	2.085435000000	-1.954542000000
C	-4.413275000000	1.228514000000	-1.155770000000
C	-2.701071000000	1.826466000000	-3.267588000000
H	-1.340012000000	2.528927000000	-1.756269000000
C	-4.811985000000	0.983047000000	-2.467890000000
H	-5.073690000000	0.961476000000	-0.334688000000
C	-3.955035000000	1.275521000000	-3.528043000000
H	-2.027958000000	2.061481000000	-4.086355000000
H	-5.790056000000	0.554140000000	-2.662094000000
H	-4.263771000000	1.078866000000	-4.549623000000
C	-1.088922000000	-3.071040000000	3.669812000000
C	-0.279397000000	-2.409802000000	4.598683000000
C	-1.708309000000	-4.261587000000	4.049224000000
C	-0.103379000000	-2.912161000000	5.883137000000
H	0.219233000000	-1.487214000000	4.309244000000
C	-1.534233000000	-4.770036000000	5.336801000000
H	-2.324251000000	-4.805808000000	3.340298000000
C	-0.736609000000	-4.096925000000	6.257774000000
H	0.531333000000	-2.385571000000	6.589163000000
H	-2.021287000000	-5.698869000000	5.616264000000
H	-0.600690000000	-4.496153000000	7.257550000000
C	-1.562006000000	-3.422389000000	1.183416000000
C	-0.505921000000	-3.881459000000	0.391448000000
C	-2.853561000000	-3.881625000000	0.913439000000
C	-0.733146000000	-4.772604000000	-0.655739000000
H	0.506000000000	-3.540025000000	0.598568000000
C	-3.084801000000	-4.781253000000	-0.125149000000
H	-3.687871000000	-3.521531000000	1.509415000000
C	-2.026384000000	-5.224416000000	-0.918085000000
H	0.099782000000	-5.121006000000	-1.259040000000
H	-4.093408000000	-5.132327000000	-0.319246000000
H	-2.207086000000	-5.924600000000	-1.727468000000
C	0.346948000000	-0.872883000000	-2.833503000000
C	1.264634000000	-0.119031000000	-3.594004000000
C	-0.235172000000	-2.017316000000	-3.417107000000
C	1.560095000000	-0.464397000000	-4.896871000000
H	1.726875000000	0.754702000000	-3.148057000000
C	0.076007000000	-2.383946000000	-4.710036000000
H	-0.895148000000	-2.657807000000	-2.847429000000
C	0.964759000000	-1.602814000000	-5.462716000000
H	2.251612000000	0.138504000000	-5.478358000000
H	-0.349604000000	-3.273236000000	-5.160246000000
O	1.207412000000	-2.002771000000	-6.717656000000
H	1.836797000000	-1.423805000000	-7.160991000000
C	0.348611000000	1.451458000000	4.535207000000
H	0.986043000000	0.647743000000	4.921972000000
H	0.710155000000	2.381484000000	4.990765000000
C	0.584736000000	1.570447000000	3.021849000000

H	0.310581000000	0.531794000000	2.652516000000
H	-0.124267000000	2.291530000000	2.595436000000
C	2.039764000000	1.936011000000	2.657165000000
H	2.713466000000	1.323734000000	3.265539000000
H	2.219496000000	2.983025000000	2.924278000000
C	2.220103000000	1.639726000000	1.176942000000
H	2.193264000000	2.520253000000	0.525603000000
H	3.083904000000	1.018551000000	0.926248000000

vTMS

C	-3.082722000000	0.087391000000	-0.036588000000
H	-2.987491000000	-0.902122000000	0.404081000000
H	-4.093979000000	0.453946000000	-0.199325000000
C	-2.007418000000	0.813879000000	-0.356529000000
H	-2.156740000000	1.801503000000	-0.792098000000
Si	-0.282519000000	0.218828000000	-0.090285000000
O	0.561795000000	1.336756000000	0.788351000000
O	0.465101000000	0.005659000000	-1.550578000000
O	-0.330415000000	-1.215792000000	0.730844000000
C	1.572197000000	1.030963000000	1.727745000000
H	1.767073000000	1.927362000000	2.321194000000
H	1.261009000000	0.222137000000	2.399072000000
H	2.504153000000	0.737125000000	1.226291000000
C	0.531527000000	-2.312293000000	0.503938000000
H	0.659043000000	-2.506444000000	-0.567315000000
H	1.517906000000	-2.136819000000	0.953360000000
H	0.090371000000	-3.194891000000	0.973358000000
C	1.848237000000	0.185215000000	-1.779031000000
H	2.434890000000	-0.627819000000	-1.330262000000
H	2.017591000000	0.177470000000	-2.858341000000
H	2.198673000000	1.141046000000	-1.372168000000

I_{vTMS}

O	6.070902000000	1.869365000000	-0.479331000000
Si	4.544225000000	1.950202000000	0.113813000000
O	4.329314000000	0.695266000000	1.135093000000
Si	3.302766000000	-0.466543000000	1.646390000000
O	3.279139000000	-1.788714000000	0.654167000000
O	3.399149000000	1.767096000000	-1.093060000000
O	4.223534000000	3.391261000000	0.865268000000
Si	4.689955000000	4.342291000000	2.111097000000
O	3.404545000000	4.887079000000	2.952306000000
Si	2.271173000000	6.086028000000	3.034384000000
O	0.821048000000	5.309314000000	2.817968000000
O	1.790372000000	0.079192000000	1.878062000000
Si	0.881993000000	1.279805000000	2.561250000000
O	-0.684029000000	0.774435000000	2.654838000000
O	4.025160000000	-0.967240000000	3.092224000000
O	5.701676000000	5.536222000000	1.662206000000
Si	5.933060000000	6.760346000000	0.578787000000
O	5.153729000000	8.046537000000	1.363467000000
O	5.344969000000	6.474189000000	-0.917770000000

O	7.562829000000	6.995655000000	0.393837000000
O	5.615561000000	3.437673000000	3.165059000000
Si	5.396804000000	2.417510000000	4.465604000000
O	4.040066000000	1.480942000000	4.343393000000
O	0.913419000000	2.675767000000	1.636154000000
Si	0.176011000000	3.193479000000	0.222935000000
O	-1.376709000000	3.665831000000	0.492376000000
Si	-2.243453000000	4.945980000000	1.136617000000
O	-3.841196000000	4.536532000000	1.155527000000
O	0.176026000000	1.989456000000	-0.914973000000
O	1.109786000000	4.491128000000	-0.153702000000
Si	1.420690000000	5.623218000000	-1.321493000000
O	2.597984000000	6.631110000000	-0.717703000000
O	2.147652000000	4.838204000000	-2.603190000000
O	0.055407000000	6.382675000000	-1.833304000000
O	1.368300000000	1.699216000000	4.071464000000
O	6.792804000000	1.531770000000	4.515018000000
Si	7.815971000000	0.673767000000	3.543715000000
O	6.876132000000	-0.426072000000	2.667646000000
O	8.627066000000	1.555608000000	2.426861000000
Si	9.058832000000	3.091777000000	2.009878000000
O	10.687881000000	3.270031000000	1.839677000000
O	8.333158000000	3.247729000000	0.502421000000
O	8.513745000000	4.219294000000	3.098014000000
O	8.810481000000	-0.098833000000	4.604070000000
O	5.168030000000	3.266782000000	5.861723000000
O	-1.917905000000	6.215794000000	0.066710000000
O	-1.881767000000	5.339764000000	2.686818000000
O	2.526040000000	7.259173000000	1.884630000000
O	2.322707000000	6.866810000000	4.489461000000
H	6.814158000000	2.416880000000	-0.123802000000
H	3.420734000000	2.477613000000	-1.765644000000
H	5.010840000000	-1.006040000000	2.961426000000
H	-1.006725000000	-0.014861000000	2.160797000000
H	2.343316000000	1.756955000000	4.244085000000
H	-4.135506000000	3.981926000000	0.401358000000
H	-0.922439000000	5.513461000000	2.858690000000
H	3.143207000000	-1.629084000000	-0.295847000000
H	0.945034000000	4.368984000000	2.531953000000
H	2.134312000000	6.326589000000	5.276349000000
H	5.876216000000	3.881146000000	6.119703000000
H	4.127492000000	0.544998000000	4.019529000000
H	11.170230000000	3.641187000000	2.598103000000
H	8.538940000000	4.072032000000	0.022195000000
H	7.535438000000	4.222203000000	3.186034000000
H	5.247864000000	8.910053000000	0.920862000000
H	8.064043000000	7.085114000000	1.223542000000
H	6.901406000000	-0.259084000000	1.705496000000
H	9.514742000000	-0.651267000000	4.224391000000
H	-0.710562000000	6.358398000000	-1.203729000000
H	1.761499000000	5.036753000000	-3.473260000000
H	1.093380000000	1.736007000000	-1.155003000000

H	-2.316512000000	7.066791000000	0.323662000000
H	3.418791000000	7.697836000000	1.907702000000
H	4.351984000000	6.504713000000	-0.996933000000
H	2.444988000000	6.927134000000	0.222008000000
Ni	-4.254604000000	-0.732916000000	3.532060000000
N	-4.198518000000	0.002670000000	5.369626000000
C	-4.722199000000	2.605780000000	-1.335876000000
H	-5.772133000000	2.870049000000	-1.508373000000
H	-3.978746000000	3.067310000000	-1.993429000000
C	-4.361579000000	1.723267000000	-0.382727000000
C	-5.319239000000	0.990939000000	0.518414000000
H	-6.345610000000	1.375565000000	0.380135000000
H	-5.342298000000	-0.071537000000	0.206569000000
C	-2.033208000000	-0.878583000000	5.170631000000
C	-3.070531000000	-0.202743000000	6.025212000000
H	-3.295547000000	1.489261000000	-0.250079000000
O	-2.454531000000	-1.238047000000	4.019032000000
C	-2.874228000000	0.108182000000	7.475563000000
H	-2.412115000000	-0.748566000000	7.991334000000
H	-2.222911000000	0.990752000000	7.598338000000
H	-3.832213000000	0.336592000000	7.958375000000
C	-5.321310000000	0.583107000000	6.070048000000
C	-5.495611000000	1.990771000000	6.079026000000
C	-6.262127000000	-0.277245000000	6.692591000000
C	-6.586599000000	2.513957000000	6.795857000000
C	-7.343161000000	0.303092000000	7.377208000000
C	-7.511416000000	1.695555000000	7.466532000000
H	-6.734498000000	3.598836000000	6.796562000000
H	-8.086952000000	-0.358991000000	7.833934000000
C	-8.655196000000	2.294238000000	8.250586000000
H	-8.360371000000	2.478458000000	9.299349000000
H	-8.972857000000	3.260091000000	7.826980000000
H	-9.527135000000	1.621419000000	8.269925000000
C	-6.095144000000	-1.799686000000	6.634617000000
H	-5.546633000000	-2.008250000000	5.697138000000
C	-4.524038000000	2.918627000000	5.333930000000
H	-4.175077000000	2.358295000000	4.444608000000
C	-7.439144000000	-2.532600000000	6.503628000000
C	-7.868810000000	-3.511255000000	7.417370000000
C	-8.268517000000	-2.235370000000	5.399767000000
C	-9.096274000000	-4.172532000000	7.236825000000
H	-7.242316000000	-3.765554000000	8.276990000000
C	-9.491087000000	-2.894018000000	5.216521000000
H	-7.957653000000	-1.462933000000	4.686654000000
C	-9.911124000000	-3.868145000000	6.138278000000
H	-9.412087000000	-4.929288000000	7.961471000000
H	-10.119562000000	-2.645408000000	4.355526000000
H	-10.865981000000	-4.383470000000	5.998777000000
C	-5.206798000000	-2.333979000000	7.761819000000
C	-4.229106000000	-3.307356000000	7.474301000000
C	-5.341364000000	-1.895703000000	9.094189000000
C	-3.400823000000	-3.823956000000	8.483113000000

H	-4.119984000000	-3.672400000000	6.446369000000
C	-4.519316000000	-2.412489000000	10.107578000000
H	-6.091378000000	-1.137840000000	9.342240000000
C	-3.542498000000	-3.375227000000	9.805564000000
H	-2.647915000000	-4.578922000000	8.236118000000
H	-4.642367000000	-2.060775000000	11.136613000000
H	-2.900104000000	-3.775568000000	10.595478000000
C	-5.207882000000	4.188171000000	4.797647000000
C	-6.383253000000	4.067458000000	4.024295000000
C	-4.679584000000	5.473728000000	5.006879000000
C	-7.016774000000	5.195585000000	3.488508000000
H	-6.823318000000	3.077807000000	3.857115000000
C	-5.313631000000	6.607724000000	4.471906000000
H	-3.758291000000	5.596841000000	5.581570000000
C	-6.483853000000	6.475557000000	3.713465000000
H	-7.929706000000	5.075988000000	2.896489000000
H	-4.882719000000	7.597789000000	4.650314000000
H	-6.978277000000	7.359408000000	3.299262000000
C	-3.261794000000	3.194218000000	6.154813000000
C	-1.994274000000	3.004677000000	5.571240000000
C	-3.330738000000	3.627131000000	7.494566000000
C	-0.819242000000	3.221923000000	6.309861000000
H	-1.920698000000	2.678228000000	4.528091000000
C	-2.160408000000	3.858023000000	8.233567000000
H	-4.307140000000	3.777568000000	7.966645000000
C	-0.900492000000	3.648496000000	7.645385000000
H	0.148818000000	3.037615000000	5.833430000000
H	-2.232218000000	4.196643000000	9.271936000000
H	0.011204000000	3.817616000000	8.227449000000
C	-0.651937000000	-1.166996000000	5.514191000000
C	0.052838000000	-2.035659000000	4.627700000000
C	0.071281000000	-0.570067000000	6.584801000000
C	1.411748000000	-2.256845000000	4.775625000000
H	-0.497156000000	-2.529372000000	3.820669000000
C	1.434378000000	-0.790928000000	6.739212000000
H	-0.411162000000	0.127465000000	7.267354000000
C	2.121937000000	-1.603957000000	5.812412000000
H	1.943505000000	-2.919906000000	4.085363000000
H	2.001182000000	-0.295555000000	7.531261000000
O	3.461289000000	-1.725078000000	5.953098000000
H	3.830476000000	-2.101383000000	5.126207000000
C	-5.822292000000	0.154187000000	2.904049000000
H	-6.318588000000	0.642126000000	3.756624000000
H	-6.528907000000	-0.487306000000	2.353417000000
C	-4.962032000000	1.056058000000	2.022178000000
H	-4.986442000000	2.101343000000	2.376956000000
H	-3.867634000000	0.777049000000	2.120054000000
C	-4.889018000000	-2.621286000000	3.264335000000
H	-4.282945000000	-3.233695000000	3.943731000000
H	-5.974282000000	-2.698261000000	3.402758000000
C	-4.338680000000	-2.131822000000	2.057523000000
H	-5.077859000000	-1.807398000000	1.309985000000

Si	-2.699970000000	-2.655826000000	1.310780000000
O	-1.793684000000	-1.280793000000	0.961990000000
O	-3.003297000000	-3.515282000000	-0.088516000000
O	-1.791710000000	-3.672566000000	2.261254000000
C	-0.750595000000	-1.359068000000	-0.048988000000
H	-0.584582000000	-0.338916000000	-0.427177000000
H	0.179021000000	-1.746522000000	0.399969000000
H	-1.055316000000	-2.017600000000	-0.878581000000
C	-1.924745000000	-5.106353000000	2.269993000000
H	-1.927112000000	-5.512255000000	1.245470000000
H	-1.063374000000	-5.511576000000	2.822956000000
H	-2.854004000000	-5.413802000000	2.782270000000
C	-3.837711000000	-3.091831000000	-1.173350000000
H	-3.718623000000	-3.821824000000	-1.988676000000
H	-4.902005000000	-3.072897000000	-0.876113000000
H	-3.549837000000	-2.092283000000	-1.548597000000

TS$_{vTMS}$

O	6.064781000000	1.936303000000	-0.496190000000
Si	4.544225000000	1.950202000000	0.113813000000
O	4.376242000000	0.637903000000	1.079707000000
Si	3.302766000000	-0.466543000000	1.646390000000
O	3.258018000000	-1.837419000000	0.723917000000
O	3.391724000000	1.783818000000	-1.086614000000
O	4.183224000000	3.356170000000	0.912477000000
Si	4.689955000000	4.342291000000	2.111097000000
O	3.421787000000	4.903171000000	2.965153000000
Si	2.271173000000	6.086028000000	3.034384000000
O	0.823586000000	5.307575000000	2.814131000000
O	1.815757000000	0.146613000000	1.816313000000
Si	0.881993000000	1.279805000000	2.561250000000
O	-0.648741000000	0.662191000000	2.619772000000
O	3.957011000000	-0.912860000000	3.133424000000
O	5.712945000000	5.509264000000	1.630519000000
Si	5.933060000000	6.760346000000	0.578787000000
O	5.164196000000	8.031171000000	1.399001000000
O	5.336921000000	6.510059000000	-0.919625000000
O	7.562159000000	7.000983000000	0.392071000000
O	5.621203000000	3.450028000000	3.176899000000
Si	5.396804000000	2.417510000000	4.465604000000
O	4.051749000000	1.470525000000	4.358849000000
O	0.811449000000	2.697200000000	1.689304000000
Si	0.176011000000	3.193479000000	0.222935000000
O	-1.391443000000	3.687973000000	0.433859000000
Si	-2.243453000000	4.945980000000	1.136617000000
O	-3.865785000000	4.666362000000	1.077243000000
O	0.208489000000	1.981612000000	-0.894255000000
O	1.127161000000	4.488180000000	-0.140287000000
Si	1.420690000000	5.623218000000	-1.321493000000
O	2.596299000000	6.634910000000	-0.720177000000
O	2.150085000000	4.858519000000	-2.611713000000

O	0.041289000000	6.363984000000	-1.822540000000
O	1.403073000000	1.681357000000	4.068905000000
O	6.800520000000	1.543245000000	4.520054000000
Si	7.815971000000	0.673767000000	3.543715000000
O	6.885734000000	-0.446848000000	2.695362000000
O	8.609452000000	1.558040000000	2.415279000000
Si	9.058832000000	3.091777000000	2.009878000000
O	10.691217000000	3.260440000000	1.857807000000
O	8.348012000000	3.259262000000	0.497769000000
O	8.512327000000	4.218805000000	3.099039000000
O	8.826824000000	-0.081204000000	4.604013000000
O	5.159924000000	3.270426000000	5.866794000000
O	-1.822801000000	6.292101000000	0.208215000000
O	-1.896785000000	5.177764000000	2.728380000000
O	2.535235000000	7.254544000000	1.880454000000
O	2.303212000000	6.864780000000	4.490440000000
H	6.813551000000	2.461052000000	-0.120007000000
H	3.384548000000	2.517490000000	-1.734398000000
H	4.927299000000	-1.083332000000	3.042884000000
H	-0.685137000000	-0.286597000000	2.900841000000
H	2.381285000000	1.732313000000	4.231406000000
H	-4.204275000000	3.908289000000	0.555578000000
H	-0.957623000000	5.434761000000	2.902033000000
H	3.063438000000	-1.726409000000	-0.223055000000
H	0.936340000000	4.371503000000	2.508534000000
H	2.045095000000	6.329807000000	5.266612000000
H	5.831229000000	3.946617000000	6.063868000000
H	4.116327000000	0.541145000000	3.989466000000
H	11.164765000000	3.634869000000	2.620109000000
H	8.562090000000	4.082795000000	0.020142000000
H	7.533724000000	4.214432000000	3.187192000000
H	5.259608000000	8.902628000000	0.972346000000
H	8.065308000000	7.076242000000	1.221978000000
H	6.827407000000	-0.249385000000	1.740337000000
H	9.535152000000	-0.622636000000	4.215779000000
H	-0.695577000000	6.388009000000	-1.160169000000
H	1.680073000000	4.955598000000	-3.457540000000
H	1.123246000000	1.688831000000	-1.093978000000
H	-2.245364000000	7.126982000000	0.477525000000
H	3.431225000000	7.686640000000	1.906215000000
H	4.343116000000	6.534412000000	-0.998871000000
H	2.447080000000	6.921237000000	0.223741000000
Ni	-2.581867000000	0.617943000000	5.381636000000
N	-2.121716000000	1.683507000000	7.007048000000
C	-5.611029000000	2.224845000000	-0.177413000000
H	-6.605068000000	2.602315000000	0.088614000000
H	-5.312151000000	2.310787000000	-1.226753000000
C	-4.803476000000	1.653095000000	0.737676000000
C	-5.139030000000	1.429677000000	2.191294000000
H	-6.142558000000	1.833703000000	2.419254000000
H	-5.200080000000	0.332465000000	2.330505000000
C	-0.188406000000	0.432677000000	6.669009000000

C	-0.938264000000	1.411632000000	7.518694000000
H	-3.814601000000	1.289930000000	0.420121000000
O	-0.838324000000	-0.028678000000	5.671682000000
C	-0.445914000000	1.893486000000	8.847465000000
H	0.012472000000	1.062238000000	9.406175000000
H	0.309739000000	2.687888000000	8.726607000000
H	-1.271154000000	2.308984000000	9.439959000000
C	-3.028535000000	2.527160000000	7.741283000000
C	-2.999715000000	3.934782000000	7.566424000000
C	-3.972940000000	1.908739000000	8.601597000000
C	-3.878635000000	4.707690000000	8.346963000000
C	-4.831077000000	2.732409000000	9.348120000000
C	-4.782664000000	4.134965000000	9.258243000000
H	-3.877943000000	5.793849000000	8.208701000000
H	-5.575118000000	2.261022000000	9.999346000000
C	-5.684346000000	5.000189000000	10.106782000000
H	-5.208034000000	5.234079000000	11.075908000000
H	-5.906323000000	5.958865000000	9.612095000000
H	-6.638560000000	4.495472000000	10.326480000000
C	-4.027758000000	0.380211000000	8.716989000000
H	-3.698329000000	-0.009662000000	7.732105000000
C	-2.052499000000	4.601153000000	6.551737000000
H	-1.947636000000	3.899223000000	5.700684000000
C	-5.452112000000	-0.156843000000	8.925145000000
C	-5.774538000000	-1.080915000000	9.935697000000
C	-6.474209000000	0.243645000000	8.036033000000
C	-7.080083000000	-1.587216000000	10.057939000000
H	-5.002584000000	-1.412181000000	10.635758000000
C	-7.775929000000	-0.260472000000	8.154446000000
H	-6.250453000000	0.978935000000	7.254826000000
C	-8.084958000000	-1.181145000000	9.169730000000
H	-7.307656000000	-2.302355000000	10.854360000000
H	-8.552383000000	0.069180000000	7.456914000000
H	-9.101060000000	-1.574639000000	9.266778000000
C	-3.005733000000	-0.162377000000	9.718901000000
C	-2.153132000000	-1.218193000000	9.338704000000
C	-2.894014000000	0.358079000000	11.023397000000
C	-1.210072000000	-1.740988000000	10.238758000000
H	-2.225650000000	-1.630309000000	8.324704000000
C	-1.955653000000	-0.165118000000	11.926047000000
H	-3.544328000000	1.181637000000	11.335983000000
C	-1.108158000000	-1.215627000000	11.536285000000
H	-0.554347000000	-2.559446000000	9.924551000000
H	-1.886383000000	0.248579000000	12.936878000000
H	-0.375731000000	-1.621910000000	12.240247000000
C	-2.648859000000	5.890586000000	5.958714000000
C	-3.913293000000	5.831415000000	5.335337000000
C	-1.970371000000	7.121921000000	5.969177000000
C	-4.487682000000	6.966434000000	4.752831000000
H	-4.457105000000	4.880839000000	5.307689000000
C	-2.543022000000	8.264943000000	5.383568000000
H	-0.983028000000	7.201077000000	6.432075000000

C	-3.803557000000	8.193628000000	4.776830000000
H	-5.467826000000	6.891937000000	4.271709000000
H	-1.995053000000	9.212013000000	5.406056000000
H	-4.251365000000	9.084037000000	4.325095000000
C	-0.639221000000	4.783460000000	7.109337000000
C	0.471246000000	4.307909000000	6.382858000000
C	-0.410808000000	5.426350000000	8.343103000000
C	1.779475000000	4.466549000000	6.878037000000
H	0.319774000000	3.789847000000	5.429398000000
C	0.892082000000	5.598799000000	8.835770000000
H	-1.261633000000	5.793661000000	8.926258000000
C	1.992507000000	5.117914000000	8.105976000000
H	2.632217000000	4.062768000000	6.320086000000
H	1.047745000000	6.106275000000	9.792746000000
H	3.008464000000	5.243638000000	8.492827000000
C	1.145613000000	-0.084230000000	6.933339000000
C	1.462196000000	-1.376235000000	6.432809000000
C	2.161528000000	0.651258000000	7.600477000000
C	2.727320000000	-1.921540000000	6.612418000000
H	0.677490000000	-1.944243000000	5.926820000000
C	3.437336000000	0.122875000000	7.759545000000
H	1.977300000000	1.671551000000	7.939453000000
C	3.726375000000	-1.169714000000	7.272503000000
H	2.954810000000	-2.925766000000	6.237634000000
H	4.234042000000	0.701262000000	8.233591000000
O	4.987871000000	-1.631213000000	7.466723000000
H	5.086146000000	-2.520661000000	7.074731000000
C	-4.184890000000	1.741363000000	4.637531000000
H	-3.509546000000	2.434321000000	5.176082000000
H	-5.181350000000	1.885957000000	5.080581000000
C	-4.081054000000	2.034900000000	3.140547000000
H	-4.122581000000	3.136766000000	3.040719000000
H	-3.066709000000	1.765839000000	2.790402000000
C	-4.331854000000	-0.258238000000	4.810843000000
H	-4.598430000000	-0.637696000000	5.809767000000
H	-5.243554000000	-0.108603000000	4.221384000000
C	-3.154898000000	-0.776103000000	4.143171000000
H	-3.077383000000	-0.439681000000	3.094702000000
Si	-2.266544000000	-2.393771000000	4.333577000000
O	-0.903871000000	-2.092849000000	3.384479000000
O	-3.094952000000	-3.711419000000	3.728024000000
O	-1.929900000000	-2.800276000000	5.910175000000
C	0.041246000000	-3.104648000000	2.988703000000
H	0.599124000000	-2.726016000000	2.119101000000
H	0.755825000000	-3.309334000000	3.805584000000
H	-0.469108000000	-4.043613000000	2.713108000000
C	-2.034218000000	-4.125028000000	6.468542000000
H	-2.765121000000	-4.739725000000	5.919862000000
H	-1.048775000000	-4.622350000000	6.443363000000
H	-2.355234000000	-4.023114000000	7.517527000000
C	-3.618619000000	-3.793718000000	2.394944000000
H	-3.944117000000	-4.832204000000	2.229771000000

H	-4.490730000000	-3.126898000000	2.270596000000
H	-2.856748000000	-3.532733000000	1.638171000000

P_{vTMS}

O	6.067807000000	1.927325000000	-0.488289000000
Si	4.544225000000	1.950202000000	0.113813000000
O	4.365541000000	0.648494000000	1.087066000000
Si	3.302766000000	-0.466543000000	1.646390000000
O	3.263022000000	-1.831922000000	0.716419000000
O	3.395651000000	1.786525000000	-1.090543000000
O	4.184382000000	3.361507000000	0.907425000000
Si	4.689955000000	4.342291000000	2.111097000000
O	3.429815000000	4.910882000000	2.972208000000
Si	2.271173000000	6.086028000000	3.034384000000
O	0.834108000000	5.290703000000	2.804444000000
O	1.810190000000	0.125824000000	1.838405000000
Si	0.881993000000	1.279805000000	2.561250000000
O	-0.659761000000	0.704454000000	2.593138000000
O	3.980990000000	-0.918321000000	3.123202000000
O	5.715015000000	5.508538000000	1.629826000000
Si	5.933060000000	6.760346000000	0.578787000000
O	5.160885000000	8.029822000000	1.399021000000
O	5.337337000000	6.511326000000	-0.920173000000
O	7.561436000000	7.005436000000	0.392632000000
O	5.621106000000	3.446068000000	3.173280000000
Si	5.396804000000	2.417510000000	4.465604000000
O	4.053833000000	1.465421000000	4.360682000000
O	0.844464000000	2.692891000000	1.674580000000
Si	0.176011000000	3.193479000000	0.222935000000
O	-1.389695000000	3.680787000000	0.456214000000
Si	-2.243453000000	4.945980000000	1.136617000000
O	-3.859303000000	4.620429000000	1.212829000000
O	0.205512000000	1.995314000000	-0.908437000000
O	1.112903000000	4.500679000000	-0.135609000000
Si	1.420690000000	5.623218000000	-1.321493000000
O	2.594257000000	6.638244000000	-0.722089000000
O	2.159281000000	4.848113000000	-2.601212000000
O	0.047799000000	6.366954000000	-1.837565000000
O	1.395956000000	1.680216000000	4.072039000000
O	6.800952000000	1.542879000000	4.519164000000
Si	7.815971000000	0.673767000000	3.543715000000
O	6.883706000000	-0.440622000000	2.687163000000
O	8.616660000000	1.557100000000	2.419709000000
Si	9.058832000000	3.091777000000	2.009878000000
O	10.690415000000	3.266724000000	1.855992000000
O	8.348547000000	3.253361000000	0.496841000000
O	8.508323000000	4.219272000000	3.096176000000
O	8.820585000000	-0.089269000000	4.603974000000
O	5.155362000000	3.271235000000	5.863732000000
O	-1.911593000000	6.240838000000	0.101477000000
O	-1.857870000000	5.272032000000	2.699006000000
O	2.535978000000	7.253723000000	1.880154000000

O	2.280519000000	6.865594000000	4.489469000000
H	6.815443000000	2.456194000000	-0.115468000000
H	3.388995000000	2.522949000000	-1.735317000000
H	4.955912000000	-1.058831000000	3.019376000000
H	-0.765469000000	-0.234367000000	2.889668000000
H	2.373286000000	1.725044000000	4.239332000000
H	-4.321281000000	4.193029000000	0.468152000000
H	-0.898259000000	5.466734000000	2.850498000000
H	3.004241000000	-1.726483000000	-0.215582000000
H	0.957383000000	4.355711000000	2.496499000000
H	1.975293000000	6.344104000000	5.257545000000
H	5.816837000000	3.957350000000	6.058826000000
H	4.124202000000	0.540607000000	3.982074000000
H	11.164193000000	3.638406000000	2.619493000000
H	8.563793000000	4.074629000000	0.015923000000
H	7.529697000000	4.212001000000	3.184520000000
H	5.256750000000	8.901845000000	0.973620000000
H	8.065248000000	7.072640000000	1.222847000000
H	6.843910000000	-0.248508000000	1.730074000000
H	9.526092000000	-0.635083000000	4.216778000000
H	-0.710714000000	6.358511000000	-1.199900000000
H	1.691390000000	4.934840000000	-3.449347000000
H	1.119664000000	1.701206000000	-1.108558000000
H	-2.305208000000	7.088202000000	0.377068000000
H	3.433277000000	7.684148000000	1.902738000000
H	4.343754000000	6.535669000000	-0.999944000000
H	2.445599000000	6.923690000000	0.221998000000
Ni	-2.908873000000	0.873160000000	5.366228000000
N	-2.452619000000	1.899919000000	6.953852000000
C	-6.654630000000	3.219348000000	-0.048477000000
H	-7.316342000000	3.973040000000	0.393281000000
H	-6.493596000000	3.273845000000	-1.129761000000
C	-6.093462000000	2.249910000000	0.698838000000
C	-6.287111000000	2.056341000000	2.181462000000
H	-7.036946000000	2.770905000000	2.565018000000
H	-6.710263000000	1.043538000000	2.336683000000
C	-0.568353000000	0.584006000000	6.648299000000
C	-1.293645000000	1.578238000000	7.495074000000
H	-5.438618000000	1.514365000000	0.208015000000
O	-1.236293000000	0.145521000000	5.640434000000
C	-0.823878000000	2.040474000000	8.837517000000
H	-0.424775000000	1.191322000000	9.414962000000
H	-0.033035000000	2.803782000000	8.742376000000
H	-1.652682000000	2.492534000000	9.397918000000
C	-3.369611000000	2.758729000000	7.650699000000
C	-3.302989000000	4.164064000000	7.475973000000
C	-4.373186000000	2.155001000000	8.452037000000
C	-4.194919000000	4.955395000000	8.220140000000
C	-5.238247000000	2.996927000000	9.170014000000
C	-5.146159000000	4.398654000000	9.094545000000
H	-4.167422000000	6.041324000000	8.082153000000
H	-6.024045000000	2.542434000000	9.782721000000

C	-6.057771000000	5.282460000000	9.912526000000
H	-5.588639000000	5.545680000000	10.877658000000
H	-6.279733000000	6.226681000000	9.390396000000
H	-7.011290000000	4.779963000000	10.139302000000
C	-4.489552000000	0.624633000000	8.516511000000
H	-4.197145000000	0.254125000000	7.507949000000
C	-2.318223000000	4.790823000000	6.471750000000
H	-2.249517000000	4.085732000000	5.619631000000
C	-5.930260000000	0.137595000000	8.734414000000
C	-6.286776000000	-0.745526000000	9.769691000000
C	-6.937057000000	0.551895000000	7.835198000000
C	-7.611327000000	-1.196021000000	9.906904000000
H	-5.527462000000	-1.090028000000	10.476925000000
C	-8.257440000000	0.104417000000	7.968138000000
H	-6.684367000000	1.252332000000	7.031362000000
C	-8.600946000000	-0.774872000000	9.008967000000
H	-7.864976000000	-1.880966000000	10.721752000000
H	-9.020634000000	0.443153000000	7.260466000000
H	-9.631397000000	-1.126003000000	9.116678000000
C	-3.477642000000	0.005140000000	9.482101000000
C	-2.688666000000	-1.085237000000	9.063310000000
C	-3.321531000000	0.487665000000	10.796762000000
C	-1.762479000000	-1.677321000000	9.937820000000
H	-2.798093000000	-1.470806000000	8.041940000000
C	-2.401882000000	-0.107256000000	11.674025000000
H	-3.924206000000	1.336480000000	11.136996000000
C	-1.616179000000	-1.190626000000	11.246401000000
H	-1.155926000000	-2.521870000000	9.595270000000
H	-2.299134000000	0.275875000000	12.694060000000
H	-0.898023000000	-1.653225000000	11.930076000000
C	-2.857969000000	6.104500000000	5.882016000000
C	-4.055602000000	6.062758000000	5.137829000000
C	-2.207285000000	7.342073000000	6.026874000000
C	-4.595906000000	7.221128000000	4.569142000000
H	-4.569006000000	5.104963000000	4.998153000000
C	-2.745432000000	8.508972000000	5.455504000000
H	-1.267449000000	7.407238000000	6.581817000000
C	-3.941942000000	8.454866000000	4.729009000000
H	-5.523749000000	7.160329000000	3.991871000000
H	-2.220380000000	9.460890000000	5.581296000000
H	-4.361686000000	9.363542000000	4.286829000000
C	-0.900544000000	4.905519000000	7.034145000000
C	0.189618000000	4.390155000000	6.304123000000
C	-0.646233000000	5.519688000000	8.277786000000
C	1.501613000000	4.481291000000	6.804578000000
H	0.017173000000	3.897623000000	5.341248000000
C	0.662420000000	5.629188000000	8.774070000000
H	-1.481872000000	5.912574000000	8.866578000000
C	1.741867000000	5.107732000000	8.040522000000
H	2.333809000000	4.041520000000	6.243265000000
H	0.838569000000	6.117208000000	9.737627000000
H	2.761774000000	5.182111000000	8.430293000000

C	0.758415000000	0.042142000000	6.870479000000
C	1.094418000000	-1.172525000000	6.208873000000
C	1.764187000000	0.693824000000	7.635863000000
C	2.364338000000	-1.719862000000	6.319240000000
H	0.327525000000	-1.669156000000	5.609765000000
C	3.043392000000	0.160886000000	7.735734000000
H	1.568795000000	1.656353000000	8.108055000000
C	3.351789000000	-1.049889000000	7.079165000000
H	2.609548000000	-2.656147000000	5.806132000000
H	3.830228000000	0.677527000000	8.290571000000
O	4.618139000000	-1.514976000000	7.210095000000
H	4.730266000000	-2.338276000000	6.695672000000
C	-4.999460000000	1.498086000000	4.343088000000
H	-4.167592000000	2.002287000000	4.957471000000
H	-5.886044000000	1.791852000000	4.934186000000
C	-4.964328000000	2.199499000000	2.973445000000
H	-4.726986000000	3.269118000000	3.082218000000
H	-4.132681000000	1.780331000000	2.379178000000
C	-4.898274000000	-0.055799000000	4.231832000000
H	-5.276299000000	-0.546110000000	5.146830000000
H	-5.561713000000	-0.396326000000	3.410036000000
C	-3.429908000000	-0.365613000000	3.989612000000
H	-3.056159000000	0.116594000000	3.065696000000
Si	-2.746662000000	-2.087291000000	4.111250000000
O	-1.222349000000	-1.943666000000	3.385854000000
O	-3.636438000000	-3.251282000000	3.302817000000
O	-2.684246000000	-2.627118000000	5.684429000000
C	-0.410723000000	-3.073615000000	3.015191000000
H	0.382526000000	-2.713453000000	2.342278000000
H	0.055292000000	-3.530369000000	3.907210000000
H	-1.006372000000	-3.842917000000	2.494716000000
C	-2.874404000000	-3.990348000000	6.106236000000
H	-3.609693000000	-4.509963000000	5.470904000000
H	-1.916266000000	-4.539199000000	6.077220000000
H	-3.240107000000	-3.969513000000	7.145182000000
C	-3.961286000000	-3.186228000000	1.907592000000
H	-4.363750000000	-4.167739000000	1.613110000000
H	-4.732298000000	-2.418071000000	1.715422000000
H	-3.074726000000	-2.961668000000	1.286980000000

MA

C	2.166035000000	-0.766375000000	0.000079000000
H	1.654376000000	-1.723253000000	0.000189000000
H	3.251211000000	-0.772619000000	0.000022000000
C	1.490195000000	0.381908000000	-0.000043000000
H	1.991260000000	1.344393000000	-0.000240000000
C	0.009538000000	0.482356000000	-0.000018000000
O	-0.605352000000	-0.711108000000	-0.000130000000
O	-0.586426000000	1.538624000000	0.000059000000
C	-2.033476000000	-0.671879000000	0.000053000000
H	-2.401232000000	-0.155073000000	0.889297000000
H	-2.401372000000	-0.156982000000	-0.890245000000

H	-2.353773000000	-1.712649000000	0.001125000000

I_{MA}

O	6.123309000000	1.414824000000	0.127882000000
Si	4.544969000000	1.950524000000	0.112489000000
O	3.801294000000	0.947374000000	1.099309000000
Si	3.301849000000	-0.466949000000	1.645880000000
O	3.342675000000	-1.733262000000	0.597877000000
O	3.791482000000	1.964046000000	-1.359404000000
O	4.411345000000	3.480143000000	0.745420000000
Si	4.690085000000	4.342445000000	2.112342000000
O	3.315260000000	4.802021000000	2.868387000000
Si	2.270984000000	6.086187000000	3.034345000000
O	0.770135000000	5.388539000000	2.853916000000
O	1.772569000000	-0.103984000000	2.190205000000
Si	0.883145000000	1.276227000000	2.567888000000
O	-0.719708000000	0.904440000000	2.554759000000
O	4.268982000000	-0.891594000000	2.956176000000
O	5.640759000000	5.629006000000	1.761383000000
Si	5.933047000000	6.760396000000	0.578630000000
O	5.151778000000	8.098800000000	1.266012000000
O	5.346669000000	6.399809000000	-0.905822000000
O	7.568451000000	6.938168000000	0.409974000000
O	5.604533000000	3.443076000000	3.171280000000
Si	5.396656000000	2.417787000000	4.465501000000
O	4.057360000000	1.460760000000	4.345106000000
O	1.004664000000	2.542743000000	1.499145000000
Si	0.176116000000	3.201595000000	0.214750000000
O	-1.345327000000	3.650369000000	0.637382000000
Si	-2.242909000000	4.945014000000	1.136661000000
O	-3.823553000000	4.499879000000	0.983711000000
O	0.045257000000	2.147692000000	-1.051283000000
O	1.087237000000	4.540070000000	-0.103040000000
Si	1.419148000000	5.619239000000	-1.319041000000
O	2.567025000000	6.669315000000	-0.729699000000
O	2.217175000000	4.783954000000	-2.531051000000
O	0.060358000000	6.319550000000	-1.922868000000
O	1.349079000000	1.858499000000	4.044277000000
O	6.805525000000	1.548980000000	4.532175000000
Si	7.816121000000	0.673533000000	3.543742000000
O	6.860166000000	-0.139332000000	2.417838000000
O	8.870570000000	1.622340000000	2.721621000000
Si	9.058817000000	3.091862000000	2.009787000000
O	10.648282000000	3.437154000000	1.739386000000
O	8.344837000000	2.953697000000	0.490465000000
O	8.407311000000	4.322951000000	2.909222000000
O	8.575611000000	-0.389259000000	4.539003000000
O	5.155309000000	3.277934000000	5.859823000000
O	-1.829695000000	6.192045000000	0.073317000000
O	-1.992137000000	5.392794000000	2.709276000000
O	2.531081000000	7.273454000000	1.902811000000
O	2.430718000000	6.805600000000	4.513406000000

H	6.876539000000	2.056193000000	0.045611000000
H	3.642494000000	2.842171000000	-1.766061000000
H	5.250251000000	-0.817475000000	2.753164000000
H	-1.079445000000	0.068019000000	2.917311000000
H	2.318088000000	1.790614000000	4.232980000000
H	-4.453820000000	4.878902000000	1.634798000000
H	-1.052507000000	5.609874000000	2.922309000000
H	2.768916000000	-1.694058000000	-0.186912000000
H	0.874677000000	4.452857000000	2.566969000000
H	2.315140000000	6.212225000000	5.282480000000
H	5.847356000000	3.928347000000	6.071491000000
H	4.187148000000	0.560742000000	3.932694000000
H	11.143900000000	3.853338000000	2.464716000000
H	8.592742000000	3.668168000000	-0.126385000000
H	7.438903000000	4.249716000000	3.054835000000
H	5.241947000000	8.933066000000	0.770675000000
H	8.076179000000	6.833381000000	1.234769000000
H	6.719847000000	0.338447000000	1.555392000000
H	9.284647000000	-0.927649000000	4.147777000000
H	-0.712631000000	6.298164000000	-1.302963000000
H	1.808809000000	4.856517000000	-3.410963000000
H	0.895299000000	1.906267000000	-1.463920000000
H	-2.250324000000	7.047816000000	0.269792000000
H	3.424694000000	7.709500000000	1.908497000000
H	4.358749000000	6.476744000000	-0.995037000000
H	2.421998000000	6.953968000000	0.212835000000
Ni	-3.404691000000	1.373935000000	6.293720000000
N	-2.370920000000	2.300709000000	7.697007000000
C	-6.151964000000	6.026610000000	2.567841000000
H	-5.711692000000	6.865323000000	3.119603000000
H	-6.500332000000	6.225608000000	1.549278000000
C	-6.293535000000	4.811533000000	3.135835000000
C	-5.909046000000	4.469302000000	4.549274000000
H	-5.525319000000	5.367281000000	5.061479000000
H	-6.823159000000	4.151415000000	5.089342000000
C	-0.792849000000	0.719174000000	7.003702000000
C	-1.186718000000	1.782365000000	7.982771000000
H	-6.745258000000	3.999135000000	2.547666000000
O	-1.739765000000	0.389559000000	6.209075000000
C	-0.374054000000	2.155745000000	9.182707000000
H	-0.056965000000	1.245988000000	9.718538000000
H	0.523379000000	2.722157000000	8.881118000000
H	-0.950089000000	2.784791000000	9.871464000000
C	-2.906618000000	3.298405000000	8.590644000000
C	-2.599759000000	4.669080000000	8.388149000000
C	-3.759894000000	2.875943000000	9.642614000000
C	-3.079389000000	5.589638000000	9.338551000000
C	-4.226555000000	3.843911000000	10.544995000000
C	-3.871428000000	5.201246000000	10.432638000000
H	-2.857889000000	6.652038000000	9.193915000000
H	-4.903228000000	3.529475000000	11.346899000000
C	-4.342535000000	6.211506000000	11.451412000000

H	-5.354247000000	5.973679000000	11.817158000000
H	-3.673979000000	6.224248000000	12.330891000000
H	-4.356041000000	7.230622000000	11.034666000000
C	-4.137328000000	1.396058000000	9.786726000000
H	-4.153318000000	0.987354000000	8.753399000000
C	-1.778712000000	5.128148000000	7.172481000000
H	-1.984850000000	4.400497000000	6.361726000000
C	-5.555862000000	1.191521000000	10.339395000000
C	-5.823418000000	0.427457000000	11.489621000000
C	-6.645530000000	1.760715000000	9.644203000000
C	-7.143095000000	0.241453000000	11.936897000000
H	-4.999722000000	-0.032405000000	12.042545000000
C	-7.961410000000	1.575524000000	10.087072000000
H	-6.456266000000	2.371176000000	8.754015000000
C	-8.215736000000	0.812874000000	11.239602000000
H	-7.327938000000	-0.355948000000	12.834830000000
H	-8.790254000000	2.027908000000	9.533656000000
H	-9.242243000000	0.666596000000	11.588216000000
C	-3.062500000000	0.598563000000	10.528892000000
C	-2.626224000000	-0.637663000000	10.012282000000
C	-2.498419000000	1.057314000000	11.736073000000
C	-1.646841000000	-1.394434000000	10.675247000000
H	-3.065362000000	-1.015834000000	9.081432000000
C	-1.523869000000	0.301792000000	12.405965000000
H	-2.821992000000	2.015544000000	12.155512000000
C	-1.090498000000	-0.924646000000	11.875440000000
H	-1.323243000000	-2.352539000000	10.256925000000
H	-1.102430000000	0.671951000000	13.345684000000
H	-0.329728000000	-1.512697000000	12.397241000000
C	-2.204407000000	6.503631000000	6.620390000000
C	-3.570548000000	6.845703000000	6.530073000000
C	-1.260264000000	7.418520000000	6.115915000000
C	-3.978357000000	8.062740000000	5.966611000000
H	-4.327856000000	6.160859000000	6.924444000000
C	-1.665469000000	8.636745000000	5.546184000000
H	-0.193016000000	7.186887000000	6.161036000000
C	-3.025342000000	8.966779000000	5.470766000000
H	-5.044515000000	8.308531000000	5.923121000000
H	-0.907800000000	9.328335000000	5.165166000000
H	-3.341013000000	9.920170000000	5.036632000000
C	-0.272944000000	5.037978000000	7.436648000000
C	0.561945000000	4.346614000000	6.536971000000
C	0.309744000000	5.649840000000	8.565354000000
C	1.947248000000	4.258142000000	6.766577000000
H	0.138300000000	3.864314000000	5.648707000000
C	1.691423000000	5.571164000000	8.794154000000
H	-0.323568000000	6.193182000000	9.273753000000
C	2.515482000000	4.870419000000	7.896544000000
H	2.584616000000	3.696534000000	6.077859000000
H	2.124591000000	6.055487000000	9.674716000000
H	3.592104000000	4.792887000000	8.075112000000
C	0.492150000000	0.064422000000	6.844651000000

C	0.575438000000	-0.923919000000	5.816760000000
C	1.684281000000	0.383244000000	7.557187000000
C	1.782047000000	-1.509419000000	5.475001000000
H	-0.334645000000	-1.197026000000	5.276638000000
C	2.895388000000	-0.212229000000	7.231451000000
H	1.684918000000	1.129925000000	8.348638000000
C	2.964329000000	-1.126886000000	6.155560000000
H	1.830185000000	-2.246639000000	4.667548000000
H	3.816778000000	0.061211000000	7.751225000000
O	4.181906000000	-1.596054000000	5.813315000000
H	4.149348000000	-1.971320000000	4.905026000000
C	-4.689695000000	2.879148000000	6.092189000000
H	-4.187143000000	3.651204000000	6.693833000000
H	-5.656243000000	2.653850000000	6.574149000000
C	-4.863758000000	3.316198000000	4.642984000000
H	-3.906525000000	3.666144000000	4.217535000000
H	-5.205255000000	2.485563000000	4.002342000000
C	-5.160008000000	0.343033000000	5.837971000000
H	-5.840895000000	0.888688000000	5.182833000000
H	-5.557537000000	0.052718000000	6.817140000000
C	-4.059510000000	-0.326987000000	5.308071000000
H	-3.548659000000	-1.099201000000	5.891764000000
C	-3.597072000000	-0.311721000000	3.886555000000
O	-4.370522000000	0.433078000000	3.076037000000
O	-2.598755000000	-0.941820000000	3.524121000000
C	-3.953162000000	0.516898000000	1.678786000000
H	-2.972104000000	1.008501000000	1.608891000000
H	-3.905702000000	-0.493506000000	1.246515000000
H	-4.725835000000	1.120997000000	1.188927000000

TS_{MA}

O	6.079332000000	1.793997000000	-0.448831000000
Si	4.544461000000	1.949709000000	0.112780000000
O	4.275678000000	0.764418000000	1.199447000000
Si	3.301855000000	-0.465402000000	1.645151000000
O	3.308421000000	-1.747532000000	0.611081000000
O	3.401937000000	1.768706000000	-1.090048000000
O	4.283316000000	3.424220000000	0.825561000000
Si	4.690372000000	4.342105000000	2.112659000000
O	3.373227000000	4.855592000000	2.929126000000
Si	2.270953000000	6.086134000000	3.034252000000
O	0.800170000000	5.340447000000	2.818580000000
O	1.777767000000	0.031559000000	1.941768000000
Si	0.883695000000	1.275818000000	2.568851000000
O	-0.706499000000	0.859719000000	2.581081000000
O	4.053842000000	-0.986050000000	3.070412000000
O	5.681485000000	5.574356000000	1.706495000000
Si	5.933058000000	6.760289000000	0.578648000000
O	5.157287000000	8.071423000000	1.324546000000
O	5.351240000000	6.438525000000	-0.912579000000
O	7.566281000000	6.973700000000	0.396173000000
O	5.607316000000	3.442735000000	3.174424000000

Si	5.396461000000	2.417763000000	4.465671000000
O	4.060911000000	1.449550000000	4.367468000000
O	0.912075000000	2.648193000000	1.616323000000
Si	0.175902000000	3.201542000000	0.213634000000
O	-1.371076000000	3.684103000000	0.503807000000
Si	-2.242870000000	4.945035000000	1.136723000000
O	-3.821311000000	4.499045000000	0.968413000000
O	0.156282000000	2.039051000000	-0.956345000000
O	1.114470000000	4.510667000000	-0.125537000000
Si	1.419175000000	5.619217000000	-1.318777000000
O	2.603062000000	6.631290000000	-0.727981000000
O	2.145608000000	4.841300000000	-2.603220000000
O	0.050345000000	6.373634000000	-1.831633000000
O	1.392346000000	1.719463000000	4.070424000000
O	6.802949000000	1.549065000000	4.515142000000
Si	7.816167000000	0.674161000000	3.543482000000
O	6.871876000000	-0.399468000000	2.643667000000
O	8.657908000000	1.555208000000	2.450064000000
Si	9.058799000000	3.091489000000	2.009902000000
O	10.683277000000	3.288702000000	1.818648000000
O	8.316008000000	3.232152000000	0.509968000000
O	8.513402000000	4.224120000000	3.094179000000
O	8.782975000000	-0.127388000000	4.607667000000
O	5.155429000000	3.269875000000	5.864489000000
O	-1.826555000000	6.278438000000	0.185959000000
O	-1.956368000000	5.267565000000	2.734320000000
O	2.532261000000	7.267066000000	1.894269000000
O	2.351698000000	6.835140000000	4.505973000000
H	6.815603000000	2.368373000000	-0.123607000000
H	3.422206000000	2.470864000000	-1.770917000000
H	5.039280000000	-1.015365000000	2.938221000000
H	-1.065346000000	0.007667000000	2.902426000000
H	2.369721000000	1.716024000000	4.237370000000
H	-4.473219000000	4.978931000000	1.523674000000
H	-1.013581000000	5.492028000000	2.932135000000
H	2.799886000000	-1.667565000000	-0.214335000000
H	0.920731000000	4.409182000000	2.506367000000
H	2.222675000000	6.247943000000	5.277532000000
H	5.831336000000	3.939146000000	6.069004000000
H	4.164102000000	0.533609000000	3.988845000000
H	11.175014000000	3.653679000000	2.573870000000
H	8.514285000000	4.053756000000	0.021719000000
H	7.535440000000	4.231957000000	3.178471000000
H	5.233085000000	8.916003000000	0.843654000000
H	8.065640000000	7.069321000000	1.226271000000
H	6.895850000000	-0.212792000000	1.684509000000
H	9.471708000000	-0.700352000000	4.229751000000
H	-0.701298000000	6.372270000000	-1.186738000000
H	1.665854000000	4.925869000000	-3.445098000000
H	1.057678000000	1.726205000000	-1.178110000000
H	-2.059881000000	7.143478000000	0.566427000000
H	3.426518000000	7.701840000000	1.910610000000

H	4.358953000000	6.476594000000	-0.999799000000
H	2.454480000000	6.922835000000	0.212092000000
Ni	-3.529119000000	1.349949000000	5.936740000000
N	-2.601172000000	2.370479000000	7.387603000000
C	-6.142592000000	6.278679000000	2.424406000000
H	-5.718749000000	7.024433000000	3.106415000000
H	-6.361988000000	6.603658000000	1.402259000000
C	-6.411480000000	5.023102000000	2.835152000000
C	-6.201353000000	4.495596000000	4.229797000000
H	-5.910500000000	5.317320000000	4.905600000000
H	-7.173301000000	4.107621000000	4.595894000000
C	-1.001422000000	0.786216000000	6.790675000000
C	-1.437298000000	1.857805000000	7.742664000000
H	-6.835422000000	4.308353000000	2.114455000000
O	-1.891639000000	0.471451000000	5.920256000000
C	-0.688392000000	2.231586000000	8.983047000000
H	-0.392374000000	1.323692000000	9.533668000000
H	0.218999000000	2.808390000000	8.736834000000
H	-1.308846000000	2.853683000000	9.639903000000
C	-3.196260000000	3.383909000000	8.219323000000
C	-2.882233000000	4.751683000000	8.006463000000
C	-4.121171000000	2.982578000000	9.219549000000
C	-3.426121000000	5.690439000000	8.902086000000
C	-4.646772000000	3.968536000000	10.069925000000
C	-4.285898000000	5.323110000000	9.952278000000
H	-3.196987000000	6.750251000000	8.750213000000
H	-5.373205000000	3.669874000000	10.833226000000
C	-4.818453000000	6.354724000000	10.918286000000
H	-5.825867000000	6.090317000000	11.276955000000
H	-4.166699000000	6.434298000000	11.806865000000
H	-4.866224000000	7.353978000000	10.457759000000
C	-4.507355000000	1.504684000000	9.365343000000
H	-4.459738000000	1.076624000000	8.338979000000
C	-1.978387000000	5.186274000000	6.840253000000
H	-2.146551000000	4.456379000000	6.022993000000
C	-5.955260000000	1.299522000000	9.838275000000
C	-6.286774000000	0.465445000000	10.921650000000
C	-7.005971000000	1.925918000000	9.132126000000
C	-7.628027000000	0.265892000000	11.291860000000
H	-5.495421000000	-0.037552000000	11.483926000000
C	-8.343635000000	1.728259000000	9.498406000000
H	-6.770088000000	2.596723000000	8.298733000000
C	-8.660919000000	0.893886000000	10.583424000000
H	-7.860871000000	-0.385968000000	12.139305000000
H	-9.140301000000	2.228611000000	8.938944000000
H	-9.704314000000	0.738107000000	10.872633000000
C	-3.477252000000	0.719952000000	10.181404000000
C	-2.998998000000	-0.516208000000	9.703859000000
C	-3.000596000000	1.190818000000	11.421002000000
C	-2.062718000000	-1.262071000000	10.437874000000
H	-3.370904000000	-0.902681000000	8.747440000000
C	-2.069294000000	0.446408000000	12.160995000000

H	-3.359783000000	2.148822000000	11.811064000000
C	-1.593074000000	-0.780717000000	11.670015000000
H	-1.705208000000	-2.220648000000	10.049273000000
H	-1.715453000000	0.825474000000	13.124729000000
H	-0.866274000000	-1.360149000000	12.246968000000
C	-2.345762000000	6.564838000000	6.256625000000
C	-3.697875000000	6.912178000000	6.053185000000
C	-1.360046000000	7.480721000000	5.842425000000
C	-4.054013000000	8.137892000000	5.474665000000
H	-4.485261000000	6.223160000000	6.374618000000
C	-1.712438000000	8.706532000000	5.253451000000
H	-0.300933000000	7.244802000000	5.973112000000
C	-3.060146000000	9.044097000000	5.070504000000
H	-5.112102000000	8.390989000000	5.349348000000
H	-0.923128000000	9.398835000000	4.944727000000
H	-3.335731000000	10.004915000000	4.625431000000
C	-0.493528000000	5.077248000000	7.199946000000
C	0.387123000000	4.365745000000	6.362067000000
C	0.026247000000	5.695218000000	8.355983000000
C	1.755285000000	4.266025000000	6.675786000000
H	0.011410000000	3.878092000000	5.455919000000
C	1.390644000000	5.605366000000	8.669246000000
H	-0.643240000000	6.252927000000	9.018649000000
C	2.261015000000	4.886858000000	7.830726000000
H	2.424927000000	3.690806000000	6.029824000000
H	1.774691000000	6.095323000000	9.569255000000
H	3.324478000000	4.803247000000	8.074394000000
C	0.267982000000	0.090720000000	6.727434000000
C	0.367504000000	-0.976853000000	5.784160000000
C	1.440413000000	0.455201000000	7.448698000000
C	1.576899000000	-1.600344000000	5.529771000000
H	-0.530905000000	-1.287135000000	5.244253000000
C	2.654596000000	-0.173844000000	7.207250000000
H	1.425646000000	1.271092000000	8.168865000000
C	2.742260000000	-1.174844000000	6.214134000000
H	1.641404000000	-2.405995000000	4.791198000000
H	3.564153000000	0.135440000000	7.727748000000
O	3.963111000000	-1.688905000000	5.952677000000
H	3.940814000000	-2.153116000000	5.088281000000
C	-5.239437000000	2.595005000000	5.627030000000
H	-4.713899000000	3.117829000000	6.446007000000
H	-6.278081000000	2.531420000000	5.985730000000
C	-5.160068000000	3.344729000000	4.293309000000
H	-4.148642000000	3.753069000000	4.123409000000
H	-5.346984000000	2.653383000000	3.454384000000
C	-5.448953000000	0.676636000000	5.282541000000
H	-6.092037000000	0.950933000000	4.439321000000
H	-6.014470000000	0.338942000000	6.159852000000
C	-4.291317000000	-0.121490000000	4.945984000000
H	-4.019126000000	-0.976678000000	5.577715000000
C	-3.702947000000	-0.223610000000	3.581156000000
O	-4.319556000000	0.572283000000	2.679611000000

O	-2.745184000000	-0.960684000000	3.322640000000
C	-3.785513000000	0.555698000000	1.322251000000
H	-2.745254000000	0.910151000000	1.324646000000
H	-3.841266000000	-0.464998000000	0.915108000000
H	-4.426066000000	1.244051000000	0.758098000000

P_{MA}

O	6.124557000000	1.493285000000	0.381839000000
Si	4.545472000000	1.950756000000	0.111925000000
O	3.707880000000	0.596329000000	0.487246000000
Si	3.300801000000	-0.466363000000	1.646544000000
O	3.079789000000	-1.971098000000	0.997459000000
O	4.292485000000	2.325079000000	-1.470390000000
O	4.088631000000	3.253164000000	1.035688000000
Si	4.690504000000	4.343066000000	2.111807000000
O	3.487102000000	4.981149000000	2.989380000000
Si	2.270870000000	6.086179000000	3.034422000000
O	0.847719000000	5.227295000000	2.988143000000
O	1.958901000000	0.027181000000	2.458396000000
Si	0.883519000000	1.274979000000	2.568144000000
O	-0.619490000000	0.720806000000	2.943336000000
O	4.428401000000	-0.670292000000	2.874980000000
O	5.729919000000	5.389538000000	1.460060000000
Si	5.932847000000	6.760114000000	0.578830000000
O	5.063847000000	7.917580000000	1.467676000000
O	5.385423000000	6.642348000000	-0.958765000000
O	7.549756000000	7.088216000000	0.510914000000
O	5.656012000000	3.492788000000	3.199893000000
Si	5.396517000000	2.417514000000	4.465788000000
O	3.988220000000	1.575543000000	4.275951000000
O	0.958300000000	2.102505000000	1.143400000000
Si	0.176248000000	3.201578000000	0.214526000000
O	-1.215235000000	3.647403000000	0.994201000000
Si	-2.242902000000	4.945130000000	1.136731000000
O	-3.823330000000	4.483654000000	1.323862000000
O	-0.253297000000	2.487745000000	-1.221990000000
O	1.126922000000	4.534294000000	-0.074469000000
Si	1.419131000000	5.619505000000	-1.319239000000
O	2.606636000000	6.665122000000	-0.816004000000
O	2.098300000000	4.648206000000	-2.509998000000
O	0.065973000000	6.396414000000	-1.820655000000
O	1.321093000000	2.330573000000	3.785974000000
O	6.750299000000	1.476904000000	4.519609000000
Si	7.816207000000	0.673544000000	3.543753000000
O	6.946419000000	-0.142035000000	2.355343000000
O	8.883427000000	1.666558000000	2.800500000000
Si	9.058812000000	3.091856000000	2.009741000000
O	10.645170000000	3.457667000000	1.743292000000
O	8.401011000000	2.855388000000	0.476391000000
O	8.362058000000	4.350132000000	2.829278000000
O	8.566353000000	-0.398583000000	4.537502000000
O	5.187635000000	3.231849000000	5.888588000000

O	-2.174785000000	5.725310000000	-0.356783000000
O	-1.800935000000	5.877015000000	2.421075000000
O	2.393486000000	7.191870000000	1.799017000000
O	2.404184000000	6.996702000000	4.412245000000
H	6.884569000000	2.118356000000	0.219891000000
H	3.730436000000	3.095749000000	-1.687534000000
H	5.416955000000	-0.606805000000	2.644277000000
H	-1.044586000000	0.057018000000	2.346831000000
H	2.261004000000	2.185337000000	4.070824000000
H	-4.180588000000	4.572461000000	2.230573000000
H	-0.860034000000	5.756545000000	2.703487000000
H	2.691973000000	-2.011986000000	0.106288000000
H	0.959639000000	4.273626000000	3.228937000000
H	2.351587000000	6.522739000000	5.259633000000
H	5.936468000000	3.771250000000	6.194480000000
H	4.109998000000	0.675204000000	3.857295000000
H	11.122118000000	3.917097000000	2.454911000000
H	8.740792000000	3.461119000000	-0.207792000000
H	7.406486000000	4.216685000000	3.033974000000
H	5.301756000000	8.841196000000	1.267535000000
H	8.061187000000	6.752286000000	1.269971000000
H	6.755231000000	0.404987000000	1.534720000000
H	9.297984000000	-0.913014000000	4.156069000000
H	-0.774233000000	6.164766000000	-1.345414000000
H	2.103791000000	5.000162000000	-3.417244000000
H	0.357786000000	2.651521000000	-1.963286000000
H	-2.951152000000	6.278736000000	-0.555226000000
H	3.268088000000	7.667644000000	1.837367000000
H	4.396376000000	6.664087000000	-1.053684000000
H	2.428413000000	6.973626000000	0.116445000000
Ni	-3.944600000000	-1.701001000000	4.461794000000
N	-3.742588000000	-1.105428000000	6.262074000000
C	-8.644880000000	-0.909505000000	-1.983040000000
H	-8.278090000000	0.121362000000	-2.049314000000
H	-8.976830000000	-1.376343000000	-2.914809000000
C	-8.693638000000	-1.564273000000	-0.810072000000
C	-8.256471000000	-0.997629000000	0.515050000000
H	-7.961492000000	0.060464000000	0.389156000000
H	-9.111877000000	-1.009186000000	1.220249000000
C	-1.564184000000	-1.698420000000	5.715878000000
C	-2.514691000000	-1.145235000000	6.742875000000
H	-9.071688000000	-2.596675000000	-0.790661000000
O	-2.130659000000	-2.009527000000	4.599917000000
C	-2.144066000000	-0.695275000000	8.117769000000
H	-1.719859000000	-1.536553000000	8.692953000000
H	-1.404338000000	0.121529000000	8.075884000000
H	-3.029827000000	-0.329444000000	8.652398000000
C	-4.841725000000	-0.529208000000	6.989489000000
C	-5.119850000000	0.850555000000	6.818348000000
C	-5.691493000000	-1.373063000000	7.745243000000
C	-6.226494000000	1.383581000000	7.500463000000
C	-6.786144000000	-0.786151000000	8.400910000000

C	-7.058160000000	0.591850000000	8.313884000000
H	-6.463383000000	2.444380000000	7.365093000000
H	-7.460509000000	-1.431840000000	8.973477000000
C	-8.217959000000	1.202076000000	9.064579000000
H	-9.039052000000	0.479675000000	9.195531000000
H	-7.906002000000	1.528455000000	10.072996000000
H	-8.615680000000	2.087499000000	8.543974000000
C	-5.412278000000	-2.879826000000	7.815297000000
H	-4.987051000000	-3.156220000000	6.828179000000
C	-4.232364000000	1.710943000000	5.909835000000
H	-3.862487000000	1.034594000000	5.110126000000
C	-6.696417000000	-3.707479000000	7.962670000000
C	-7.020963000000	-4.418246000000	9.131792000000
C	-7.594116000000	-3.758711000000	6.875211000000
C	-8.215372000000	-5.154741000000	9.214459000000
H	-6.334931000000	-4.408180000000	9.983617000000
C	-8.784741000000	-4.492039000000	6.953477000000
H	-7.357151000000	-3.207613000000	5.956943000000
C	-9.101018000000	-5.193881000000	8.129023000000
H	-8.448080000000	-5.702814000000	10.132513000000
H	-9.465882000000	-4.520101000000	6.097263000000
H	-10.028422000000	-5.770442000000	8.193416000000
C	-4.333328000000	-3.228347000000	8.842720000000
C	-3.357395000000	-4.194171000000	8.525105000000
C	-4.293583000000	-2.625588000000	10.115617000000
C	-2.361891000000	-4.545584000000	9.449968000000
H	-3.382730000000	-4.681779000000	7.543492000000
C	-3.303574000000	-2.978979000000	11.046204000000
H	-5.039460000000	-1.868485000000	10.379656000000
C	-2.331279000000	-3.936721000000	10.715249000000
H	-1.613844000000	-5.298887000000	9.184027000000
H	-3.292248000000	-2.504212000000	12.032236000000
H	-1.559273000000	-4.211430000000	11.440186000000
C	-4.999854000000	2.819452000000	5.171813000000
C	-6.163280000000	2.488795000000	4.442453000000
C	-4.544955000000	4.150037000000	5.131847000000
C	-6.855290000000	3.456416000000	3.703220000000
H	-6.536577000000	1.458962000000	4.462801000000
C	-5.235859000000	5.126098000000	4.389108000000
H	-3.641578000000	4.434234000000	5.678066000000
C	-6.394325000000	4.784490000000	3.674085000000
H	-7.756565000000	3.176486000000	3.148969000000
H	-4.865360000000	6.156092000000	4.380744000000
H	-6.934364000000	5.543644000000	3.100523000000
C	-2.982245000000	2.211888000000	6.638141000000
C	-1.731790000000	2.163285000000	5.989974000000
C	-3.048651000000	2.736741000000	7.944354000000
C	-0.570580000000	2.616821000000	6.637492000000
H	-1.653555000000	1.765103000000	4.971156000000
C	-1.890578000000	3.193407000000	8.592156000000
H	-4.011336000000	2.781779000000	8.463824000000
C	-0.646199000000	3.129269000000	7.942452000000

H	0.384076000000	2.566447000000	6.105717000000
H	-1.960701000000	3.598771000000	9.606411000000
H	0.257468000000	3.480596000000	8.450304000000
C	-0.130630000000	-1.864764000000	5.794755000000
C	0.515735000000	-2.329115000000	4.606260000000
C	0.691708000000	-1.534885000000	6.913640000000
C	1.892070000000	-2.379203000000	4.506533000000
H	-0.099719000000	-2.613334000000	3.750592000000
C	2.076091000000	-1.577725000000	6.818489000000
H	0.253698000000	-1.214328000000	7.857411000000
C	2.689182000000	-1.937071000000	5.592724000000
H	2.365495000000	-2.717678000000	3.579958000000
H	2.709859000000	-1.283327000000	7.658572000000
O	4.026592000000	-1.832887000000	5.501304000000
H	4.289269000000	-1.823331000000	4.548179000000
C	-6.677489000000	-1.232620000000	2.512790000000
H	-6.358935000000	-0.184582000000	2.415964000000
H	-7.544165000000	-1.258869000000	3.199285000000
C	-7.089272000000	-1.789489000000	1.140671000000
H	-6.222745000000	-1.760498000000	0.455995000000
H	-7.378443000000	-2.853646000000	1.239183000000
C	-5.544451000000	-2.079968000000	3.134090000000
H	-5.907720000000	-3.102218000000	3.338158000000
H	-5.533899000000	-1.596714000000	4.269060000000
C	-4.162107000000	-2.141740000000	2.599994000000
H	-3.720260000000	-3.129344000000	2.421264000000
C	-3.432556000000	-1.060783000000	1.885463000000
O	-4.174447000000	0.048444000000	1.664292000000
O	-2.239801000000	-1.163524000000	1.565112000000
C	-3.505774000000	1.146247000000	0.976717000000
H	-2.716211000000	1.569211000000	1.612724000000
H	-3.071964000000	0.790927000000	0.030068000000
H	-4.284784000000	1.896730000000	0.801084000000

vCl

C	0.000000000000	0.761117000000	0.000000000000
H	-0.781650000000	1.511697000000	0.000000000000
C	1.295653000000	1.041451000000	0.000000000000
H	2.055691000000	0.267922000000	0.000000000000
H	1.612775000000	2.078715000000	0.000000000000
Cl	-0.627102000000	-0.863161000000	0.000000000000

I_{vCl}

O	6.063255000000	1.925598000000	-0.503107000000
Si	4.544919000000	1.950539000000	0.112427000000
O	4.366794000000	0.660386000000	1.104795000000
Si	3.301774000000	-0.466946000000	1.645913000000
O	3.282562000000	-1.831961000000	0.722740000000
O	3.387386000000	1.784879000000	-1.083137000000
O	4.199147000000	3.371512000000	0.896320000000
Si	4.690179000000	4.342484000000	2.112313000000
O	3.405819000000	4.892305000000	2.954433000000

Si	2.270961000000	6.086177000000	3.034359000000
O	0.814618000000	5.298095000000	2.839950000000
O	1.806493000000	0.150437000000	1.805922000000
Si	0.883327000000	1.276233000000	2.567970000000
O	-0.636204000000	0.637676000000	2.689245000000
O	3.940930000000	-0.906636000000	3.141632000000
O	5.702703000000	5.527890000000	1.650661000000
Si	5.933023000000	6.760385000000	0.578630000000
O	5.139095000000	8.036561000000	1.368246000000
O	5.353539000000	6.485594000000	-0.923094000000
O	7.560841000000	7.010810000000	0.406854000000
O	5.615618000000	3.450611000000	3.177276000000
Si	5.396706000000	2.417633000000	4.465578000000
O	4.045543000000	1.473492000000	4.374573000000
O	0.830102000000	2.676268000000	1.656387000000
Si	0.176008000000	3.201554000000	0.214648000000
O	-1.388506000000	3.693369000000	0.459738000000
Si	-2.242883000000	4.945013000000	1.136629000000
O	-3.859706000000	4.550913000000	1.169750000000
O	0.188723000000	2.029117000000	-0.939473000000
O	1.103473000000	4.522400000000	-0.112007000000
Si	1.419171000000	5.619257000000	-1.319007000000
O	2.595355000000	6.638041000000	-0.735340000000
O	2.144522000000	4.823969000000	-2.591442000000
O	0.045420000000	6.363043000000	-1.840489000000
O	1.401142000000	1.712874000000	4.067132000000
O	6.796618000000	1.543396000000	4.515664000000
Si	7.816014000000	0.673785000000	3.543645000000
O	6.892283000000	-0.454524000000	2.701813000000
O	8.599998000000	1.561458000000	2.411726000000
Si	9.058829000000	3.091742000000	2.009869000000
O	10.690715000000	3.258424000000	1.854180000000
O	8.348322000000	3.262601000000	0.497592000000
O	8.515176000000	4.221074000000	3.099110000000
O	8.831797000000	-0.062723000000	4.610814000000
O	5.165786000000	3.272889000000	5.865132000000
O	-1.946018000000	6.254297000000	0.118381000000
O	-1.897639000000	5.261872000000	2.714034000000
O	2.519940000000	7.247336000000	1.870946000000
O	2.316916000000	6.872047000000	4.484135000000
H	6.812718000000	2.449823000000	-0.127997000000
H	3.397568000000	2.505518000000	-1.745124000000
H	4.908146000000	-1.098046000000	3.068211000000
H	-1.037731000000	0.694700000000	3.585828000000
H	2.378827000000	1.742599000000	4.244513000000
H	-4.264996000000	4.411227000000	0.295901000000
H	-0.936108000000	5.446986000000	2.884253000000
H	2.917749000000	-1.764626000000	-0.176533000000
H	0.941911000000	4.370142000000	2.514865000000
H	2.056979000000	6.352572000000	5.269120000000
H	5.829865000000	3.956522000000	6.059775000000
H	4.108889000000	0.546561000000	3.996529000000

H	11.172467000000	3.604923000000	2.624495000000
H	8.580142000000	4.074960000000	0.009738000000
H	7.537394000000	4.215474000000	3.191210000000
H	5.239121000000	8.904860000000	0.936340000000
H	8.061607000000	7.065109000000	1.239901000000
H	6.848359000000	-0.276171000000	1.742572000000
H	9.501747000000	-0.659325000000	4.235774000000
H	-0.712856000000	6.363404000000	-1.207719000000
H	1.669076000000	4.891555000000	-3.437196000000
H	1.094963000000	1.706258000000	-1.131481000000
H	-2.258479000000	7.116398000000	0.447241000000
H	3.415102000000	7.684042000000	1.888100000000
H	4.362531000000	6.517527000000	-1.013040000000
H	2.450236000000	6.921174000000	0.209791000000
Ni	-3.436999000000	2.373684000000	5.002275000000
N	-2.701364000000	2.839806000000	6.793597000000
C	-7.872130000000	6.379504000000	2.197299000000
H	-7.061193000000	6.876222000000	1.651997000000
H	-8.875161000000	6.452290000000	1.767051000000
C	-7.643503000000	5.723194000000	3.348229000000
C	-6.297280000000	5.578304000000	4.006108000000
H	-5.540345000000	6.158114000000	3.448648000000
H	-6.337524000000	5.992688000000	5.032783000000
C	-1.279271000000	1.059277000000	6.220528000000
C	-1.708125000000	2.087900000000	7.226558000000
H	-8.486956000000	5.239993000000	3.862555000000
O	-1.990076000000	1.027709000000	5.155942000000
C	-1.129570000000	2.160171000000	8.603930000000
H	-0.996713000000	1.146395000000	9.013220000000
H	-0.148394000000	2.664476000000	8.592891000000
H	-1.785043000000	2.733437000000	9.271110000000
C	-3.257358000000	3.833381000000	7.679428000000
C	-2.712698000000	5.142684000000	7.707145000000
C	-4.401549000000	3.491931000000	8.446828000000
C	-3.289251000000	6.073583000000	8.588786000000
C	-4.929981000000	4.462522000000	9.313763000000
C	-4.375876000000	5.750743000000	9.420485000000
H	-2.895205000000	7.095424000000	8.596349000000
H	-5.821020000000	4.210787000000	9.898573000000
C	-4.942978000000	6.762551000000	10.387563000000
H	-4.459986000000	6.674686000000	11.377376000000
H	-4.780729000000	7.793215000000	10.034924000000
H	-6.023971000000	6.614536000000	10.538789000000
C	-5.028869000000	2.097196000000	8.322549000000
H	-4.888682000000	1.793932000000	7.262816000000
C	-1.553291000000	5.544531000000	6.780839000000
H	-1.672982000000	4.952996000000	5.852635000000
C	-6.546770000000	2.103257000000	8.558673000000
C	-7.169334000000	1.329133000000	9.554176000000
C	-7.360315000000	2.896977000000	7.721155000000
C	-8.566022000000	1.352183000000	9.713118000000
H	-6.565568000000	0.696877000000	10.210911000000

C	-8.751837000000	2.921337000000	7.876362000000
H	-6.892068000000	3.513451000000	6.945877000000
C	-9.362096000000	2.146444000000	8.877408000000
H	-9.029106000000	0.741728000000	10.494313000000
H	-9.362469000000	3.545388000000	7.216213000000
H	-10.448914000000	2.162327000000	9.000943000000
C	-4.281222000000	1.055470000000	9.157997000000
C	-3.946903000000	-0.189878000000	8.589763000000
C	-3.931217000000	1.297711000000	10.501484000000
C	-3.273020000000	-1.167065000000	9.340419000000
H	-4.228535000000	-0.398712000000	7.551195000000
C	-3.263041000000	0.321480000000	11.256419000000
H	-4.183069000000	2.259025000000	10.961711000000
C	-2.926757000000	-0.913353000000	10.676866000000
H	-3.025667000000	-2.129825000000	8.882117000000
H	-3.006198000000	0.525113000000	12.300625000000
H	-2.405907000000	-1.674285000000	11.265695000000
C	-1.635434000000	7.020957000000	6.352101000000
C	-2.732690000000	7.436364000000	5.567748000000
C	-0.662570000000	7.976624000000	6.696598000000
C	-2.857825000000	8.766482000000	5.148589000000
H	-3.499620000000	6.707846000000	5.286494000000
C	-0.784343000000	9.312239000000	6.275230000000
H	0.204349000000	7.685617000000	7.296409000000
C	-1.881643000000	9.713423000000	5.502296000000
H	-3.720468000000	9.065476000000	4.544503000000
H	-0.012373000000	10.036460000000	6.552372000000
H	-1.976811000000	10.753147000000	5.175354000000
C	-0.188715000000	5.162550000000	7.356320000000
C	0.765187000000	4.523595000000	6.536341000000
C	0.157237000000	5.446708000000	8.692814000000
C	2.034763000000	4.181033000000	7.036198000000
H	0.508754000000	4.273815000000	5.499912000000
C	1.423558000000	5.108244000000	9.196205000000
H	-0.573656000000	5.930115000000	9.349105000000
C	2.366400000000	4.475069000000	8.369650000000
H	2.769756000000	3.685761000000	6.394234000000
H	1.672969000000	5.340332000000	10.236303000000
H	3.353786000000	4.208644000000	8.758731000000
C	-0.200734000000	0.100795000000	6.379776000000
C	-0.271789000000	-1.101782000000	5.618882000000
C	0.966451000000	0.334861000000	7.159838000000
C	0.762747000000	-2.025873000000	5.637955000000
H	-1.168514000000	-1.305030000000	5.027604000000
C	2.014833000000	-0.574809000000	7.167190000000
H	1.092472000000	1.270153000000	7.705384000000
C	1.923923000000	-1.759357000000	6.402074000000
H	0.685036000000	-2.952590000000	5.059327000000
H	2.930875000000	-0.377594000000	7.729329000000
O	2.982320000000	-2.599607000000	6.447101000000
H	2.846237000000	-3.351169000000	5.836959000000
C	-4.517440000000	3.972616000000	4.820818000000

H	-3.709442000000	4.536633000000	4.304009000000
H	-4.618010000000	4.324812000000	5.861222000000
C	-5.840972000000	4.092436000000	4.085513000000
H	-5.786086000000	3.714539000000	3.051239000000
H	-6.632874000000	3.519043000000	4.604920000000
C	-3.843171000000	1.736460000000	3.132708000000
H	-4.122542000000	2.614879000000	2.540369000000
C	-4.759369000000	1.186131000000	4.032255000000
H	-5.760564000000	1.591766000000	4.196085000000
H	-2.995601000000	1.150061000000	2.762485000000
Cl	-4.670765000000	-0.488036000000	4.563104000000

TS$_{vCl}$

O	6.071282000000	1.883146000000	-0.481038000000
Si	4.544930000000	1.950713000000	0.112599000000
O	4.333486000000	0.692248000000	1.129922000000
Si	3.301850000000	-0.467190000000	1.645920000000
O	3.289814000000	-1.805295000000	0.683832000000
O	3.394536000000	1.779625000000	-1.088194000000
O	4.218792000000	3.385580000000	0.877068000000
Si	4.690174000000	4.342581000000	2.112064000000
O	3.405992000000	4.885025000000	2.959035000000
Si	2.270988000000	6.086150000000	3.034368000000
O	0.820199000000	5.316265000000	2.802376000000
O	1.789291000000	0.098586000000	1.857330000000
Si	0.883220000000	1.276280000000	2.567912000000
O	-0.687660000000	0.738935000000	2.625467000000
O	3.984487000000	-0.925781000000	3.116829000000
O	5.701387000000	5.536116000000	1.662660000000
Si	5.933007000000	6.760345000000	0.578675000000
O	5.164808000000	8.049366000000	1.369642000000
O	5.340121000000	6.479076000000	-0.915888000000
O	7.563633000000	6.988328000000	0.389746000000
O	5.617337000000	3.447525000000	3.176266000000
Si	5.396696000000	2.417635000000	4.465592000000
O	4.052015000000	1.464774000000	4.358642000000
O	0.829572000000	2.676213000000	1.671382000000
Si	0.176051000000	3.201554000000	0.214734000000
O	-1.381669000000	3.690991000000	0.440706000000
Si	-2.242915000000	4.945041000000	1.136648000000
O	-3.851455000000	4.589707000000	1.082539000000
O	0.198785000000	2.000700000000	-0.914551000000
O	1.134528000000	4.493879000000	-0.127528000000
Si	1.419167000000	5.619276000000	-1.319063000000
O	2.596104000000	6.635725000000	-0.727602000000
O	2.140899000000	4.846115000000	-2.608100000000
O	0.037618000000	6.356487000000	-1.818188000000
O	1.388279000000	1.663986000000	4.083202000000
O	6.799187000000	1.542501000000	4.518652000000
Si	7.816026000000	0.673659000000	3.543689000000
O	6.882878000000	-0.435394000000	2.682125000000
O	8.623639000000	1.556710000000	2.425143000000

Si	9.058834000000	3.091817000000	2.009837000000
O	10.688942000000	3.266511000000	1.844016000000
O	8.334385000000	3.252962000000	0.503279000000
O	8.515976000000	4.219903000000	3.099733000000
O	8.815331000000	-0.092008000000	4.607110000000
O	5.153910000000	3.264765000000	5.868506000000
O	-1.864077000000	6.283122000000	0.181236000000
O	-1.884507000000	5.214489000000	2.719115000000
O	2.544468000000	7.253362000000	1.881996000000
O	2.303900000000	6.857992000000	4.492947000000
H	6.814761000000	2.427778000000	-0.122709000000
H	3.405093000000	2.496143000000	-1.754548000000
H	4.960334000000	-1.059964000000	3.013499000000
H	-0.819489000000	-0.171900000000	2.946278000000
H	2.367235000000	1.703399000000	4.251599000000
H	-4.095294000000	3.757929000000	0.618880000000
H	-0.942495000000	5.467282000000	2.884934000000
H	2.911485000000	-1.718682000000	-0.208152000000
H	0.937687000000	4.382223000000	2.496685000000
H	2.024964000000	6.323437000000	5.262654000000
H	5.819979000000	3.944464000000	6.070980000000
H	4.127837000000	0.541625000000	3.975920000000
H	11.167990000000	3.647149000000	2.599752000000
H	8.543188000000	4.076151000000	0.022455000000
H	7.537544000000	4.224099000000	3.185057000000
H	5.251398000000	8.910506000000	0.920829000000
H	8.066147000000	7.080915000000	1.218265000000
H	6.864978000000	-0.254506000000	1.722265000000
H	9.530491000000	-0.626689000000	4.221995000000
H	-0.706114000000	6.378199000000	-1.163737000000
H	1.672085000000	4.947929000000	-3.454128000000
H	1.110885000000	1.707245000000	-1.124204000000
H	-2.263204000000	7.123971000000	0.467117000000
H	3.438530000000	7.689160000000	1.905267000000
H	4.347128000000	6.510906000000	-0.999086000000
H	2.451957000000	6.922138000000	0.216553000000
Ni	-2.909496000000	0.301742000000	5.587715000000
N	-2.305296000000	1.641845000000	6.985584000000
C	-4.849979000000	1.845544000000	-0.291040000000
H	-5.937039000000	1.971275000000	-0.223876000000
H	-4.384391000000	2.054686000000	-1.259213000000
C	-4.120270000000	1.413751000000	0.757133000000
C	-4.679719000000	1.029618000000	2.102296000000
H	-5.764439000000	1.245040000000	2.136400000000
H	-4.563803000000	-0.068828000000	2.183638000000
C	-0.436734000000	0.265977000000	6.758791000000
C	-1.131369000000	1.351835000000	7.512622000000
H	-3.034163000000	1.295124000000	0.639897000000
O	-1.180188000000	-0.361399000000	5.924487000000
C	-0.606806000000	1.903961000000	8.802277000000
H	-0.106838000000	1.112901000000	9.382405000000
H	0.117143000000	2.716950000000	8.623158000000

H	-1.425808000000	2.324001000000	9.401005000000
C	-3.158672000000	2.544547000000	7.718302000000
C	-3.113190000000	3.946694000000	7.522139000000
C	-4.049075000000	1.952004000000	8.647376000000
C	-3.905881000000	4.749536000000	8.361115000000
C	-4.798778000000	2.807569000000	9.474978000000
C	-4.722933000000	4.207505000000	9.368547000000
H	-3.892754000000	5.833728000000	8.207781000000
H	-5.504447000000	2.364058000000	10.185065000000
C	-5.516377000000	5.102756000000	10.290603000000
H	-4.923892000000	5.373243000000	11.183121000000
H	-5.802602000000	6.042483000000	9.792409000000
H	-6.433345000000	4.606496000000	10.645599000000
C	-4.244908000000	0.442319000000	8.709130000000
H	-4.025444000000	0.064475000000	7.678221000000
C	-2.216898000000	4.574446000000	6.441547000000
H	-2.183029000000	3.859712000000	5.596040000000
C	-5.711827000000	0.086050000000	8.965691000000
C	-6.112448000000	-0.864871000000	9.921709000000
C	-6.704460000000	0.691695000000	8.163762000000
C	-7.470075000000	-1.191681000000	10.081926000000
H	-5.362613000000	-1.351622000000	10.551930000000
C	-8.058043000000	0.367626000000	8.321831000000
H	-6.409113000000	1.443580000000	7.422710000000
C	-8.446924000000	-0.577730000000	9.285670000000
H	-7.761188000000	-1.928540000000	10.836702000000
H	-8.811064000000	0.854825000000	7.694561000000
H	-9.503192000000	-0.831898000000	9.413222000000
C	-3.245242000000	-0.259861000000	9.628898000000
C	-2.577615000000	-1.419650000000	9.188303000000
C	-2.977783000000	0.215172000000	10.928313000000
C	-1.662592000000	-2.087228000000	10.018419000000
H	-2.782454000000	-1.807127000000	8.183493000000
C	-2.068122000000	-0.451347000000	11.763095000000
H	-3.484145000000	1.115654000000	11.290585000000
C	-1.404123000000	-1.603321000000	11.310111000000
H	-1.156192000000	-2.987795000000	9.657224000000
H	-1.878173000000	-0.069977000000	12.771143000000
H	-0.694244000000	-2.121783000000	11.961371000000
C	-2.818811000000	5.870431000000	5.873061000000
C	-4.089758000000	5.809808000000	5.263886000000
C	-2.152865000000	7.107770000000	5.902804000000
C	-4.682748000000	6.949694000000	4.710375000000
H	-4.621652000000	4.852587000000	5.220276000000
C	-2.744865000000	8.256290000000	5.347833000000
H	-1.160046000000	7.187228000000	6.353944000000
C	-4.011118000000	8.183512000000	4.753071000000
H	-5.666185000000	6.874290000000	4.236316000000
H	-2.207221000000	9.208875000000	5.383650000000
H	-4.472868000000	9.077912000000	4.323914000000
C	-0.774049000000	4.727432000000	6.928751000000
C	0.291363000000	4.218363000000	6.161686000000

C	-0.475789000000	5.375274000000	8.146414000000
C	1.622558000000	4.347616000000	6.600731000000
H	0.089020000000	3.696329000000	5.221242000000
C	0.850123000000	5.521126000000	8.581781000000
H	-1.291019000000	5.767462000000	8.763111000000
C	1.906139000000	5.004426000000	7.811198000000
H	2.438867000000	3.915695000000	6.011625000000
H	1.058701000000	6.033652000000	9.525895000000
H	2.940973000000	5.105806000000	8.152653000000
C	0.935384000000	-0.171955000000	6.932979000000
C	1.292550000000	-1.459626000000	6.442984000000
C	1.957526000000	0.642038000000	7.494596000000
C	2.597710000000	-1.922790000000	6.527901000000
H	0.517255000000	-2.089915000000	6.000629000000
C	3.269694000000	0.193058000000	7.567934000000
H	1.741459000000	1.660203000000	7.819356000000
C	3.598151000000	-1.093511000000	7.088288000000
H	2.855841000000	-2.918417000000	6.151324000000
H	4.064870000000	0.831807000000	7.959902000000
O	4.895470000000	-1.471017000000	7.184934000000
H	5.018864000000	-2.363799000000	6.806949000000
C	-4.497344000000	1.365326000000	4.678499000000
H	-4.184933000000	2.084683000000	5.455516000000
H	-5.597412000000	1.376687000000	4.713568000000
C	-3.960764000000	1.712398000000	3.286680000000
H	-4.066185000000	2.811280000000	3.181993000000
H	-2.872009000000	1.525488000000	3.215281000000
C	-3.492504000000	-1.369219000000	5.006779000000
H	-3.307258000000	-2.076341000000	5.832864000000
C	-4.715841000000	-0.612504000000	4.918553000000
H	-5.220965000000	-0.600955000000	3.949913000000
H	-3.542437000000	-2.341585000000	4.486749000000
Cl	-5.521928000000	-1.002549000000	6.438463000000

P_{vCl}

O	6.078638000000	1.835136000000	-0.455405000000
Si	4.544928000000	1.950671000000	0.112602000000
O	4.308300000000	0.710265000000	1.153257000000
Si	3.301827000000	-0.467125000000	1.645908000000
O	3.264410000000	-1.804584000000	0.679083000000
O	3.402750000000	1.769187000000	-1.092917000000
O	4.240017000000	3.393869000000	0.859359000000
Si	4.690181000000	4.342582000000	2.112069000000
O	3.390670000000	4.874781000000	2.936496000000
Si	2.270981000000	6.086147000000	3.034375000000
O	0.799311000000	5.335487000000	2.841079000000
O	1.796348000000	0.060222000000	1.915144000000
Si	0.883257000000	1.276260000000	2.567930000000
O	-0.680687000000	0.714820000000	2.613156000000
O	4.071810000000	-0.999147000000	3.069997000000
O	5.703951000000	5.538558000000	1.668666000000
Si	5.933005000000	6.760345000000	0.578676000000

O	5.159805000000	8.048961000000	1.363257000000
O	5.339721000000	6.464769000000	-0.913483000000
O	7.562535000000	6.989775000000	0.384708000000
O	5.611063000000	3.443069000000	3.172718000000
Si	5.396691000000	2.417636000000	4.465595000000
O	4.048459000000	1.466213000000	4.337293000000
O	0.857869000000	2.651299000000	1.644971000000
Si	0.176034000000	3.201556000000	0.214705000000
O	-1.370183000000	3.690221000000	0.469273000000
Si	-2.242909000000	4.945039000000	1.136648000000
O	-3.843643000000	4.628777000000	0.990255000000
O	0.169945000000	2.011747000000	-0.931560000000
O	1.145707000000	4.484488000000	-0.135054000000
Si	1.419170000000	5.619273000000	-1.319057000000
O	2.585745000000	6.645292000000	-0.725040000000
O	2.146935000000	4.849745000000	-2.608405000000
O	0.029308000000	6.335417000000	-1.825791000000
O	1.371607000000	1.657644000000	4.087038000000
O	6.794420000000	1.532944000000	4.512936000000
Si	7.816027000000	0.673661000000	3.543687000000
O	6.860416000000	-0.408061000000	2.652646000000
O	8.639834000000	1.551526000000	2.433649000000
Si	9.058834000000	3.091816000000	2.009837000000
O	10.685033000000	3.267305000000	1.815808000000
O	8.311114000000	3.253444000000	0.513896000000
O	8.530932000000	4.213046000000	3.111249000000
O	8.793538000000	-0.123797000000	4.599755000000
O	5.162382000000	3.252462000000	5.867241000000
O	-1.771840000000	6.293361000000	0.227442000000
O	-1.981392000000	5.182400000000	2.743094000000
O	2.537189000000	7.254847000000	1.882273000000
O	2.350124000000	6.862591000000	4.490150000000
H	6.816893000000	2.397854000000	-0.112822000000
H	3.408864000000	2.488742000000	-1.756093000000
H	5.063253000000	-0.990877000000	2.938715000000
H	-0.776126000000	-0.133699000000	3.082001000000
H	2.343836000000	1.708631000000	4.273409000000
H	-4.179878000000	4.312275000000	0.126678000000
H	-1.048308000000	5.416500000000	2.962327000000
H	2.827416000000	-1.726893000000	-0.186767000000
H	0.911938000000	4.395411000000	2.559244000000
H	2.039958000000	6.361632000000	5.264345000000
H	5.870101000000	3.859235000000	6.143253000000
H	4.161462000000	0.534865000000	4.020403000000
H	11.180951000000	3.635765000000	2.566685000000
H	8.506112000000	4.083732000000	0.039106000000
H	7.553130000000	4.238303000000	3.197822000000
H	5.242875000000	8.910884000000	0.915315000000
H	8.061551000000	7.139372000000	1.206883000000
H	6.900367000000	-0.235256000000	1.691607000000
H	9.497963000000	-0.676425000000	4.220853000000
H	-0.690798000000	6.391269000000	-1.146418000000

H	1.702512000000	4.983417000000	-3.462879000000
H	1.077541000000	1.728939000000	-1.169294000000
H	-2.146222000000	7.137790000000	0.535592000000
H	3.432200000000	7.689370000000	1.905192000000
H	4.347470000000	6.501071000000	-0.998471000000
H	2.440907000000	6.928058000000	0.220417000000
Ni	-4.678986000000	-2.263698000000	3.683772000000
N	-4.592205000000	-2.863651000000	5.490379000000
C	-5.471859000000	3.729330000000	-1.676147000000
H	-6.424505000000	4.097152000000	-1.277343000000
H	-4.971796000000	4.362244000000	-2.416659000000
C	-4.966870000000	2.533586000000	-1.308677000000
C	-5.592545000000	1.597730000000	-0.306488000000
H	-6.587754000000	1.976434000000	-0.011866000000
H	-5.751938000000	0.613080000000	-0.790502000000
C	-2.341776000000	-2.721783000000	4.951389000000
C	-3.371415000000	-3.028060000000	5.975780000000
H	-4.014131000000	2.200296000000	-1.744176000000
O	-2.833814000000	-2.360261000000	3.806668000000
C	-3.077944000000	-3.517169000000	7.356136000000
H	-2.361578000000	-4.354209000000	7.317245000000
H	-2.648414000000	-2.708947000000	7.973250000000
H	-3.996803000000	-3.858749000000	7.847895000000
C	-5.748966000000	-3.065321000000	6.305780000000
C	-6.277023000000	-1.968042000000	7.033920000000
C	-6.386820000000	-4.333313000000	6.293862000000
C	-7.396756000000	-2.204112000000	7.843863000000
C	-7.502411000000	-4.510506000000	7.125959000000
C	-8.006144000000	-3.471035000000	7.929772000000
H	-7.833150000000	-1.362248000000	8.391651000000
H	-8.016800000000	-5.477083000000	7.113264000000
C	-9.190639000000	-3.697286000000	8.837356000000
H	-8.858840000000	-3.997141000000	9.847867000000
H	-9.792717000000	-2.781921000000	8.950911000000
H	-9.843594000000	-4.498355000000	8.457159000000
C	-5.865337000000	-5.453727000000	5.384870000000
H	-5.493684000000	-4.949385000000	4.469368000000
C	-5.668930000000	-0.568753000000	6.874922000000
H	-5.331145000000	-0.513545000000	5.819498000000
C	-6.981941000000	-6.396413000000	4.911943000000
C	-7.082579000000	-7.737608000000	5.326367000000
C	-7.946319000000	-5.893370000000	4.012367000000
C	-8.128109000000	-8.553750000000	4.862417000000
H	-6.337949000000	-8.158300000000	6.008057000000
C	-8.987950000000	-6.707157000000	3.548141000000
H	-7.878701000000	-4.851324000000	3.681354000000
C	-9.085164000000	-8.042311000000	3.974443000000
H	-8.187679000000	-9.594560000000	5.195257000000
H	-9.725157000000	-6.298093000000	2.850263000000
H	-9.897207000000	-8.679193000000	3.611151000000
C	-4.661695000000	-6.180719000000	5.988337000000
C	-3.537338000000	-6.454618000000	5.183607000000

C	-4.646208000000	-6.610378000000	7.330592000000
C	-2.423498000000	-7.132373000000	5.704055000000
H	-3.542318000000	-6.142947000000	4.132293000000
C	-3.537401000000	-7.294362000000	7.853686000000
H	-5.508147000000	-6.404808000000	7.974294000000
C	-2.419560000000	-7.553452000000	7.043867000000
H	-1.562067000000	-7.338818000000	5.061112000000
H	-3.547732000000	-7.626767000000	8.896515000000
H	-1.555842000000	-8.086906000000	7.451593000000
C	-6.716712000000	0.545539000000	7.024200000000
C	-7.734675000000	0.639189000000	6.050333000000
C	-6.702095000000	1.479019000000	8.076404000000
C	-8.713267000000	1.638020000000	6.131178000000
H	-7.758527000000	-0.085486000000	5.228245000000
C	-7.685502000000	2.480865000000	8.158900000000
H	-5.916657000000	1.437905000000	8.836077000000
C	-8.693115000000	2.564396000000	7.188332000000
H	-9.494216000000	1.695841000000	5.366411000000
H	-7.656152000000	3.198331000000	8.984721000000
H	-9.457395000000	3.344746000000	7.251075000000
C	-4.416214000000	-0.365059000000	7.727916000000
C	-3.280452000000	0.252495000000	7.164758000000
C	-4.358287000000	-0.774015000000	9.075262000000
C	-2.114285000000	0.450036000000	7.921480000000
H	-3.314616000000	0.594433000000	6.123660000000
C	-3.197479000000	-0.570218000000	9.839250000000
H	-5.226579000000	-1.262583000000	9.529894000000
C	-2.068966000000	0.037303000000	9.263347000000
H	-1.246628000000	0.932716000000	7.461034000000
H	-3.175648000000	-0.885399000000	10.887297000000
H	-1.165970000000	0.197260000000	9.860321000000
C	-0.903034000000	-2.784179000000	5.071981000000
C	-0.150003000000	-2.828160000000	3.858873000000
C	-0.184229000000	-2.732180000000	6.301518000000
C	1.233579000000	-2.789045000000	3.866895000000
H	-0.684197000000	-2.924190000000	2.909868000000
C	1.202492000000	-2.673748000000	6.316090000000
H	-0.711938000000	-2.658707000000	7.251558000000
C	1.929821000000	-2.669492000000	5.100703000000
H	1.791822000000	-2.863596000000	2.929646000000
H	1.751798000000	-2.579839000000	7.256094000000
O	3.262731000000	-2.551156000000	5.166459000000
H	3.628932000000	-2.293053000000	4.277767000000
C	-5.288727000000	0.517769000000	2.044203000000
H	-4.677403000000	0.651833000000	2.954173000000
H	-6.317680000000	0.830753000000	2.285494000000
C	-4.693236000000	1.430589000000	0.938637000000
H	-4.508823000000	2.418021000000	1.394471000000
H	-3.700670000000	1.048667000000	0.635995000000
C	-4.148002000000	-1.716339000000	1.675239000000
H	-4.113839000000	-2.695664000000	1.183497000000
C	-5.274808000000	-0.923016000000	1.623637000000

H	-6.180550000000	-1.312071000000	1.147223000000
H	-3.192486000000	-1.237415000000	1.904983000000
Cl	-6.840295000000	-2.150591000000	3.539821000000

ACl

C	4.194904000000	-0.039744000000	-0.524349000000
H	4.115419000000	-0.792599000000	-1.305397000000
H	5.123948000000	0.519181000000	-0.470972000000
C	3.197192000000	0.181920000000	0.330172000000
H	3.318400000000	0.947736000000	1.097434000000
C	1.878142000000	-0.535831000000	0.321878000000
H	1.885714000000	-1.316693000000	-0.448220000000
H	1.734521000000	-1.042200000000	1.286274000000
C	0.696437000000	0.413468000000	0.083104000000
H	0.827714000000	0.908260000000	-0.887557000000
H	0.710176000000	1.207106000000	0.842328000000
C	-0.650929000000	-0.311367000000	0.121904000000
H	-0.786034000000	-0.797731000000	1.094833000000
H	-0.671687000000	-1.102400000000	-0.636412000000
C	-1.801805000000	0.649220000000	-0.123586000000
H	-1.727361000000	1.120084000000	-1.105576000000
H	-1.842307000000	1.428673000000	0.639625000000
Cl	-3.398362000000	-0.189730000000	-0.080065000000

I_{ACl}

O	6.070522000000	1.873059000000	-0.483491000000
Si	4.544918000000	1.950604000000	0.112435000000
O	4.333342000000	0.705486000000	1.150218000000
Si	3.301784000000	-0.467008000000	1.645935000000
O	3.345342000000	-1.824342000000	0.731049000000
O	3.386611000000	1.789610000000	-1.078558000000
O	4.240885000000	3.393209000000	0.869711000000
Si	4.690172000000	4.342462000000	2.112298000000
O	3.380888000000	4.867380000000	2.929348000000
Si	2.270969000000	6.086178000000	3.034351000000
O	0.811154000000	5.324343000000	2.817132000000
O	1.766342000000	0.100960000000	1.804899000000
Si	0.883309000000	1.276225000000	2.567988000000
O	-0.661742000000	0.713697000000	2.796830000000
O	3.928849000000	-0.864342000000	3.164229000000
O	5.695510000000	5.550370000000	1.682906000000
Si	5.933025000000	6.760388000000	0.578627000000
O	5.146276000000	8.053783000000	1.342788000000
O	5.350330000000	6.450532000000	-0.914973000000
O	7.563352000000	6.991508000000	0.397075000000
O	5.601966000000	3.459616000000	3.195154000000
Si	5.396700000000	2.417633000000	4.465587000000
O	4.061269000000	1.459915000000	4.395942000000
O	0.846877000000	2.658243000000	1.643188000000
Si	0.176072000000	3.201554000000	0.214590000000
O	-1.381329000000	3.674517000000	0.490170000000
Si	-2.242933000000	4.945045000000	1.136663000000

O	-3.832478000000	4.528052000000	1.284504000000
O	0.161380000000	2.018815000000	-0.932922000000
O	1.109031000000	4.511921000000	-0.120673000000
Si	1.419162000000	5.619252000000	-1.319008000000
O	2.594730000000	6.636380000000	-0.726484000000
O	2.153875000000	4.828350000000	-2.589538000000
O	0.048129000000	6.364921000000	-1.836238000000
O	1.456645000000	1.686147000000	4.044398000000
O	6.812943000000	1.573255000000	4.529314000000
Si	7.816018000000	0.673787000000	3.543640000000
O	6.874899000000	-0.438277000000	2.709552000000
O	8.600820000000	1.562015000000	2.415416000000
Si	9.058832000000	3.091740000000	2.009869000000
O	10.689337000000	3.245745000000	1.834154000000
O	8.324405000000	3.275233000000	0.511500000000
O	8.539599000000	4.221164000000	3.111970000000
O	8.818690000000	-0.070446000000	4.615487000000
O	5.129170000000	3.284456000000	5.880777000000
O	-1.994766000000	6.187539000000	0.018755000000
O	-1.830602000000	5.395803000000	2.664502000000
O	2.490186000000	7.271043000000	1.889575000000
O	2.382877000000	6.809403000000	4.513759000000
H	6.810922000000	2.424701000000	-0.131487000000
H	3.403690000000	2.501401000000	-1.749560000000
H	4.879304000000	-1.122634000000	3.103069000000
H	-0.934476000000	0.769102000000	3.736735000000
H	2.436797000000	1.717622000000	4.204833000000
H	-4.214933000000	3.956347000000	0.583600000000
H	-0.859058000000	5.548405000000	2.808301000000
H	2.749252000000	-1.941677000000	-0.039644000000
H	0.927439000000	4.382283000000	2.538078000000
H	2.563840000000	6.195275000000	5.258380000000
H	5.894911000000	3.791842000000	6.206077000000
H	4.111157000000	0.536400000000	3.982788000000
H	11.184311000000	3.594593000000	2.594904000000
H	8.540206000000	4.097576000000	0.032539000000
H	7.562803000000	4.249472000000	3.196495000000
H	5.242352000000	8.911166000000	0.888767000000
H	8.063131000000	7.097468000000	1.225599000000
H	6.810550000000	-0.250181000000	1.752914000000
H	9.463698000000	-0.699866000000	4.250312000000
H	-0.728401000000	6.331479000000	-1.222029000000
H	1.702095000000	4.926864000000	-3.445123000000
H	1.059026000000	1.685767000000	-1.144903000000
H	-2.433485000000	7.028823000000	0.239088000000
H	3.374712000000	7.722721000000	1.919970000000
H	4.359025000000	6.491072000000	-1.001817000000
H	2.436762000000	6.935798000000	0.209950000000
Ni	-3.720471000000	0.162226000000	4.655727000000
N	-4.441202000000	1.661365000000	5.759726000000
C	-5.182682000000	2.568262000000	-0.975172000000
H	-6.254546000000	2.762821000000	-0.854622000000

H	-4.665372000000	3.108256000000	-1.774992000000
C	-4.535455000000	1.673020000000	-0.201494000000
C	-5.174067000000	0.840067000000	0.877345000000
H	-6.261453000000	1.027395000000	0.913266000000
H	-5.037889000000	-0.230341000000	0.627535000000
C	-2.133872000000	1.884780000000	6.149308000000
C	-3.537318000000	2.326983000000	6.449800000000
H	-3.458315000000	1.515974000000	-0.358336000000
O	-2.038280000000	0.755119000000	5.549449000000
C	-3.823107000000	3.286829000000	7.561168000000
H	-3.136125000000	3.099854000000	8.401866000000
H	-3.685700000000	4.331540000000	7.235113000000
H	-4.860090000000	3.185352000000	7.906415000000
C	-5.845499000000	1.834989000000	6.067337000000
C	-6.608205000000	2.886222000000	5.496280000000
C	-6.453207000000	0.876363000000	6.925317000000
C	-7.975338000000	2.961273000000	5.828316000000
C	-7.817437000000	1.009765000000	7.223676000000
C	-8.599825000000	2.051379000000	6.694558000000
H	-8.574433000000	3.751794000000	5.364228000000
H	-8.284637000000	0.260575000000	7.871812000000
C	-10.061188000000	2.185083000000	7.050745000000
H	-10.185886000000	2.705787000000	8.017136000000
H	-10.610243000000	2.763999000000	6.291978000000
H	-10.543560000000	1.199347000000	7.149605000000
C	-5.621596000000	-0.248809000000	7.548146000000
H	-4.843541000000	-0.496063000000	6.797454000000
C	-6.002027000000	3.942153000000	4.563874000000
H	-5.120869000000	3.486379000000	4.074674000000
C	-6.421109000000	-1.542059000000	7.756520000000
C	-6.548057000000	-2.167912000000	9.009569000000
C	-7.031044000000	-2.150738000000	6.637358000000
C	-7.268727000000	-3.367595000000	9.143132000000
H	-6.080749000000	-1.720449000000	9.891326000000
C	-7.748476000000	-3.346465000000	6.767565000000
H	-6.954349000000	-1.669008000000	5.655744000000
C	-7.870699000000	-3.961085000000	8.025639000000
H	-7.356907000000	-3.836305000000	10.127997000000
H	-8.216003000000	-3.799191000000	5.887497000000
H	-8.431319000000	-4.894600000000	8.130570000000
C	-4.862651000000	0.220019000000	8.792412000000
C	-3.516660000000	-0.157547000000	8.972699000000
C	-5.475096000000	1.009071000000	9.786304000000
C	-2.797415000000	0.244625000000	10.109389000000
H	-3.027792000000	-0.783084000000	8.216541000000
C	-4.761791000000	1.409316000000	10.926849000000
H	-6.519228000000	1.315776000000	9.666277000000
C	-3.418893000000	1.032418000000	11.091001000000
H	-1.754076000000	-0.063143000000	10.231113000000
H	-5.257489000000	2.017236000000	11.690015000000
H	-2.863121000000	1.345942000000	11.979644000000
C	-6.962929000000	4.334247000000	3.424186000000

C	-7.570919000000	3.323071000000	2.649647000000
C	-7.233249000000	5.674604000000	3.094989000000
C	-8.422787000000	3.639912000000	1.583155000000
H	-7.393238000000	2.271619000000	2.899767000000
C	-8.086611000000	5.996299000000	2.025767000000
H	-6.774168000000	6.479913000000	3.674515000000
C	-8.684949000000	4.982951000000	1.264993000000
H	-8.891969000000	2.836700000000	1.005369000000
H	-8.283962000000	7.047086000000	1.792303000000
H	-9.353925000000	5.234091000000	0.436475000000
C	-5.461889000000	5.154673000000	5.333009000000
C	-4.248452000000	5.740728000000	4.919302000000
C	-6.146983000000	5.722572000000	6.425233000000
C	-3.727209000000	6.859524000000	5.590044000000
H	-3.704861000000	5.335127000000	4.059490000000
C	-5.626125000000	6.840066000000	7.097248000000
H	-7.090910000000	5.285106000000	6.764750000000
C	-4.410931000000	7.411170000000	6.685834000000
H	-2.788637000000	7.303591000000	5.242070000000
H	-6.172762000000	7.265335000000	7.944716000000
H	-4.006263000000	8.282572000000	7.209550000000
C	-0.936050000000	2.622178000000	6.511069000000
C	0.273039000000	1.912446000000	6.744619000000
C	-0.890399000000	4.044884000000	6.466142000000
C	1.483949000000	2.580463000000	6.872019000000
H	0.245602000000	0.821737000000	6.806973000000
C	0.325506000000	4.713924000000	6.535205000000
H	-1.795737000000	4.625401000000	6.271976000000
C	1.525518000000	3.978960000000	6.675904000000
H	2.410471000000	2.022133000000	7.036276000000
H	0.372117000000	5.799283000000	6.413827000000
O	2.693218000000	4.671913000000	6.586385000000
H	3.476772000000	4.070765000000	6.473630000000
C	-5.122295000000	0.145683000000	3.341003000000
H	-6.035188000000	0.513800000000	3.839266000000
H	-5.301898000000	-0.864135000000	2.939642000000
C	-4.551913000000	1.093888000000	2.283609000000
H	-4.715354000000	2.147378000000	2.561001000000
H	-3.453729000000	0.975756000000	2.181769000000
C	-3.721998000000	-1.866436000000	4.798569000000
H	-4.639867000000	-2.328608000000	4.420161000000
H	-3.532641000000	-2.013487000000	5.869040000000
C	-2.675247000000	-1.517963000000	3.938894000000
H	-1.698428000000	-1.322500000000	4.402855000000
C	-2.599863000000	-1.762293000000	2.451832000000
H	-3.561602000000	-2.128725000000	2.054718000000
H	-2.360345000000	-0.810509000000	1.947395000000
C	-1.455663000000	-2.751439000000	2.109811000000
H	-1.787766000000	-3.790304000000	2.289267000000
H	-0.607513000000	-2.569271000000	2.795934000000
C	-0.955200000000	-2.565183000000	0.666125000000
H	-0.618723000000	-1.520699000000	0.545586000000

H	-1.771626000000	-2.728859000000	-0.059747000000
C	0.207868000000	-3.499402000000	0.352748000000
H	-0.106600000000	-4.546217000000	0.229249000000
H	0.994427000000	-3.447377000000	1.121185000000
Cl	1.025822000000	-3.037817000000	-1.215772000000

TS$_{ACl}$

O	6.107776000000	1.460965000000	0.419833000000
Si	4.544916000000	1.950601000000	0.112425000000
O	3.639287000000	0.638285000000	0.485983000000
Si	3.301801000000	-0.466998000000	1.645939000000
O	3.200203000000	-1.986080000000	1.037421000000
O	4.350490000000	2.363041000000	-1.466500000000
O	4.094020000000	3.256506000000	1.041136000000
Si	4.690164000000	4.342439000000	2.112289000000
O	3.481974000000	4.982558000000	2.973261000000
Si	2.270965000000	6.086186000000	3.034352000000
O	0.846632000000	5.236375000000	2.918131000000
O	1.889061000000	-0.024811000000	2.421555000000
Si	0.883280000000	1.276212000000	2.567982000000
O	-0.611671000000	0.783208000000	3.050001000000
O	4.397302000000	-0.511809000000	2.897935000000
O	5.743722000000	5.392645000000	1.480343000000
Si	5.933026000000	6.760401000000	0.578624000000
O	5.092291000000	7.922957000000	1.488184000000
O	5.342709000000	6.633113000000	-0.940399000000
O	7.549836000000	7.069356000000	0.456993000000
O	5.637463000000	3.508668000000	3.227288000000
Si	5.396723000000	2.417644000000	4.465609000000
O	3.990844000000	1.573735000000	4.340249000000
O	0.829749000000	1.976217000000	1.076556000000
Si	0.176078000000	3.201566000000	0.214596000000
O	-1.246433000000	3.636728000000	0.953009000000
Si	-2.242930000000	4.945042000000	1.136663000000
O	-3.785999000000	4.475256000000	1.522526000000
O	-0.115551000000	2.600135000000	-1.314562000000
O	1.083129000000	4.564500000000	-0.029712000000
Si	1.419161000000	5.619250000000	-1.319011000000
O	2.525425000000	6.719653000000	-0.759965000000
O	2.293166000000	4.794089000000	-2.478748000000
O	0.027563000000	6.248405000000	-1.922648000000
O	1.463068000000	2.435550000000	3.616504000000
O	6.766986000000	1.513162000000	4.524894000000
Si	7.816010000000	0.673772000000	3.543637000000
O	6.955297000000	-0.169039000000	2.382378000000
O	8.880149000000	1.650696000000	2.770277000000
Si	9.058833000000	3.091745000000	2.009870000000
O	10.647033000000	3.510597000000	1.865735000000
O	8.357689000000	2.868800000000	0.482461000000
O	8.385762000000	4.366533000000	2.812887000000
O	8.567097000000	-0.362596000000	4.572813000000
O	5.185646000000	3.240918000000	5.917690000000

O	-2.223425000000	5.751363000000	-0.348851000000
O	-1.768238000000	5.893230000000	2.396996000000
O	2.373780000000	7.248423000000	1.853829000000
O	2.415714000000	6.878535000000	4.484455000000
H	6.875579000000	2.077231000000	0.254413000000
H	3.790624000000	3.137428000000	-1.684682000000
H	5.383427000000	-0.579496000000	2.704917000000
H	-0.947193000000	1.050572000000	3.932460000000
H	2.386629000000	2.247772000000	3.932443000000
H	-4.276108000000	3.965607000000	0.844703000000
H	-0.812879000000	5.765764000000	2.632816000000
H	2.562981000000	-2.181323000000	0.319225000000
H	0.976853000000	4.285101000000	3.166700000000
H	2.713493000000	6.311919000000	5.225575000000
H	5.975233000000	3.692184000000	6.267330000000
H	4.108531000000	0.683469000000	3.846362000000
H	11.259750000000	2.833753000000	1.533252000000
H	8.530021000000	3.595814000000	-0.145226000000
H	7.418880000000	4.273729000000	2.962317000000
H	5.316247000000	8.844001000000	1.260876000000
H	8.081141000000	6.807376000000	1.231386000000
H	6.741418000000	0.364672000000	1.558499000000
H	9.214499000000	-0.977999000000	4.189281000000
H	-0.794176000000	6.053605000000	-1.406519000000
H	1.740116000000	4.367430000000	-3.157364000000
H	-0.169658000000	1.632063000000	-1.382215000000
H	-2.760719000000	6.564656000000	-0.365966000000
H	3.256254000000	7.707533000000	1.881394000000
H	4.355219000000	6.681704000000	-1.030722000000
H	2.348447000000	6.991343000000	0.181797000000
Ni	-4.121668000000	0.774853000000	4.897312000000
N	-4.529747000000	2.221850000000	6.210351000000
C	-5.577285000000	2.528979000000	-0.692255000000
H	-6.631335000000	2.828857000000	-0.672630000000
H	-4.959284000000	2.940724000000	-1.496681000000
C	-5.073984000000	1.662476000000	0.208360000000
C	-5.848642000000	1.023517000000	1.332001000000
H	-6.930610000000	1.209048000000	1.205435000000
H	-5.703127000000	-0.073182000000	1.285074000000
C	-2.204966000000	2.273499000000	6.162747000000
C	-3.469101000000	2.775279000000	6.764105000000
H	-4.010546000000	1.388320000000	0.144838000000
O	-2.323581000000	1.252281000000	5.380554000000
C	-3.484927000000	3.682908000000	7.955579000000
H	-2.618306000000	3.485190000000	8.603737000000
H	-3.456327000000	4.743134000000	7.651272000000
H	-4.407433000000	3.537977000000	8.534079000000
C	-5.831072000000	2.484180000000	6.770860000000
C	-6.597574000000	3.591159000000	6.323316000000
C	-6.351552000000	1.566987000000	7.723342000000
C	-7.850939000000	3.808924000000	6.924501000000
C	-7.607548000000	1.838716000000	8.289379000000

C	-8.363592000000	2.968214000000	7.926639000000
H	-8.457067000000	4.649643000000	6.570581000000
H	-8.019159000000	1.128228000000	9.014267000000
C	-9.691867000000	3.258177000000	8.584082000000
H	-10.212952000000	2.330313000000	8.868945000000
H	-9.553103000000	3.850320000000	9.506444000000
H	-10.353360000000	3.836906000000	7.920551000000
C	-5.548203000000	0.323807000000	8.123700000000
H	-4.949169000000	0.048905000000	7.227750000000
C	-6.092266000000	4.514147000000	5.207119000000
H	-5.425047000000	3.901328000000	4.569985000000
C	-6.433609000000	-0.896244000000	8.422449000000
C	-6.269870000000	-1.683978000000	9.576429000000
C	-7.413138000000	-1.285539000000	7.481657000000
C	-7.062411000000	-2.825562000000	9.786872000000
H	-5.518147000000	-1.407882000000	10.320963000000
C	-8.203450000000	-2.423983000000	7.688178000000
H	-7.578098000000	-0.671434000000	6.588914000000
C	-8.029693000000	-3.201612000000	8.845355000000
H	-6.919043000000	-3.420419000000	10.694036000000
H	-8.960542000000	-2.702013000000	6.948174000000
H	-8.646892000000	-4.089454000000	9.010894000000
C	-4.521819000000	0.625873000000	9.218606000000
C	-3.199833000000	0.156923000000	9.086108000000
C	-4.864363000000	1.348451000000	10.378676000000
C	-2.241190000000	0.404932000000	10.081741000000
H	-2.922211000000	-0.419683000000	8.195542000000
C	-3.910586000000	1.595373000000	11.377990000000
H	-5.885940000000	1.722300000000	10.502482000000
C	-2.594303000000	1.127798000000	11.231736000000
H	-1.220611000000	0.027940000000	9.961739000000
H	-4.197233000000	2.154096000000	12.274240000000
H	-1.851211000000	1.321430000000	12.010927000000
C	-7.229122000000	4.997751000000	4.286529000000
C	-8.097974000000	4.051220000000	3.700163000000
C	-7.415199000000	6.355081000000	3.967716000000
C	-9.117927000000	4.447071000000	2.824739000000
H	-7.991368000000	2.990339000000	3.952826000000
C	-8.438134000000	6.755834000000	3.091019000000
H	-6.756001000000	7.110771000000	4.403128000000
C	-9.292329000000	5.806258000000	2.514838000000
H	-9.783494000000	3.694586000000	2.389625000000
H	-8.564108000000	7.818395000000	2.861708000000
H	-10.089952000000	6.119442000000	1.834726000000
C	-5.213636000000	5.655950000000	5.725715000000
C	-4.064172000000	6.021177000000	4.996309000000
C	-5.534999000000	6.380338000000	6.890792000000
C	-3.248374000000	7.080655000000	5.425911000000
H	-3.809271000000	5.492996000000	4.070863000000
C	-4.719489000000	7.438602000000	7.323255000000
H	-6.424172000000	6.113058000000	7.470810000000
C	-3.570920000000	7.790378000000	6.594370000000

H	-2.372108000000	7.351706000000	4.828799000000
H	-4.984492000000	7.991245000000	8.230038000000
H	-2.939169000000	8.618603000000	6.929793000000
C	-0.898710000000	2.844459000000	6.443527000000
C	0.260166000000	2.025792000000	6.511306000000
C	-0.740374000000	4.254696000000	6.556360000000
C	1.526494000000	2.585319000000	6.652456000000
H	0.151923000000	0.937901000000	6.464622000000
C	0.523571000000	4.821104000000	6.641965000000
H	-1.606363000000	4.916612000000	6.480320000000
C	1.670539000000	3.993551000000	6.644886000000
H	2.412062000000	1.944633000000	6.700159000000
H	0.648309000000	5.907013000000	6.650061000000
O	2.872112000000	4.620307000000	6.609552000000
H	3.635266000000	3.995532000000	6.457157000000
C	-5.974452000000	0.764703000000	3.916037000000
H	-6.295268000000	1.392170000000	4.762639000000
H	-6.872509000000	0.198016000000	3.637586000000
C	-5.404683000000	1.546661000000	2.717827000000
H	-5.711394000000	2.603051000000	2.809456000000
H	-4.293117000000	1.583505000000	2.745282000000
C	-5.139748000000	-0.983535000000	4.271959000000
H	-5.637888000000	-1.384481000000	3.381965000000
H	-5.615099000000	-1.334046000000	5.197672000000
C	-3.692692000000	-0.988839000000	4.178926000000
H	-3.119451000000	-1.393428000000	5.028477000000
C	-3.035488000000	-1.109869000000	2.815711000000
H	-3.819109000000	-1.268066000000	2.050161000000
H	-2.522358000000	-0.168665000000	2.546894000000
C	-1.982574000000	-2.231564000000	2.711206000000
H	-2.442494000000	-3.211705000000	2.937333000000
H	-1.197256000000	-2.056758000000	3.469315000000
C	-1.348781000000	-2.243042000000	1.308607000000
H	-0.954551000000	-1.235418000000	1.089614000000
H	-2.118092000000	-2.463144000000	0.546384000000
C	-0.215284000000	-3.252525000000	1.186631000000
H	-0.558135000000	-4.291433000000	1.302680000000
H	0.596959000000	-3.053095000000	1.901795000000
Cl	0.550889000000	-3.168469000000	-0.472381000000

P_{ACl}

O	6.109180000000	1.466178000000	0.418452000000
Si	4.544913000000	1.950604000000	0.112428000000
O	3.643505000000	0.642761000000	0.492298000000
Si	3.301803000000	-0.467001000000	1.645936000000
O	3.146391000000	-1.959637000000	0.972809000000
O	4.345935000000	2.364666000000	-1.465509000000
O	4.087999000000	3.256025000000	1.044193000000
Si	4.690196000000	4.342425000000	2.112309000000
O	3.495365000000	4.998586000000	2.980127000000
Si	2.270959000000	6.086188000000	3.034353000000
O	0.860659000000	5.213769000000	2.926695000000

O	1.899039000000	-0.012046000000	2.428127000000
Si	0.883279000000	1.276215000000	2.567997000000
O	-0.540199000000	0.701808000000	3.160459000000
O	4.394821000000	-0.556677000000	2.893467000000
O	5.743099000000	5.384998000000	1.466052000000
Si	5.933024000000	6.760401000000	0.578622000000
O	5.084587000000	7.915357000000	1.492318000000
O	5.348131000000	6.646754000000	-0.944165000000
O	7.548945000000	7.076530000000	0.467574000000
O	5.640733000000	3.511821000000	3.227295000000
Si	5.396683000000	2.417660000000	4.465597000000
O	3.994228000000	1.566868000000	4.315199000000
O	0.789944000000	1.963519000000	1.078240000000
Si	0.176059000000	3.201566000000	0.214580000000
O	-1.267142000000	3.626869000000	0.928286000000
Si	-2.242912000000	4.945034000000	1.136667000000
O	-3.802389000000	4.502782000000	1.493193000000
O	-0.090169000000	2.594763000000	-1.316713000000
O	1.071865000000	4.575740000000	-0.017405000000
Si	1.419163000000	5.619256000000	-1.319010000000
O	2.519984000000	6.725087000000	-0.762679000000
O	2.297645000000	4.782043000000	-2.466028000000
O	0.029040000000	6.239488000000	-1.936594000000
O	1.494086000000	2.479180000000	3.555706000000
O	6.770016000000	1.517716000000	4.526381000000
Si	7.816031000000	0.673774000000	3.543630000000
O	6.952523000000	-0.166619000000	2.382526000000
O	8.880547000000	1.650836000000	2.770313000000
Si	9.058830000000	3.091740000000	2.009869000000
O	10.646421000000	3.514291000000	1.868535000000
O	8.363481000000	2.865246000000	0.479888000000
O	8.380345000000	4.366656000000	2.807975000000
O	8.566224000000	-0.364672000000	4.570892000000
O	5.135511000000	3.232416000000	5.909871000000
O	-2.204506000000	5.807017000000	-0.316605000000
O	-1.759996000000	5.831995000000	2.438751000000
O	2.365957000000	7.247146000000	1.851130000000
O	2.405306000000	6.891473000000	4.479757000000
H	6.878059000000	2.080908000000	0.251947000000
H	3.788597000000	3.141800000000	-1.680887000000
H	5.380521000000	-0.612751000000	2.697742000000
H	-1.072277000000	1.254522000000	3.768958000000
H	2.407654000000	2.269345000000	3.888486000000
H	-4.395767000000	4.281135000000	0.747214000000
H	-0.800120000000	5.704564000000	2.661500000000
H	2.612550000000	-1.997213000000	0.153418000000
H	1.022619000000	4.257384000000	3.150122000000
H	2.701313000000	6.329492000000	5.223781000000
H	5.884138000000	3.752341000000	6.253315000000
H	4.111430000000	0.668464000000	3.845730000000
H	11.261968000000	2.837075000000	1.542080000000
H	8.541411000000	3.589185000000	-0.149672000000

H	7.415084000000	4.265266000000	2.964590000000
H	5.317378000000	8.837886000000	1.280490000000
H	8.079083000000	6.794413000000	1.235835000000
H	6.737515000000	0.369246000000	1.560579000000
H	9.206116000000	-0.986646000000	4.185400000000
H	-0.790881000000	6.052079000000	-1.416250000000
H	1.744748000000	4.333492000000	-3.130880000000
H	-0.067281000000	1.624076000000	-1.390085000000
H	-2.645452000000	6.675509000000	-0.270747000000
H	3.249877000000	7.704709000000	1.875574000000
H	4.361540000000	6.693898000000	-1.036758000000
H	2.344373000000	6.992339000000	0.180863000000
Ni	-4.516242000000	1.494301000000	4.427806000000
N	-4.858735000000	2.886245000000	5.771164000000
C	-6.402962000000	3.710984000000	-0.824991000000
H	-7.349519000000	4.158172000000	-0.499908000000
H	-5.849134000000	4.238636000000	-1.608061000000
C	-5.958079000000	2.548130000000	-0.310623000000
C	-6.651130000000	1.746398000000	0.765840000000
H	-7.735785000000	1.956421000000	0.762830000000
H	-6.532330000000	0.670071000000	0.543246000000
C	-2.539727000000	2.758542000000	5.787621000000
C	-3.779681000000	3.356656000000	6.360611000000
H	-5.004407000000	2.138590000000	-0.675013000000
O	-2.712099000000	1.790725000000	4.945644000000
C	-3.772033000000	4.251007000000	7.560719000000
H	-2.997192000000	3.933709000000	8.275147000000
H	-3.573945000000	5.298537000000	7.276886000000
H	-4.751496000000	4.228742000000	8.056654000000
C	-6.168128000000	3.189570000000	6.287547000000
C	-6.872639000000	4.334065000000	5.838137000000
C	-6.757505000000	2.265738000000	7.192441000000
C	-8.137408000000	4.586223000000	6.399974000000
C	-8.026732000000	2.567075000000	7.712170000000
C	-8.722453000000	3.736243000000	7.354498000000
H	-8.696553000000	5.460846000000	6.051203000000
H	-8.493399000000	1.852360000000	8.398462000000
C	-10.064203000000	4.060000000000	7.967490000000
H	-10.626999000000	3.145659000000	8.213917000000
H	-9.940573000000	4.629649000000	8.906054000000
H	-10.680084000000	4.674007000000	7.291757000000
C	-6.014589000000	0.982639000000	7.590262000000
H	-5.426951000000	0.677948000000	6.695208000000
C	-6.277685000000	5.257875000000	4.768067000000
H	-5.622935000000	4.626568000000	4.134149000000
C	-6.966179000000	-0.188032000000	7.878295000000
C	-7.021501000000	-0.839369000000	9.123380000000
C	-7.807544000000	-0.650617000000	6.843294000000
C	-7.897654000000	-1.919142000000	9.330655000000
H	-6.374266000000	-0.508419000000	9.940304000000
C	-8.680268000000	-1.727033000000	7.045480000000
H	-7.788393000000	-0.147625000000	5.869351000000

C	-8.728913000000	-2.367212000000	8.295595000000
H	-7.924427000000	-2.410708000000	10.307880000000
H	-9.324417000000	-2.067610000000	6.228755000000
H	-9.408866000000	-3.208659000000	8.457555000000
C	-4.983265000000	1.221768000000	8.695031000000
C	-3.709904000000	0.625054000000	8.601409000000
C	-5.276650000000	2.003846000000	9.829596000000
C	-2.750843000000	0.807537000000	9.610255000000
H	-3.473183000000	0.000037000000	7.732050000000
C	-4.322525000000	2.184230000000	10.843134000000
H	-6.259798000000	2.477279000000	9.921239000000
C	-3.054520000000	1.590391000000	10.735282000000
H	-1.769044000000	0.331973000000	9.521027000000
H	-4.571275000000	2.790117000000	11.719893000000
H	-2.310885000000	1.732136000000	11.525067000000
C	-7.341537000000	5.839463000000	3.817637000000
C	-8.324683000000	4.992944000000	3.258927000000
C	-7.333501000000	7.191249000000	3.426311000000
C	-9.266060000000	5.479703000000	2.341512000000
H	-8.373369000000	3.942961000000	3.567631000000
C	-8.275983000000	7.682783000000	2.507095000000
H	-6.585269000000	7.871775000000	3.841455000000
C	-9.244911000000	6.831266000000	1.959224000000
H	-10.025035000000	4.804831000000	1.932594000000
H	-8.250725000000	8.739586000000	2.224092000000
H	-9.982018000000	7.215864000000	1.248133000000
C	-5.355704000000	6.324712000000	5.366705000000
C	-4.117089000000	6.590691000000	4.749820000000
C	-5.718360000000	7.069225000000	6.506768000000
C	-3.255789000000	7.573753000000	5.265182000000
H	-3.817133000000	6.036705000000	3.853864000000
C	-4.858479000000	8.050846000000	7.024196000000
H	-6.676570000000	6.877581000000	7.000313000000
C	-3.621690000000	8.304827000000	6.407224000000
H	-2.305348000000	7.768965000000	4.758198000000
H	-5.156515000000	8.620196000000	7.910097000000
H	-2.954094000000	9.073114000000	6.809209000000
C	-1.200676000000	3.173544000000	6.169917000000
C	-0.138384000000	2.236245000000	6.283408000000
C	-0.906333000000	4.555182000000	6.349749000000
C	1.167534000000	2.657256000000	6.519111000000
H	-0.351627000000	1.166761000000	6.195826000000
C	0.400005000000	4.982637000000	6.537481000000
H	-1.691849000000	5.306418000000	6.241358000000
C	1.455753000000	4.041747000000	6.571648000000
H	1.977059000000	1.925333000000	6.599623000000
H	0.632655000000	6.048892000000	6.595065000000
O	2.712175000000	4.550049000000	6.623737000000
H	3.428787000000	3.875987000000	6.482399000000
C	-6.400542000000	1.026650000000	3.256033000000
H	-6.265288000000	1.485155000000	4.300691000000
H	-7.494052000000	0.872485000000	3.334681000000

C	-6.073240000000	2.069772000000	2.166193000000
H	-6.462089000000	3.054008000000	2.475407000000
H	-4.978859000000	2.208078000000	2.085393000000
C	-5.697832000000	-0.363529000000	3.152255000000
H	-5.920356000000	-0.826797000000	2.171768000000
H	-6.113885000000	-1.024493000000	3.930655000000
C	-4.212333000000	-0.114677000000	3.363956000000
H	-3.729305000000	-0.773521000000	4.109114000000
C	-3.380573000000	0.058125000000	2.099878000000
H	-4.040566000000	0.328269000000	1.255579000000
H	-2.653728000000	0.882578000000	2.198755000000
C	-2.580047000000	-1.213356000000	1.731295000000
H	-3.266927000000	-2.067591000000	1.583701000000
H	-1.920431000000	-1.464727000000	2.580523000000
C	-1.724560000000	-0.982521000000	0.471679000000
H	-1.189515000000	-0.023914000000	0.581587000000
H	-2.365904000000	-0.895851000000	-0.423742000000
C	-0.700127000000	-2.091259000000	0.267112000000
H	-1.160755000000	-3.057939000000	0.015170000000
H	-0.051304000000	-2.213612000000	1.147438000000
Cl	0.422334000000	-1.697732000000	-1.125262000000

AMA

C	-4.962228000000	0.751016000000	-0.144193000000
H	-4.764026000000	1.798325000000	0.072862000000
H	-5.938601000000	0.508189000000	-0.551903000000
C	-4.047735000000	-0.190366000000	0.084774000000
H	-4.287879000000	-1.228384000000	-0.149345000000
C	-2.672509000000	0.056080000000	0.635097000000
H	-2.557945000000	1.118442000000	0.883348000000
H	-2.552384000000	-0.503980000000	1.572866000000
C	-1.565932000000	-0.369930000000	-0.338099000000
H	-1.669782000000	0.199798000000	-1.270321000000
H	-1.700736000000	-1.427438000000	-0.602563000000
C	-0.165571000000	-0.166596000000	0.237977000000
H	-0.059894000000	-0.744196000000	1.164334000000
H	-0.025759000000	0.889319000000	0.500247000000
C	0.933059000000	-0.593873000000	-0.747977000000
H	0.859644000000	-0.003778000000	-1.666065000000
H	0.818588000000	-1.652453000000	-0.996011000000
C	2.300907000000	-0.415909000000	-0.140107000000
O	2.885515000000	-1.248953000000	0.516202000000
O	2.794569000000	0.812848000000	-0.374037000000
C	4.064505000000	1.096573000000	0.217528000000
H	4.822489000000	0.404177000000	-0.155376000000
H	4.301634000000	2.118504000000	-0.074395000000
H	4.007010000000	1.010346000000	1.304989000000

I$_{AMA}$

O	6.062892000000	1.900967000000	-0.500963000000
Si	4.544905000000	1.950572000000	0.112426000000
O	4.370464000000	0.659552000000	1.109374000000

Si	3.301772000000	-0.466985000000	1.645953000000
O	3.305608000000	-1.834850000000	0.728483000000
O	3.389630000000	1.778182000000	-1.081408000000
O	4.201673000000	3.367192000000	0.899340000000
Si	4.690195000000	4.342484000000	2.112343000000
O	3.431102000000	4.905331000000	2.976327000000
Si	2.270973000000	6.086176000000	3.034351000000
O	0.844839000000	5.273297000000	2.816958000000
O	1.809643000000	0.138126000000	1.818869000000
Si	0.883373000000	1.276159000000	2.568045000000
O	-0.649051000000	0.645423000000	2.685878000000
O	3.934579000000	-0.898633000000	3.150984000000
O	5.718595000000	5.510590000000	1.638475000000
Si	5.933017000000	6.760400000000	0.578609000000
O	5.150048000000	8.028793000000	1.388238000000
O	5.343450000000	6.490557000000	-0.918840000000
O	7.559819000000	7.013058000000	0.395583000000
O	5.629151000000	3.444483000000	3.173872000000
Si	5.396721000000	2.417559000000	4.465572000000
O	4.028471000000	1.511673000000	4.329456000000
O	0.876592000000	2.677580000000	1.661214000000
Si	0.176061000000	3.201610000000	0.214458000000
O	-1.366626000000	3.666092000000	0.516073000000
Si	-2.242957000000	4.945075000000	1.136672000000
O	-3.848529000000	4.551744000000	1.197017000000
O	0.171438000000	1.975184000000	-0.888800000000
O	1.125080000000	4.490111000000	-0.140132000000
Si	1.419153000000	5.619225000000	-1.318972000000
O	2.606809000000	6.622778000000	-0.728318000000
O	2.138367000000	4.833093000000	-2.603076000000
O	0.050750000000	6.383377000000	-1.805170000000
O	1.333931000000	1.623047000000	4.107275000000
O	6.794124000000	1.530520000000	4.511504000000
Si	7.816002000000	0.673922000000	3.543621000000
O	6.933765000000	-0.425235000000	2.639928000000
O	8.749032000000	1.552551000000	2.537680000000
Si	9.058814000000	3.091664000000	2.009895000000
O	10.673691000000	3.342898000000	1.810759000000
O	8.328391000000	3.135818000000	0.501473000000
O	8.463831000000	4.230312000000	3.054677000000
O	8.675523000000	-0.177582000000	4.694539000000
O	5.222043000000	3.257173000000	5.879320000000
O	-2.052551000000	6.131415000000	-0.042934000000
O	-1.807107000000	5.411825000000	2.647413000000
O	2.521780000000	7.245750000000	1.870791000000
O	2.298461000000	6.879246000000	4.479909000000
H	6.821748000000	2.404715000000	-0.115816000000
H	3.380394000000	2.504161000000	-1.737838000000
H	4.885851000000	-1.138283000000	3.060664000000
H	-1.059866000000	0.417920000000	1.832975000000
H	2.306649000000	1.714363000000	4.274351000000
H	-4.135265000000	4.217857000000	2.068008000000

H	-0.836021000000	5.501715000000	2.826083000000
H	3.055253000000	-1.758526000000	-0.208358000000
H	0.968427000000	4.336962000000	2.515818000000
H	2.134937000000	6.343101000000	5.281273000000
H	5.978112000000	3.816727000000	6.128998000000
H	4.092735000000	0.576365000000	3.972293000000
H	11.146096000000	3.789727000000	2.533585000000
H	8.557477000000	3.915891000000	-0.039185000000
H	7.483338000000	4.219254000000	3.131263000000
H	5.247277000000	8.900619000000	0.962578000000
H	8.062817000000	7.090362000000	1.225162000000
H	6.819614000000	-0.189660000000	1.698428000000
H	9.384468000000	-0.765998000000	4.380462000000
H	-0.744068000000	6.315190000000	-1.215587000000
H	1.693842000000	4.962244000000	-3.458446000000
H	1.077818000000	1.676998000000	-1.115047000000
H	-2.608398000000	6.921879000000	0.077613000000
H	3.411425000000	7.690207000000	1.900827000000
H	4.349715000000	6.516773000000	-1.001981000000
H	2.450610000000	6.924464000000	0.209043000000
Ni	-0.580870000000	-0.473994000000	7.030314000000
N	-0.165278000000	0.881333000000	8.405185000000
C	-5.343616000000	2.055475000000	3.289863000000
H	-6.075513000000	2.382014000000	4.037253000000
H	-5.496249000000	2.401444000000	2.262052000000
C	-4.336289000000	1.221123000000	3.606847000000
C	-4.110351000000	0.600134000000	4.959590000000
H	-4.807950000000	1.034088000000	5.699128000000
H	-4.360481000000	-0.478438000000	4.892291000000
C	2.005009000000	0.164816000000	7.850067000000
C	1.115108000000	1.041194000000	8.694514000000
H	-3.630497000000	0.919367000000	2.820060000000
O	1.364583000000	-0.618115000000	7.071395000000
C	1.618924000000	1.978427000000	9.746898000000
H	2.130287000000	1.420642000000	10.549440000000
H	2.327081000000	2.698265000000	9.303685000000
H	0.793982000000	2.541151000000	10.199395000000
C	-1.145538000000	1.548912000000	9.233639000000
C	-1.647981000000	2.828459000000	8.886592000000
C	-1.619103000000	0.861982000000	10.382262000000
C	-2.562752000000	3.435589000000	9.767151000000
C	-2.558814000000	1.504128000000	11.204223000000
C	-3.021451000000	2.804009000000	10.935039000000
H	-2.959218000000	4.422010000000	9.505757000000
H	-2.951537000000	0.960392000000	12.070427000000
C	-3.988489000000	3.494398000000	11.867538000000
H	-3.451132000000	3.998751000000	12.690686000000
H	-4.578367000000	4.261835000000	11.342346000000
H	-4.686568000000	2.776696000000	12.327371000000
C	-1.108620000000	-0.543526000000	10.721051000000
H	-0.845127000000	-1.013773000000	9.752639000000
C	-1.201340000000	3.542213000000	7.602002000000

H	-0.992970000000	2.745645000000	6.860327000000
C	-2.203066000000	-1.433361000000	11.328617000000
C	-2.126811000000	-1.962613000000	12.629020000000
C	-3.329675000000	-1.749217000000	10.538399000000
C	-3.153364000000	-2.781963000000	13.130692000000
H	-1.258737000000	-1.742012000000	13.256847000000
C	-4.352616000000	-2.566814000000	11.035254000000
H	-3.407997000000	-1.337178000000	9.525482000000
C	-4.268124000000	-3.086906000000	12.338218000000
H	-3.074861000000	-3.183582000000	14.145608000000
H	-5.217919000000	-2.798676000000	10.406319000000
H	-5.065483000000	-3.725909000000	12.729013000000
C	0.185648000000	-0.508641000000	11.539727000000
C	1.227620000000	-1.405912000000	11.231498000000
C	0.373345000000	0.386692000000	12.611611000000
C	2.428575000000	-1.413291000000	11.958167000000
H	1.095609000000	-2.119460000000	10.411368000000
C	1.569426000000	0.383416000000	13.347288000000
H	-0.416838000000	1.099210000000	12.868916000000
C	2.602194000000	-0.511964000000	13.021042000000
H	3.215754000000	-2.127642000000	11.694708000000
H	1.693997000000	1.083412000000	14.179492000000
H	3.532586000000	-0.512716000000	13.596960000000
C	-2.301511000000	4.431043000000	6.991450000000
C	-3.615691000000	3.931451000000	6.859802000000
C	-2.033780000000	5.723689000000	6.502968000000
C	-4.628453000000	4.703643000000	6.275899000000
H	-3.856410000000	2.932850000000	7.238600000000
C	-3.046015000000	6.497863000000	5.912811000000
H	-1.025230000000	6.138630000000	6.579205000000
C	-4.348221000000	5.994640000000	5.800192000000
H	-5.641325000000	4.296261000000	6.196403000000
H	-2.808066000000	7.497285000000	5.536878000000
H	-5.138160000000	6.599867000000	5.345490000000
C	0.124810000000	4.281162000000	7.793677000000
C	1.192252000000	4.047640000000	6.905747000000
C	0.306915000000	5.208389000000	8.839801000000
C	2.422304000000	4.711486000000	7.068994000000
H	1.062998000000	3.334572000000	6.082297000000
C	1.523447000000	5.889021000000	8.994587000000
H	-0.513034000000	5.401984000000	9.539033000000
C	2.588485000000	5.638084000000	8.113026000000
H	3.262209000000	4.468265000000	6.409767000000
H	1.641924000000	6.612278000000	9.807277000000
H	3.542980000000	6.158107000000	8.241779000000
C	3.465184000000	0.132626000000	7.824854000000
C	4.073037000000	-0.618514000000	6.774710000000
C	4.336387000000	0.768947000000	8.755574000000
C	5.450639000000	-0.673663000000	6.617959000000
H	3.428906000000	-1.131034000000	6.056293000000
C	5.717205000000	0.707451000000	8.613501000000
H	3.943021000000	1.310979000000	9.612545000000

C	6.294414000000	0.029095000000	7.514859000000
H	5.890549000000	-1.241218000000	5.791647000000
H	6.376923000000	1.206705000000	9.327684000000
O	7.632937000000	0.089147000000	7.381133000000
H	7.930814000000	-0.202940000000	6.483417000000
C	-2.460579000000	-0.051533000000	6.784119000000
H	-2.767236000000	0.532360000000	7.669271000000
H	-3.047290000000	-0.987062000000	6.765230000000
C	-2.644006000000	0.713800000000	5.466722000000
H	-2.387929000000	1.779810000000	5.580845000000
H	-1.980802000000	0.335468000000	4.669124000000
C	-0.987901000000	-2.563754000000	6.935260000000
H	-2.048866000000	-2.621792000000	7.207824000000
H	0.331012000000	-2.113009000000	5.266442000000
C	-0.671809000000	-2.003910000000	5.693763000000
H	-1.468193000000	-1.741606000000	4.991407000000
C	-0.112571000000	-3.436881000000	7.804106000000
H	-0.683772000000	-4.378314000000	7.943020000000
H	-0.077785000000	-3.005920000000	8.823846000000
C	1.306507000000	-3.768412000000	7.323062000000
H	1.829172000000	-2.840635000000	7.036650000000
H	1.251696000000	-4.396767000000	6.414080000000
C	2.118642000000	-4.474856000000	8.418292000000
H	1.625984000000	-5.420232000000	8.712967000000
H	2.139025000000	-3.842952000000	9.323852000000
C	3.573964000000	-4.759826000000	8.015442000000
H	3.997014000000	-3.869328000000	7.509710000000
H	3.657667000000	-5.599027000000	7.306515000000
C	4.460566000000	-5.013285000000	9.223682000000
O	4.386977000000	-4.405758000000	10.287082000000
O	5.377727000000	-5.983796000000	8.974507000000
C	6.296355000000	-6.261575000000	10.062209000000
H	5.742780000000	-6.588841000000	10.955281000000
H	6.947911000000	-7.062653000000	9.691495000000
H	6.881411000000	-5.362262000000	10.308767000000

TS$_{AMA}$

O	6.062892000000	1.900967000000	-0.500963000000
Si	4.544905000000	1.950572000000	0.112426000000
O	4.370464000000	0.659552000000	1.109374000000
Si	3.301772000000	-0.466985000000	1.645953000000
O	3.305608000000	-1.834850000000	0.728483000000
O	3.389630000000	1.778182000000	-1.081408000000
O	4.201673000000	3.367192000000	0.899340000000
Si	4.690195000000	4.342484000000	2.112343000000
O	3.431102000000	4.905331000000	2.976327000000
Si	2.270973000000	6.086176000000	3.034351000000
O	0.844839000000	5.273297000000	2.816958000000
O	1.809643000000	0.138126000000	1.818869000000
Si	0.883373000000	1.276159000000	2.568045000000
O	-0.649051000000	0.645423000000	2.685878000000
O	3.934579000000	-0.898633000000	3.150984000000

O	5.718595000000	5.510590000000	1.638475000000
Si	5.933017000000	6.760400000000	0.578609000000
O	5.150048000000	8.028793000000	1.388238000000
O	5.343450000000	6.490557000000	-0.918840000000
O	7.559819000000	7.013058000000	0.395583000000
O	5.629151000000	3.444483000000	3.173872000000
Si	5.396721000000	2.417559000000	4.465572000000
O	4.028471000000	1.511673000000	4.329456000000
O	0.876592000000	2.677580000000	1.661214000000
Si	0.176061000000	3.201610000000	0.214458000000
O	-1.366626000000	3.666092000000	0.516073000000
Si	-2.242957000000	4.945075000000	1.136672000000
O	-3.848529000000	4.551744000000	1.197017000000
O	0.171438000000	1.975184000000	-0.888800000000
O	1.125080000000	4.490111000000	-0.140132000000
Si	1.419153000000	5.619225000000	-1.318972000000
O	2.606809000000	6.622778000000	-0.728318000000
O	2.138367000000	4.833093000000	-2.603076000000
O	0.050750000000	6.383377000000	-1.805170000000
O	1.333931000000	1.623047000000	4.107275000000
O	6.794124000000	1.530520000000	4.511504000000
Si	7.816002000000	0.673922000000	3.543621000000
O	6.933765000000	-0.425235000000	2.639928000000
O	8.749032000000	1.552551000000	2.537680000000
Si	9.058814000000	3.091664000000	2.009895000000
O	10.673691000000	3.342898000000	1.810759000000
O	8.328391000000	3.135818000000	0.501473000000
O	8.463831000000	4.230312000000	3.054677000000
O	8.675523000000	-0.177582000000	4.694539000000
O	5.222043000000	3.257173000000	5.879320000000
O	-2.052551000000	6.131415000000	-0.042934000000
O	-1.807107000000	5.411825000000	2.647413000000
O	2.521780000000	7.245750000000	1.870791000000
O	2.298461000000	6.879246000000	4.479909000000
H	6.821748000000	2.404715000000	-0.115816000000
H	3.380394000000	2.504161000000	-1.737838000000
H	4.885851000000	-1.138283000000	3.060664000000
H	-1.059866000000	0.417920000000	1.832975000000
H	2.306649000000	1.714363000000	4.274351000000
H	-4.135265000000	4.217857000000	2.068008000000
H	-0.836021000000	5.501715000000	2.826083000000
H	3.055253000000	-1.758526000000	-0.208358000000
H	0.968427000000	4.336962000000	2.515818000000
H	2.134937000000	6.343101000000	5.281273000000
H	5.978112000000	3.816727000000	6.128998000000
H	4.092735000000	0.576365000000	3.972293000000
H	11.146096000000	3.789727000000	2.533585000000
H	8.557477000000	3.915891000000	-0.039185000000
H	7.483338000000	4.219254000000	3.131263000000
H	5.247277000000	8.900619000000	0.962578000000
H	8.062817000000	7.090362000000	1.225162000000
H	6.819614000000	-0.189660000000	1.698428000000

H	9.384468000000	-0.765998000000	4.380462000000
H	-0.744068000000	6.315190000000	-1.215587000000
H	1.693842000000	4.962244000000	-3.458446000000
H	1.077818000000	1.676998000000	-1.115047000000
H	-2.608398000000	6.921879000000	0.077613000000
H	3.411425000000	7.690207000000	1.900827000000
H	4.349715000000	6.516773000000	-1.001981000000
H	2.450610000000	6.924464000000	0.209043000000
Ni	-0.580870000000	-0.473994000000	7.030314000000
N	-0.165278000000	0.881333000000	8.405185000000
C	-5.343616000000	2.055475000000	3.289863000000
H	-6.075513000000	2.382014000000	4.037253000000
H	-5.496249000000	2.401444000000	2.262052000000
C	-4.336289000000	1.221123000000	3.606847000000
C	-4.110351000000	0.600134000000	4.959590000000
H	-4.807950000000	1.034088000000	5.699128000000
H	-4.360481000000	-0.478438000000	4.892291000000
C	2.005009000000	0.164816000000	7.850067000000
C	1.115108000000	1.041194000000	8.694514000000
H	-3.630497000000	0.919367000000	2.820060000000
O	1.364583000000	-0.618115000000	7.071395000000
C	1.618924000000	1.978427000000	9.746898000000
H	2.130287000000	1.420642000000	10.549440000000
H	2.327081000000	2.698265000000	9.303685000000
H	0.793982000000	2.541151000000	10.199395000000
C	-1.145538000000	1.548912000000	9.233639000000
C	-1.647981000000	2.828459000000	8.886592000000
C	-1.619103000000	0.861982000000	10.382262000000
C	-2.562752000000	3.435589000000	9.767151000000
C	-2.558814000000	1.504128000000	11.204223000000
C	-3.021451000000	2.804009000000	10.935039000000
H	-2.959218000000	4.422010000000	9.505757000000
H	-2.951537000000	0.960392000000	12.070427000000
C	-3.988489000000	3.494398000000	11.867538000000
H	-3.451132000000	3.998751000000	12.690686000000
H	-4.578367000000	4.261835000000	11.342346000000
H	-4.686568000000	2.776696000000	12.327371000000
C	-1.108620000000	-0.543526000000	10.721051000000
H	-0.845127000000	-1.013773000000	9.752639000000
C	-1.201340000000	3.542213000000	7.602002000000
H	-0.992970000000	2.745645000000	6.860327000000
C	-2.203066000000	-1.433361000000	11.328617000000
C	-2.126811000000	-1.962613000000	12.629020000000
C	-3.329675000000	-1.749217000000	10.538399000000
C	-3.153364000000	-2.781963000000	13.130692000000
H	-1.258737000000	-1.742012000000	13.256847000000
C	-4.352616000000	-2.566814000000	11.035254000000
H	-3.407997000000	-1.337178000000	9.525482000000
C	-4.268124000000	-3.086906000000	12.338218000000
H	-3.074861000000	-3.183582000000	14.145608000000
H	-5.217919000000	-2.798676000000	10.406319000000
H	-5.065483000000	-3.725909000000	12.729013000000

C	0.185648000000	-0.508641000000	11.539727000000
C	1.227620000000	-1.405912000000	11.231498000000
C	0.373345000000	0.386692000000	12.611611000000
C	2.428575000000	-1.413291000000	11.958167000000
H	1.095609000000	-2.119460000000	10.411368000000
C	1.569426000000	0.383416000000	13.347288000000
H	-0.416838000000	1.099210000000	12.868916000000
C	2.602194000000	-0.511964000000	13.021042000000
H	3.215754000000	-2.127642000000	11.694708000000
H	1.693997000000	1.083412000000	14.179492000000
H	3.532586000000	-0.512716000000	13.596960000000
C	-2.301511000000	4.431043000000	6.991450000000
C	-3.615691000000	3.931451000000	6.859802000000
C	-2.033780000000	5.723689000000	6.502968000000
C	-4.628453000000	4.703643000000	6.275899000000
H	-3.856410000000	2.932850000000	7.238600000000
C	-3.046015000000	6.497863000000	5.912811000000
H	-1.025230000000	6.138630000000	6.579205000000
C	-4.348221000000	5.994640000000	5.800192000000
H	-5.641325000000	4.296261000000	6.196403000000
H	-2.808066000000	7.497285000000	5.536878000000
H	-5.138160000000	6.599867000000	5.345490000000
C	0.124810000000	4.281162000000	7.793677000000
C	1.192252000000	4.047640000000	6.905747000000
C	0.306915000000	5.208389000000	8.839801000000
C	2.422304000000	4.711486000000	7.068994000000
H	1.062998000000	3.334572000000	6.082297000000
C	1.523447000000	5.889021000000	8.994587000000
H	-0.513034000000	5.401984000000	9.539033000000
C	2.588485000000	5.638084000000	8.113026000000
H	3.262209000000	4.468265000000	6.409767000000
H	1.641924000000	6.612278000000	9.807277000000
H	3.542980000000	6.158107000000	8.241779000000
C	3.465184000000	0.132626000000	7.824854000000
C	4.073037000000	-0.618514000000	6.774710000000
C	4.336387000000	0.768947000000	8.755574000000
C	5.450639000000	-0.673663000000	6.617959000000
H	3.428906000000	-1.131034000000	6.056293000000
C	5.717205000000	0.707451000000	8.613501000000
H	3.943021000000	1.310979000000	9.612545000000
C	6.294414000000	0.029095000000	7.514859000000
H	5.890549000000	-1.241218000000	5.791647000000
H	6.376923000000	1.206705000000	9.327684000000
O	7.632937000000	0.089147000000	7.381133000000
H	7.930814000000	-0.202940000000	6.483417000000
C	-2.460579000000	-0.051533000000	6.784119000000
H	-2.767236000000	0.532360000000	7.669271000000
H	-3.047290000000	-0.987062000000	6.765230000000
C	-2.644006000000	0.713800000000	5.466722000000
H	-2.387929000000	1.779810000000	5.580845000000
H	-1.980802000000	0.335468000000	4.669124000000
C	-0.987901000000	-2.563754000000	6.935260000000

H	-2.048866000000	-2.621792000000	7.207824000000
H	0.331012000000	-2.113009000000	5.266442000000
C	-0.671809000000	-2.003910000000	5.693763000000
H	-1.468193000000	-1.741606000000	4.991407000000
C	-0.112571000000	-3.436881000000	7.804106000000
H	-0.683772000000	-4.378314000000	7.943020000000
H	-0.077785000000	-3.005920000000	8.823846000000
C	1.306507000000	-3.768412000000	7.323062000000
H	1.829172000000	-2.840635000000	7.036650000000
H	1.251696000000	-4.396767000000	6.414080000000
C	2.118642000000	-4.474856000000	8.418292000000
H	1.625984000000	-5.420232000000	8.712967000000
H	2.139025000000	-3.842952000000	9.323852000000
C	3.573964000000	-4.759826000000	8.015442000000
H	3.997014000000	-3.869328000000	7.509710000000
H	3.657667000000	-5.599027000000	7.306515000000
C	4.460566000000	-5.013285000000	9.223682000000
O	4.386977000000	-4.405758000000	10.287082000000
O	5.377727000000	-5.983796000000	8.974507000000
C	6.296355000000	-6.261575000000	10.062209000000
H	5.742780000000	-6.588841000000	10.955281000000
H	6.947911000000	-7.062653000000	9.691495000000
H	6.881411000000	-5.362262000000	10.308767000000

P$_{AMA}$

O	6.066535000000	1.844197000000	-0.488927000000
Si	4.544459000000	1.949737000000	0.112792000000
O	4.334328000000	0.731946000000	1.192013000000
Si	3.301829000000	-0.465415000000	1.645161000000
O	3.416624000000	-1.685477000000	0.579738000000
O	3.382841000000	1.789375000000	-1.071304000000
O	4.248524000000	3.408663000000	0.853777000000
Si	4.690380000000	4.342091000000	2.112655000000
O	3.413438000000	4.886902000000	2.968835000000
Si	2.270953000000	6.086134000000	3.034250000000
O	0.837375000000	5.281298000000	2.819064000000
O	1.781065000000	0.086923000000	1.861039000000
Si	0.883729000000	1.275818000000	2.568842000000
O	-0.668862000000	0.693264000000	2.716566000000
O	3.912520000000	-0.879956000000	3.183472000000
O	5.703660000000	5.547369000000	1.681267000000
Si	5.933058000000	6.760292000000	0.578643000000
O	5.142982000000	8.051099000000	1.345770000000
O	5.355680000000	6.454464000000	-0.917150000000
O	7.562361000000	7.003772000000	0.400949000000
O	5.622289000000	3.434354000000	3.166825000000
Si	5.396460000000	2.417740000000	4.465672000000
O	4.039193000000	1.492808000000	4.378522000000
O	0.900046000000	2.661149000000	1.638749000000
Si	0.175901000000	3.201526000000	0.213627000000
O	-1.367726000000	3.649482000000	0.559115000000
Si	-2.242879000000	4.945044000000	1.136723000000

O	-3.844888000000	4.535022000000	1.257942000000
O	0.149420000000	1.997478000000	-0.910374000000
O	1.097598000000	4.511009000000	-0.129406000000
Si	1.419175000000	5.619220000000	-1.318772000000
O	2.612818000000	6.616614000000	-0.731482000000
O	2.140018000000	4.824022000000	-2.595655000000
O	0.058229000000	6.394014000000	-1.814600000000
O	1.346749000000	1.648167000000	4.100231000000
O	6.801061000000	1.537051000000	4.511635000000
Si	7.816168000000	0.674217000000	3.543468000000
O	6.947287000000	-0.438378000000	2.649667000000
O	8.750362000000	1.553288000000	2.538024000000
Si	9.058793000000	3.091455000000	2.009914000000
O	10.675146000000	3.342780000000	1.815650000000
O	8.326885000000	3.136103000000	0.503156000000
O	8.465572000000	4.233714000000	3.053417000000
O	8.693526000000	-0.169531000000	4.691490000000
O	5.228350000000	3.279380000000	5.869311000000
O	-2.090178000000	6.074589000000	-0.103404000000
O	-1.792326000000	5.498189000000	2.614441000000
O	2.526248000000	7.245103000000	1.871725000000
O	2.302048000000	6.874529000000	4.482466000000
H	6.819563000000	2.366969000000	-0.120996000000
H	3.389398000000	2.507206000000	-1.736002000000
H	4.850004000000	-1.165429000000	3.097319000000
H	-1.129720000000	0.549322000000	1.870753000000
H	2.321873000000	1.713013000000	4.268491000000
H	-4.135878000000	4.404623000000	2.178256000000
H	-0.816132000000	5.553372000000	2.791126000000
H	2.782992000000	-2.450033000000	0.553557000000
H	0.963629000000	4.344497000000	2.522745000000
H	2.113465000000	6.337751000000	5.278017000000
H	5.952521000000	3.898786000000	6.066343000000
H	4.092134000000	0.567600000000	3.980716000000
H	11.140725000000	3.798009000000	2.537620000000
H	8.542016000000	3.923341000000	-0.032695000000
H	7.485029000000	4.227108000000	3.124453000000
H	5.223819000000	8.906810000000	0.885598000000
H	8.060520000000	7.081613000000	1.233424000000
H	6.799927000000	-0.197390000000	1.713724000000
H	9.403203000000	-0.752465000000	4.368895000000
H	-0.746535000000	6.298037000000	-1.243733000000
H	1.668205000000	4.909338000000	-3.441845000000
H	1.044326000000	1.654298000000	-1.119907000000
H	-2.665287000000	6.855759000000	-0.017436000000
H	3.415226000000	7.691425000000	1.895535000000
H	4.363051000000	6.482836000000	-1.005790000000
H	2.459457000000	6.917807000000	0.206022000000
Ni	-0.243210000000	-0.680912000000	7.455841000000
N	0.114912000000	0.779399000000	8.723290000000
C	-5.286304000000	0.976097000000	3.370149000000
H	-6.183211000000	0.569052000000	3.851530000000

H	-5.443195000000	1.721251000000	2.584238000000
C	-4.052284000000	0.567170000000	3.712153000000
C	-3.743463000000	-0.479949000000	4.752008000000
H	-4.667957000000	-0.747214000000	5.298742000000
H	-3.405659000000	-1.401456000000	4.237942000000
C	2.259620000000	0.118258000000	8.101916000000
C	1.394783000000	1.011014000000	8.943780000000
H	-3.180015000000	1.007251000000	3.210278000000
O	1.607485000000	-0.731778000000	7.383040000000
C	1.901460000000	2.047754000000	9.896734000000
H	2.452668000000	1.585855000000	10.732728000000
H	2.568625000000	2.752235000000	9.372829000000
H	1.064442000000	2.617999000000	10.319957000000
C	-0.893557000000	1.411608000000	9.533568000000
C	-1.531930000000	2.601538000000	9.102146000000
C	-1.284547000000	0.759840000000	10.734197000000
C	-2.472671000000	3.190567000000	9.966949000000
C	-2.257012000000	1.375834000000	11.537647000000
C	-2.835225000000	2.611410000000	11.194993000000
H	-2.967267000000	4.113953000000	9.648492000000
H	-2.585051000000	0.862318000000	12.447975000000
C	-3.829537000000	3.285256000000	12.110861000000
H	-4.418291000000	2.547957000000	12.679534000000
H	-3.314533000000	3.928809000000	12.846669000000
H	-4.527685000000	3.925693000000	11.549164000000
C	-0.669655000000	-0.592775000000	11.124000000000
H	-0.430981000000	-1.108738000000	10.170865000000
C	-1.193779000000	3.225808000000	7.740794000000
H	-0.937041000000	2.380716000000	7.068811000000
C	-1.674187000000	-1.502100000000	11.846336000000
C	-1.588980000000	-1.802932000000	13.217547000000
C	-2.734496000000	-2.061074000000	11.102227000000
C	-2.543689000000	-2.632802000000	13.830459000000
H	-0.766568000000	-1.397697000000	13.814207000000
C	-3.686643000000	-2.890006000000	11.708851000000
H	-2.814347000000	-1.838094000000	10.031509000000
C	-3.595245000000	-3.177696000000	13.081103000000
H	-2.457886000000	-2.855724000000	14.898362000000
H	-4.499385000000	-3.315338000000	11.111776000000
H	-4.335455000000	-3.826522000000	13.558395000000
C	0.661365000000	-0.446683000000	11.863666000000
C	1.722960000000	-1.323402000000	11.560962000000
C	0.860672000000	0.530091000000	12.859482000000
C	2.954414000000	-1.223947000000	12.226906000000
H	1.579818000000	-2.096265000000	10.796720000000
C	2.088029000000	0.627641000000	13.534319000000
H	0.052847000000	1.227915000000	13.102697000000
C	3.141324000000	-0.245780000000	13.217173000000
H	3.766021000000	-1.913728000000	11.975335000000
H	2.221461000000	1.389237000000	14.308951000000
H	4.098200000000	-0.168665000000	13.742126000000
C	-2.383642000000	3.949265000000	7.081983000000

C	-3.672209000000	3.370918000000	7.106093000000
C	-2.214694000000	5.153878000000	6.373804000000
C	-4.753593000000	3.980678000000	6.456262000000
H	-3.840796000000	2.441995000000	7.660558000000
C	-3.295540000000	5.767593000000	5.719770000000
H	-1.228426000000	5.622772000000	6.323628000000
C	-4.570046000000	5.186728000000	5.760566000000
H	-5.741838000000	3.511916000000	6.494861000000
H	-3.130374000000	6.697663000000	5.168927000000
H	-5.415431000000	5.669871000000	5.260707000000
C	0.069265000000	4.088355000000	7.818025000000
C	1.135899000000	3.855214000000	6.929988000000
C	0.191805000000	5.125513000000	8.765636000000
C	2.313043000000	4.623229000000	7.002986000000
H	1.048428000000	3.065760000000	6.173772000000
C	1.352449000000	5.909815000000	8.827011000000
H	-0.630423000000	5.322501000000	9.461272000000
C	2.422383000000	5.655066000000	7.951445000000
H	3.158545000000	4.382853000000	6.349480000000
H	1.425616000000	6.715976000000	9.563436000000
H	3.336635000000	6.254176000000	8.012278000000
C	3.709437000000	0.118235000000	7.997688000000
C	4.292558000000	-0.639181000000	6.937164000000
C	4.597476000000	0.806167000000	8.875296000000
C	5.661466000000	-0.649419000000	6.718958000000
H	3.634714000000	-1.196640000000	6.266729000000
C	5.971023000000	0.785978000000	8.673213000000
H	4.220157000000	1.352454000000	9.737092000000
C	6.520261000000	0.101233000000	7.563101000000
H	6.084310000000	-1.219673000000	5.886206000000
H	6.645744000000	1.322893000000	9.344779000000
O	7.846702000000	0.198676000000	7.368777000000
H	8.112449000000	-0.107413000000	6.462824000000
C	-2.456210000000	-1.009598000000	6.909857000000
H	-1.906049000000	-0.494731000000	7.787787000000
H	-3.414411000000	-1.131202000000	7.452172000000
C	-2.650807000000	-0.022142000000	5.743292000000
H	-2.937764000000	0.960566000000	6.153443000000
H	-1.707804000000	0.139613000000	5.190696000000
C	-1.902727000000	-2.425679000000	6.558462000000
H	-2.401383000000	-2.792607000000	5.641972000000
H	-2.167487000000	-3.123833000000	7.369850000000
C	-0.392992000000	-2.304831000000	6.424413000000
H	0.169648000000	-3.007596000000	7.069138000000
C	0.203723000000	-2.215653000000	5.029501000000
H	1.247819000000	-1.859415000000	5.088137000000
H	-0.346865000000	-1.481522000000	4.413785000000
C	0.186225000000	-3.583267000000	4.302680000000
H	-0.851885000000	-3.964859000000	4.256237000000
H	0.760137000000	-4.320787000000	4.896079000000
C	0.762110000000	-3.496499000000	2.881463000000
H	1.811206000000	-3.154498000000	2.932268000000

H	0.213415000000	-2.729699000000	2.305216000000
C	0.693171000000	-4.837463000000	2.143542000000
H	1.277604000000	-5.610908000000	2.678450000000
H	-0.339991000000	-5.226402000000	2.107349000000
C	1.220279000000	-4.788164000000	0.724383000000
O	1.892269000000	-3.880490000000	0.227863000000
O	0.874391000000	-5.890224000000	0.031252000000
C	1.363524000000	-5.962535000000	-1.336075000000
H	2.463354000000	-5.937501000000	-1.347989000000
H	0.988174000000	-6.918048000000	-1.721666000000
H	0.971517000000	-5.119424000000	-1.924001000000

NBA

C	2.747620000000	0.717407000000	-0.078797000000
C	1.906697000000	0.196188000000	1.077068000000
C	1.146628000000	-0.775424000000	-0.800601000000
C	2.291579000000	0.142068000000	-1.196721000000
H	3.504505000000	1.489495000000	0.000737000000
H	2.596346000000	0.348722000000	-2.216059000000
C	1.624633000000	-1.239630000000	0.590008000000
H	0.847282000000	-1.752950000000	1.163567000000
H	2.524418000000	-1.857122000000	0.538665000000
C	0.495883000000	0.833649000000	0.902733000000
H	-0.158894000000	0.563545000000	1.737004000000
H	0.538028000000	1.923357000000	0.833278000000
C	-0.022905000000	0.164294000000	-0.401718000000
H	-0.249179000000	0.879071000000	-1.196735000000
H	2.329316000000	0.313262000000	2.075633000000
H	0.851355000000	-1.537721000000	-1.521497000000
O	-1.166606000000	-0.668970000000	-0.165464000000
C	-2.409260000000	-0.161188000000	-0.029403000000
O	-3.315388000000	-0.911510000000	0.237878000000
C	-2.604959000000	1.323409000000	-0.243707000000
H	-1.945240000000	1.915547000000	0.395442000000
H	-2.394287000000	1.592597000000	-1.283369000000
H	-3.643201000000	1.561395000000	-0.019148000000

I_{NBA}

O	6.070978000000	1.859097000000	-0.479261000000
Si	4.544968000000	1.950605000000	0.112429000000
O	4.326531000000	0.700457000000	1.150704000000
Si	3.301788000000	-0.467080000000	1.645962000000
O	3.279659000000	-1.802333000000	0.676712000000
O	3.393263000000	1.760475000000	-1.083610000000
O	4.234283000000	3.393969000000	0.860162000000
Si	4.690097000000	4.342524000000	2.112273000000
O	3.387456000000	4.870971000000	2.939566000000
Si	2.270978000000	6.086176000000	3.034363000000
O	0.805811000000	5.330623000000	2.836350000000
O	1.793866000000	0.077013000000	1.889314000000
Si	0.883263000000	1.276243000000	2.567923000000
O	-0.688645000000	0.744470000000	2.580151000000

O	4.001982000000	-0.992511000000	3.100389000000
O	5.693898000000	5.546490000000	1.673481000000
Si	5.933030000000	6.760384000000	0.578643000000
O	5.158319000000	8.054296000000	1.353342000000
O	5.343249000000	6.462951000000	-0.915199000000
O	7.564186000000	6.984143000000	0.391368000000
O	5.613474000000	3.438958000000	3.167012000000
Si	5.396712000000	2.417755000000	4.465543000000
O	4.038125000000	1.480978000000	4.340553000000
O	0.861913000000	2.664602000000	1.652870000000
Si	0.176087000000	3.201567000000	0.214674000000
O	-1.373723000000	3.686632000000	0.465418000000
Si	-2.242931000000	4.945042000000	1.136656000000
O	-3.843910000000	4.551462000000	1.090435000000
O	0.172071000000	2.010549000000	-0.928721000000
O	1.133778000000	4.492932000000	-0.131954000000
Si	1.419158000000	5.619247000000	-1.319026000000
O	2.588775000000	6.643196000000	-0.727575000000
O	2.145940000000	4.835201000000	-2.600515000000
O	0.038305000000	6.348834000000	-1.832166000000
O	1.359905000000	1.647889000000	4.094174000000
O	6.789633000000	1.529486000000	4.515255000000
Si	7.816028000000	0.673470000000	3.543730000000
O	6.875718000000	-0.439284000000	2.684706000000
O	8.609749000000	1.557022000000	2.416052000000
Si	9.058851000000	3.091926000000	2.009806000000
O	10.689240000000	3.255498000000	1.840694000000
O	8.329969000000	3.260770000000	0.505429000000
O	8.524344000000	4.217749000000	3.105362000000
O	8.823152000000	-0.079514000000	4.605817000000
O	5.164540000000	3.264495000000	5.861756000000
O	-1.870400000000	6.254690000000	0.130660000000
O	-1.921934000000	5.274742000000	2.712246000000
O	2.523867000000	7.255976000000	1.880171000000
O	2.357023000000	6.869596000000	4.486364000000
H	6.812909000000	2.410395000000	-0.126997000000
H	3.401717000000	2.471336000000	-1.756195000000
H	4.987709000000	-1.078581000000	3.003373000000
H	-0.797798000000	-0.162448000000	2.922664000000
H	2.331943000000	1.723510000000	4.279738000000
H	-4.118713000000	4.063676000000	0.284261000000
H	-0.975429000000	5.486985000000	2.902929000000
H	2.961565000000	-1.692986000000	-0.236334000000
H	0.916492000000	4.389078000000	2.554893000000
H	2.218361000000	6.325356000000	5.280804000000
H	5.882449000000	3.859062000000	6.138395000000
H	4.138389000000	0.546606000000	4.027244000000
H	11.174865000000	3.621863000000	2.599383000000
H	8.533787000000	4.088177000000	0.029706000000
H	7.546455000000	4.229149000000	3.193560000000
H	5.251996000000	8.914212000000	0.903765000000
H	8.065195000000	7.087441000000	1.219600000000

H	6.904079000000	-0.289416000000	1.719895000000
H	9.518902000000	-0.645104000000	4.229873000000
H	-0.706964000000	6.366727000000	-1.177657000000
H	1.728426000000	4.995242000000	-3.464053000000
H	1.078455000000	1.712696000000	-1.153262000000
H	-2.262113000000	7.101992000000	0.408833000000
H	3.412268000000	7.701684000000	1.908701000000
H	4.351202000000	6.501947000000	-0.998361000000
H	2.442310000000	6.930320000000	0.215993000000
Ni	-4.971506000000	-1.429704000000	2.462139000000
N	-5.051778000000	-0.832705000000	4.361850000000
C	-4.533326000000	3.026914000000	-1.719138000000
H	-5.555716000000	3.357298000000	-1.937395000000
H	-3.718252000000	3.596421000000	-2.176865000000
C	-4.287337000000	1.941491000000	-0.958442000000
C	-5.347857000000	1.064167000000	-0.348769000000
H	-6.348024000000	1.500431000000	-0.522066000000
H	-5.340715000000	0.089431000000	-0.877667000000
C	-2.872600000000	-1.680208000000	4.272727000000
C	-4.004302000000	-1.142284000000	5.102949000000
H	-3.243545000000	1.648517000000	-0.774592000000
O	-3.199134000000	-2.005527000000	3.078884000000
C	-3.972963000000	-1.055841000000	6.597482000000
H	-3.474751000000	-1.940791000000	7.022978000000
H	-3.440609000000	-0.148224000000	6.931298000000
H	-4.992291000000	-0.999070000000	7.000612000000
C	-6.231304000000	-0.311656000000	5.013906000000
C	-6.360776000000	1.087150000000	5.219562000000
C	-7.260510000000	-1.209078000000	5.397539000000
C	-7.508305000000	1.549292000000	5.888086000000
C	-8.389558000000	-0.685839000000	6.050456000000
C	-8.524346000000	0.684909000000	6.330409000000
H	-7.623302000000	2.628470000000	6.033840000000
H	-9.198105000000	-1.371651000000	6.325871000000
C	-9.726534000000	1.214430000000	7.075721000000
H	-9.532143000000	1.248976000000	8.162789000000
H	-9.979611000000	2.238208000000	6.757717000000
H	-10.610777000000	0.575926000000	6.922036000000
C	-7.126343000000	-2.712021000000	5.132896000000
H	-6.474528000000	-2.804828000000	4.240677000000
C	-5.293193000000	2.071791000000	4.720402000000
H	-4.850787000000	1.614720000000	3.814561000000
C	-8.456629000000	-3.374839000000	4.742054000000
C	-8.897793000000	-4.580940000000	5.317124000000
C	-9.243462000000	-2.793547000000	3.722708000000
C	-10.091743000000	-5.187861000000	4.890028000000
H	-8.308266000000	-5.053827000000	6.107573000000
C	-10.433740000000	-3.396528000000	3.295258000000
H	-8.929405000000	-1.843362000000	3.276113000000
C	-10.863668000000	-4.600504000000	3.878646000000
H	-10.416801000000	-6.123209000000	5.355808000000
H	-11.031058000000	-2.922854000000	2.509668000000

H	-11.794654000000	-5.071372000000	3.549500000000
C	-6.386269000000	-3.430982000000	6.265491000000
C	-5.341121000000	-4.325641000000	5.961153000000
C	-6.727657000000	-3.237547000000	7.619127000000
C	-4.649582000000	-5.005935000000	6.976783000000
H	-5.069944000000	-4.498764000000	4.913534000000
C	-6.041476000000	-3.916658000000	8.637422000000
H	-7.535175000000	-2.546722000000	7.881523000000
C	-4.997078000000	-4.801072000000	8.320828000000
H	-3.843664000000	-5.699299000000	6.716307000000
H	-6.324723000000	-3.755152000000	9.682104000000
H	-4.462772000000	-5.330015000000	9.115568000000
C	-5.890269000000	3.418441000000	4.271607000000
C	-6.962844000000	3.430699000000	3.352974000000
C	-5.373468000000	4.651924000000	4.705604000000
C	-7.508111000000	4.635949000000	2.892584000000
H	-7.394301000000	2.483966000000	3.009155000000
C	-5.918967000000	5.862248000000	4.246017000000
H	-4.529654000000	4.675990000000	5.399768000000
C	-6.987820000000	5.861105000000	3.340956000000
H	-8.343353000000	4.618764000000	2.185171000000
H	-5.497257000000	6.808559000000	4.598228000000
H	-7.412787000000	6.804238000000	2.984632000000
C	-4.127056000000	2.223638000000	5.700524000000
C	-2.804113000000	2.183920000000	5.216750000000
C	-4.336120000000	2.422631000000	7.080016000000
C	-1.711462000000	2.323884000000	6.088519000000
H	-2.618226000000	2.052369000000	4.144765000000
C	-3.247956000000	2.567970000000	7.954916000000
H	-5.356501000000	2.457783000000	7.475307000000
C	-1.932259000000	2.512873000000	7.462546000000
H	-0.693328000000	2.282332000000	5.687254000000
H	-3.428242000000	2.725035000000	9.023050000000
H	-1.085166000000	2.625854000000	8.146516000000
C	-1.480700000000	-1.820398000000	4.677155000000
C	-0.587552000000	-2.427067000000	3.740180000000
C	-0.922677000000	-1.306154000000	5.884148000000
C	0.781567000000	-2.460628000000	3.961865000000
H	-1.001185000000	-2.860209000000	2.825748000000
C	0.443537000000	-1.354981000000	6.121904000000
H	-1.548214000000	-0.808296000000	6.622255000000
C	1.317645000000	-1.885871000000	5.145082000000
H	1.448936000000	-2.921954000000	3.226983000000
H	0.872729000000	-0.920528000000	7.027925000000
O	2.637335000000	-1.815579000000	5.388828000000
H	3.155552000000	-1.884045000000	4.547011000000
C	-6.208400000000	-0.137793000000	1.755641000000
H	-6.786613000000	0.261981000000	2.602862000000
H	-6.890122000000	-0.549246000000	0.993561000000
C	-5.159843000000	0.812222000000	1.166239000000
H	-5.163759000000	1.774907000000	1.708308000000
H	-4.121970000000	0.418913000000	1.322153000000

C	-6.130577000000	-2.940647000000	1.600500000000
C	-5.894238000000	-4.413638000000	1.923690000000
C	-4.173407000000	-3.768136000000	0.590386000000
C	-5.060653000000	-2.536561000000	0.765387000000
H	-7.152246000000	-2.552241000000	1.562918000000
H	-5.163526000000	-1.805715000000	-0.046139000000
C	-4.356311000000	-4.529310000000	1.925117000000
H	-4.006848000000	-5.571239000000	1.856744000000
H	-3.871296000000	-4.032834000000	2.774930000000
C	-6.219348000000	-5.112552000000	0.552673000000
H	-6.215385000000	-6.207024000000	0.685737000000
H	-7.200139000000	-4.818996000000	0.145533000000
C	-5.020053000000	-4.686932000000	-0.357290000000
H	-5.331723000000	-4.157141000000	-1.272425000000
H	-6.450795000000	-4.828346000000	2.775960000000
H	-3.150874000000	-3.601693000000	0.225362000000
O	-4.169668000000	-5.805017000000	-0.701972000000
C	-4.433023000000	-6.623983000000	-1.783262000000
O	-3.683159000000	-7.563194000000	-1.974070000000
C	-5.619743000000	-6.291466000000	-2.665899000000
H	-6.554631000000	-6.203921000000	-2.088836000000
H	-5.463023000000	-5.336605000000	-3.196543000000
H	-5.724206000000	-7.091868000000	-3.408899000000

TS_{NBA}

O	6.069328000000	1.884369000000	-0.485283000000
Si	4.544968000000	1.950605000000	0.112429000000
O	4.343974000000	0.676252000000	1.126097000000
Si	3.301788000000	-0.467080000000	1.645962000000
O	3.268011000000	-1.813021000000	0.686783000000
O	3.394586000000	1.765506000000	-1.087385000000
O	4.213636000000	3.380464000000	0.877290000000
Si	4.690097000000	4.342524000000	2.112273000000
O	3.403576000000	4.887009000000	2.950576000000
Si	2.270978000000	6.086176000000	3.034363000000
O	0.818419000000	5.310314000000	2.829219000000
O	1.801378000000	0.095209000000	1.871215000000
Si	0.883263000000	1.276243000000	2.567923000000
O	-0.687162000000	0.748016000000	2.566058000000
O	3.993215000000	-0.988934000000	3.105476000000
O	5.703219000000	5.529742000000	1.654211000000
Si	5.933030000000	6.760384000000	0.578643000000
O	5.156352000000	8.042948000000	1.370836000000
O	5.340056000000	6.480180000000	-0.917362000000
O	7.562454000000	6.994669000000	0.390529000000
O	5.616125000000	3.439339000000	3.166609000000
Si	5.396712000000	2.417755000000	4.465543000000
O	4.037556000000	1.482745000000	4.335107000000
O	0.860854000000	2.669325000000	1.655929000000
Si	0.176087000000	3.201567000000	0.214674000000
O	-1.375483000000	3.686827000000	0.458313000000
Si	-2.242931000000	4.945042000000	1.136656000000

O	-3.843136000000	4.556450000000	1.150820000000
O	0.190890000000	2.008759000000	-0.928301000000
O	1.133760000000	4.494346000000	-0.132273000000
Si	1.419158000000	5.619247000000	-1.319026000000
O	2.585797000000	6.647418000000	-0.728890000000
O	2.148045000000	4.831777000000	-2.597937000000
O	0.038532000000	6.348881000000	-1.833994000000
O	1.353394000000	1.633205000000	4.098036000000
O	6.790375000000	1.529709000000	4.515792000000
Si	7.816028000000	0.673470000000	3.543730000000
O	6.875311000000	-0.440915000000	2.687046000000
O	8.606497000000	1.557234000000	2.413483000000
Si	9.058851000000	3.091926000000	2.009806000000
O	10.689779000000	3.255225000000	1.845237000000
O	8.335796000000	3.260532000000	0.502313000000
O	8.521218000000	4.217759000000	3.103378000000
O	8.825588000000	-0.078657000000	4.603915000000
O	5.163810000000	3.263270000000	5.861736000000
O	-1.909174000000	6.240971000000	0.095518000000
O	-1.888378000000	5.288871000000	2.700124000000
O	2.520085000000	7.254958000000	1.877964000000
O	2.335538000000	6.874680000000	4.484006000000
H	6.813789000000	2.426579000000	-0.123793000000
H	3.396597000000	2.487909000000	-1.747916000000
H	4.978672000000	-1.077540000000	3.009262000000
H	-0.822116000000	-0.130172000000	2.968786000000
H	2.323265000000	1.712773000000	4.290085000000
H	-4.255873000000	4.352861000000	0.284562000000
H	-0.931598000000	5.459926000000	2.885538000000
H	3.022856000000	-1.686730000000	-0.246331000000
H	0.936941000000	4.369419000000	2.545730000000
H	2.184438000000	6.334153000000	5.278780000000
H	5.881771000000	3.855992000000	6.142029000000
H	4.134447000000	0.544617000000	4.031683000000
H	11.173118000000	3.621346000000	2.605530000000
H	8.543926000000	4.086414000000	0.025970000000
H	7.543254000000	4.223922000000	3.193777000000
H	5.257923000000	8.910385000000	0.937678000000
H	8.065260000000	7.087066000000	1.218975000000
H	6.891923000000	-0.283260000000	1.723252000000
H	9.522272000000	-0.642377000000	4.226953000000
H	-0.713564000000	6.354952000000	-1.186943000000
H	1.748400000000	5.009555000000	-3.466360000000
H	1.104924000000	1.720610000000	-1.136522000000
H	-2.249773000000	7.098657000000	0.407996000000
H	3.409563000000	7.699121000000	1.906821000000
H	4.347745000000	6.517086000000	-0.998438000000
H	2.438531000000	6.933644000000	0.215106000000
Ni	-4.512808000000	-1.514103000000	1.408287000000
N	-4.984361000000	-1.096147000000	3.343897000000
C	-5.336702000000	4.213915000000	-1.724981000000
H	-6.365730000000	4.511971000000	-1.493774000000

H	-4.672407000000	4.988610000000	-2.121150000000
C	-4.916122000000	2.944021000000	-1.560201000000
C	-5.769307000000	1.799404000000	-1.082119000000
H	-6.794897000000	2.150912000000	-0.867883000000
H	-5.858469000000	1.071522000000	-1.915491000000
C	-2.819854000000	-1.932046000000	3.515520000000
C	-4.059829000000	-1.464396000000	4.208646000000
H	-3.873682000000	2.693587000000	-1.807722000000
O	-2.971419000000	-2.170364000000	2.262983000000
C	-4.247479000000	-1.520709000000	5.692894000000
H	-3.786660000000	-2.431122000000	6.106862000000
H	-3.799126000000	-0.637316000000	6.180224000000
H	-5.315689000000	-1.519952000000	5.945402000000
C	-6.281776000000	-0.713022000000	3.845589000000
C	-6.548141000000	0.637080000000	4.192161000000
C	-7.287715000000	-1.709885000000	3.954559000000
C	-7.808651000000	0.936957000000	4.740201000000
C	-8.533594000000	-1.346274000000	4.491019000000
C	-8.806340000000	-0.035875000000	4.920944000000
H	-8.026186000000	1.977754000000	5.001307000000
H	-9.318466000000	-2.107815000000	4.553689000000
C	-10.134429000000	0.317812000000	5.547121000000
H	-10.105842000000	0.167677000000	6.641366000000
H	-10.396712000000	1.372607000000	5.369321000000
H	-10.947493000000	-0.312380000000	5.153110000000
C	-7.004166000000	-3.150412000000	3.516682000000
H	-6.240203000000	-3.074022000000	2.713917000000
C	-5.503656000000	1.740903000000	3.972672000000
H	-4.906048000000	1.432928000000	3.092067000000
C	-8.223560000000	-3.829565000000	2.875498000000
C	-8.685544000000	-5.095731000000	3.277675000000
C	-8.881255000000	-3.185994000000	1.803884000000
C	-9.778161000000	-5.700545000000	2.632417000000
H	-8.190833000000	-5.619371000000	4.100349000000
C	-9.969991000000	-3.786653000000	1.159017000000
H	-8.544837000000	-2.193040000000	1.483890000000
C	-10.424097000000	-5.050698000000	1.572355000000
H	-10.121459000000	-6.685294000000	2.963621000000
H	-10.468617000000	-3.266849000000	0.334688000000
H	-11.274709000000	-5.521850000000	1.071164000000
C	-6.342139000000	-3.978051000000	4.621420000000
C	-5.219416000000	-4.773725000000	4.317749000000
C	-6.832840000000	-3.985997000000	5.942186000000
C	-4.596553000000	-5.552983000000	5.305944000000
H	-4.834158000000	-4.790489000000	3.291365000000
C	-6.215858000000	-4.766183000000	6.932129000000
H	-7.703925000000	-3.375234000000	6.200623000000
C	-5.092749000000	-5.549466000000	6.618889000000
H	-3.728318000000	-6.167276000000	5.047664000000
H	-6.614883000000	-4.763089000000	7.951178000000
H	-4.612273000000	-6.157209000000	7.391351000000
C	-6.144574000000	3.092603000000	3.607403000000

C	-7.127408000000	3.146636000000	2.594435000000
C	-5.750018000000	4.298086000000	4.213805000000
C	-7.704402000000	4.363940000000	2.209200000000
H	-7.467515000000	2.219403000000	2.119560000000
C	-6.326586000000	5.519913000000	3.829639000000
H	-4.977285000000	4.291825000000	4.986754000000
C	-7.306414000000	5.559772000000	2.829449000000
H	-8.474156000000	4.377794000000	1.430518000000
H	-6.000260000000	6.443751000000	4.316823000000
H	-7.758558000000	6.511560000000	2.534676000000
C	-4.497258000000	1.836511000000	5.122459000000
C	-3.120249000000	1.912321000000	4.833955000000
C	-4.908547000000	1.869654000000	6.470064000000
C	-2.171299000000	2.005327000000	5.865597000000
H	-2.778748000000	1.906868000000	3.792867000000
C	-3.964128000000	1.966341000000	7.504139000000
H	-5.974298000000	1.813726000000	6.714686000000
C	-2.591848000000	2.029024000000	7.205283000000
H	-1.106782000000	2.061772000000	5.615064000000
H	-4.300869000000	1.994184000000	8.545236000000
H	-1.857081000000	2.106309000000	8.012825000000
C	-1.489832000000	-2.092218000000	4.087036000000
C	-0.469316000000	-2.625254000000	3.240740000000
C	-1.103730000000	-1.638026000000	5.383231000000
C	0.861449000000	-2.636394000000	3.630645000000
H	-0.750388000000	-3.005691000000	2.256007000000
C	0.225851000000	-1.657986000000	5.784277000000
H	-1.834255000000	-1.203072000000	6.063317000000
C	1.229932000000	-2.105537000000	4.894609000000
H	1.628846000000	-3.035058000000	2.959518000000
H	0.525280000000	-1.261101000000	6.757239000000
O	2.511263000000	-1.995126000000	5.291473000000
H	3.114740000000	-1.995774000000	4.507206000000
C	-5.908216000000	-0.262828000000	0.448445000000
H	-6.361722000000	-0.331697000000	1.454901000000
H	-6.755439000000	-0.434506000000	-0.232595000000
C	-5.197522000000	1.074138000000	0.160549000000
H	-5.276258000000	1.746938000000	1.029693000000
H	-4.110649000000	0.916517000000	0.017707000000
C	-4.991005000000	-1.600105000000	-0.742646000000
C	-5.944301000000	-2.699553000000	-1.230116000000
C	-4.153906000000	-3.800304000000	-0.371440000000
C	-3.849781000000	-2.312898000000	-0.214049000000
H	-4.807007000000	-0.756646000000	-1.418591000000
H	-2.822465000000	-1.938863000000	-0.330712000000
C	-5.692971000000	-3.833407000000	-0.214141000000
H	-6.132560000000	-4.795222000000	-0.519467000000
H	-6.035357000000	-3.587157000000	0.803123000000
C	-5.239622000000	-3.285404000000	-2.496362000000
H	-5.914742000000	-3.989368000000	-3.009261000000
H	-4.942991000000	-2.508446000000	-3.220484000000
C	-4.019661000000	-4.066441000000	-1.905328000000

H	-3.048661000000	-3.721127000000	-2.297506000000
H	-6.988643000000	-2.395150000000	-1.396406000000
H	-3.554714000000	-4.491162000000	0.237851000000
O	-4.143591000000	-5.495702000000	-2.084720000000
C	-3.729400000000	-6.130883000000	-3.240346000000
O	-3.907328000000	-7.331745000000	-3.318399000000
C	-3.068098000000	-5.298552000000	-4.321513000000
H	-3.693545000000	-4.444828000000	-4.629186000000
H	-2.099914000000	-4.897084000000	-3.976092000000
H	-2.889147000000	-5.948502000000	-5.187059000000

P_{NBA}

O	6.062939000000	1.915664000000	-0.504990000000
Si	4.544949000000	1.950583000000	0.112416000000
O	4.364873000000	0.664829000000	1.110748000000
Si	3.301770000000	-0.467013000000	1.645952000000
O	3.285504000000	-1.829532000000	0.718789000000
O	3.385224000000	1.780100000000	-1.080451000000
O	4.209966000000	3.375673000000	0.888951000000
Si	4.690106000000	4.342514000000	2.112284000000
O	3.391361000000	4.878906000000	2.940696000000
Si	2.270978000000	6.086166000000	3.034362000000
O	0.802015000000	5.321563000000	2.835951000000
O	1.805622000000	0.149691000000	1.804201000000
Si	0.883287000000	1.276223000000	2.567939000000
O	-0.637203000000	0.639720000000	2.705613000000
O	3.941716000000	-0.911419000000	3.140159000000
O	5.697524000000	5.536447000000	1.660740000000
Si	5.933038000000	6.760375000000	0.578648000000
O	5.144720000000	8.043676000000	1.361005000000
O	5.350588000000	6.476630000000	-0.920368000000
O	7.561976000000	7.001125000000	0.402653000000
O	5.613637000000	3.452147000000	3.178871000000
Si	5.396712000000	2.417766000000	4.465547000000
O	4.047100000000	1.472361000000	4.371220000000
O	0.815593000000	2.674036000000	1.659196000000
Si	0.176083000000	3.201568000000	0.214654000000
O	-1.386893000000	3.706967000000	0.437799000000
Si	-2.242931000000	4.945034000000	1.136653000000
O	-3.868374000000	4.609521000000	1.047603000000
O	0.184043000000	2.020202000000	-0.932599000000
O	1.121419000000	4.507142000000	-0.120715000000
Si	1.419158000000	5.619244000000	-1.319017000000
O	2.592454000000	6.639528000000	-0.729886000000
O	2.138806000000	4.839328000000	-2.603223000000
O	0.037548000000	6.356609000000	-1.828965000000
O	1.407797000000	1.715276000000	4.064038000000
O	6.798073000000	1.545409000000	4.516049000000
Si	7.816028000000	0.673474000000	3.543731000000
O	6.889599000000	-0.452585000000	2.701395000000
O	8.600632000000	1.561149000000	2.412177000000
Si	9.058851000000	3.091926000000	2.009806000000

O	10.690436000000	3.257117000000	1.850767000000
O	8.343858000000	3.264716000000	0.499854000000
O	8.518487000000	4.220739000000	3.101359000000
O	8.830134000000	-0.065155000000	4.610735000000
O	5.164876000000	3.271323000000	5.866572000000
O	-1.842844000000	6.305634000000	0.227640000000
O	-1.966575000000	5.149390000000	2.749187000000
O	2.517228000000	7.257316000000	1.879825000000
O	2.350172000000	6.855297000000	4.493631000000
H	6.811011000000	2.444271000000	-0.133478000000
H	3.398249000000	2.494681000000	-1.748679000000
H	4.910425000000	-1.095636000000	3.064578000000
H	-0.983223000000	0.611163000000	3.624392000000
H	2.385866000000	1.740660000000	4.240141000000
H	-4.205281000000	4.506638000000	0.140390000000
H	-1.025397000000	5.383612000000	2.952800000000
H	2.939877000000	-1.755470000000	-0.187505000000
H	0.926456000000	4.391719000000	2.518749000000
H	2.203032000000	6.286632000000	5.275611000000
H	5.853999000000	3.923536000000	6.081502000000
H	4.111277000000	0.545320000000	3.994587000000
H	11.174130000000	3.605371000000	2.619067000000
H	8.571915000000	4.079721000000	0.014481000000
H	7.540793000000	4.219085000000	3.193135000000
H	5.242983000000	8.908201000000	0.921248000000
H	8.063327000000	7.067597000000	1.234452000000
H	6.846351000000	-0.272810000000	1.742322000000
H	9.500872000000	-0.661141000000	4.236118000000
H	-0.690975000000	6.402569000000	-1.163500000000
H	1.674015000000	4.935378000000	-3.452029000000
H	1.090755000000	1.700910000000	-1.127975000000
H	-2.168342000000	7.153681000000	0.579590000000
H	3.412319000000	7.692663000000	1.898203000000
H	4.359446000000	6.512339000000	-1.008204000000
H	2.446315000000	6.923370000000	0.214665000000
Ni	-3.201257000000	2.070549000000	5.199566000000
N	-2.477115000000	2.653837000000	6.949874000000
C	-8.002741000000	6.703743000000	5.942722000000
H	-7.537116000000	6.676637000000	6.935009000000
H	-8.687385000000	7.532252000000	5.739807000000
C	-7.754006000000	5.753183000000	5.024849000000
C	-6.827828000000	4.582542000000	5.226040000000
H	-6.445397000000	4.592369000000	6.263675000000
H	-7.396306000000	3.640062000000	5.100576000000
C	-1.023791000000	0.937994000000	6.362589000000
C	-1.420691000000	1.974508000000	7.356272000000
H	-8.245398000000	5.816240000000	4.043331000000
O	-1.815953000000	0.834188000000	5.340999000000
C	-0.744683000000	2.160701000000	8.678664000000
H	-0.541034000000	1.190510000000	9.157991000000
H	0.210223000000	2.699101000000	8.548937000000
H	-1.370931000000	2.762743000000	9.348688000000

C	-3.108148000000	3.600994000000	7.829844000000
C	-2.750433000000	4.970951000000	7.760118000000
C	-4.126626000000	3.145299000000	8.710713000000
C	-3.365405000000	5.858021000000	8.661710000000
C	-4.718789000000	4.082716000000	9.572989000000
C	-4.334218000000	5.435844000000	9.588430000000
H	-3.101088000000	6.919534000000	8.613172000000
H	-5.521064000000	3.745073000000	10.237873000000
C	-4.952215000000	6.407024000000	10.566709000000
H	-4.404740000000	6.401787000000	11.526374000000
H	-4.927997000000	7.438990000000	10.182601000000
H	-5.998873000000	6.144951000000	10.789333000000
C	-4.564939000000	1.671303000000	8.717366000000
H	-4.468450000000	1.317082000000	7.669940000000
C	-1.710097000000	5.454393000000	6.738615000000
H	-1.813275000000	4.792897000000	5.854100000000
C	-6.047190000000	1.495957000000	9.087836000000
C	-6.474957000000	0.824642000000	10.247549000000
C	-7.028346000000	2.018553000000	8.219331000000
C	-7.844455000000	0.688267000000	10.534367000000
H	-5.739622000000	0.394440000000	10.932876000000
C	-8.393609000000	1.884448000000	8.500825000000
H	-6.716448000000	2.547646000000	7.312989000000
C	-8.808274000000	1.216940000000	9.665453000000
H	-8.154104000000	0.160621000000	11.441614000000
H	-9.134960000000	2.299262000000	7.810599000000
H	-9.873540000000	1.107230000000	9.888631000000
C	-3.634848000000	0.781391000000	9.544318000000
C	-3.231525000000	-0.469016000000	9.035119000000
C	-3.192952000000	1.158189000000	10.828068000000
C	-2.401413000000	-1.319444000000	9.782582000000
H	-3.582910000000	-0.784476000000	8.045529000000
C	-2.367869000000	0.308686000000	11.581367000000
H	-3.494302000000	2.126569000000	11.241413000000
C	-1.965126000000	-0.931673000000	11.059456000000
H	-2.102347000000	-2.288131000000	9.370189000000
H	-2.039801000000	0.616255000000	12.579150000000
H	-1.321987000000	-1.593825000000	11.646699000000
C	-1.967421000000	6.882993000000	6.224165000000
C	-3.273463000000	7.273113000000	5.857947000000
C	-0.923070000000	7.805152000000	6.025062000000
C	-3.529142000000	8.539277000000	5.315940000000
H	-4.107410000000	6.583190000000	6.017651000000
C	-1.173857000000	9.075409000000	5.478901000000
H	0.102424000000	7.539531000000	6.292513000000
C	-2.476496000000	9.449299000000	5.122559000000
H	-4.554292000000	8.814914000000	5.048498000000
H	-0.341203000000	9.770737000000	5.335396000000
H	-2.673024000000	10.440927000000	4.703910000000
C	-0.281209000000	5.246364000000	7.249311000000
C	0.661914000000	4.560870000000	6.456666000000
C	0.127332000000	5.736842000000	8.505766000000

C	1.981580000000	4.368014000000	6.907323000000
H	0.365613000000	4.163528000000	5.479344000000
C	1.442671000000	5.551888000000	8.958564000000
H	-0.591253000000	6.268342000000	9.137825000000
C	2.374503000000	4.866438000000	8.161429000000
H	2.705017000000	3.823535000000	6.291682000000
H	1.739752000000	5.944153000000	9.936054000000
H	3.399372000000	4.714946000000	8.513479000000
C	0.090952000000	0.016886000000	6.454139000000
C	0.026667000000	-1.191988000000	5.702576000000
C	1.286877000000	0.294574000000	7.174453000000
C	1.095309000000	-2.076148000000	5.668927000000
H	-0.889478000000	-1.433476000000	5.157840000000
C	2.366005000000	-0.578020000000	7.133859000000
H	1.403780000000	1.233166000000	7.715625000000
C	2.281830000000	-1.766722000000	6.375568000000
H	1.023076000000	-3.006516000000	5.095469000000
H	3.298894000000	-0.346943000000	7.653826000000
O	3.370533000000	-2.569730000000	6.370515000000
H	3.230976000000	-3.325965000000	5.767099000000
C	-4.702114000000	3.370442000000	4.287588000000
H	-3.664763000000	3.756505000000	4.098718000000
H	-4.773655000000	2.976796000000	5.363761000000
C	-5.644169000000	4.604502000000	4.239021000000
H	-5.054490000000	5.518699000000	4.410463000000
H	-6.011275000000	4.671538000000	3.198795000000
C	-4.964758000000	2.241744000000	3.267876000000
C	-6.262824000000	1.400795000000	3.416053000000
C	-4.619489000000	-0.085539000000	3.922353000000
C	-3.874330000000	1.208532000000	3.603263000000
H	-4.868867000000	2.678014000000	2.257460000000
H	-3.015089000000	1.133205000000	2.915423000000
C	-5.928453000000	0.429209000000	4.572641000000
H	-6.681090000000	-0.364380000000	4.702340000000
H	-5.767547000000	0.918683000000	5.548100000000
C	-6.285099000000	0.417882000000	2.212802000000
H	-7.249510000000	-0.113796000000	2.164800000000
H	-6.124049000000	0.926670000000	1.247422000000
C	-5.152047000000	-0.602891000000	2.550997000000
H	-4.350817000000	-0.637770000000	1.794775000000
H	-7.188910000000	1.987769000000	3.516110000000
H	-4.045611000000	-0.856189000000	4.457550000000
O	-5.664227000000	-1.938213000000	2.785217000000
C	-5.849726000000	-2.841986000000	1.759631000000
O	-6.296821000000	-3.938110000000	2.045390000000
C	-5.475172000000	-2.421414000000	0.351757000000
H	-5.966498000000	-1.480054000000	0.056753000000
H	-4.385729000000	-2.272388000000	0.258554000000
H	-5.780064000000	-3.222208000000	-0.333392000000
